# Supplementary material for: Landscape analysis of nutrition services at Primary Health Care Units (PHCUs) in four districts of Ethiopia
Source: PLoS One. 2020 Dec 3;15(12):e0243240. doi: 10.1371/journal.pone.0243240 (PMC7714176; doi:10.1371/journal.pone.0243240)
Supplement: S1 Checklist — (DOCX) [file pone.0243240.s002.docx]

**Observation checklist one: Drug and supplies**

**Date: Observer name:**

**Woreda:**  **Name of Health facility:**

| List of Drugs/supplies | YES | NO | Remark |
| --- | --- | --- | --- |
| Albendazole (400 mg) |  |  |  |
| Mebendazole |  |  |  |
| Vitamin A (50,000 IU) |  |  |  |
| Multivitamin |  |  |  |
| RUTF-F75 (75kcal/100ml) |  |  |  |
| RUTF-F100 (100kcal/100ml) |  |  |  |
| RUSF |  |  |  |
| CSB |  |  |  |
| Multiple Micro nutrient powder |  |  |  |
| Zinc (20mg) |  |  |  |
| ORS |  |  |  |
| Daily rations / TSF |  |  |  |
| RDT |  |  |  |
| Antibiotics |  |  |  |
| Iron folic acid (60mg elemental iron + 0.4µg folic acid) |  |  |  |
| Observers comment |  | | |

**Observation checklist two: Availability of logistics**

Date: ____________________

Woreda: _____________________

| **Check List for Availability of Logistics, and Equipment** | **Immunization** | | | **IMNCI** | | |
| --- | --- | --- | --- | --- | --- | --- |
| [Tick where appropriate] | Available | | Not Ava | Ava | | Non Ava. |
|  | Fun. | Non fun. |  | Fun. | Non fun. |  |
| A. Equipment |  |  |  |  |  |  |
| 1. Baby Weighing scale |  |  |  |  |  |  |
| 2. Adult Weighing Scale |  |  |  |  |  |  |
| 3. Measuring Height/length board |  |  |  |  |  |  |
| 4. thermometer |  |  |  |  |  |  |
| 4. MUAC tape |  |  |  |  |  |  |
| 5. Demonstration equipment |  |  |  |  |  |  |
| 5.1 cooking materials |  |  |  |  |  |  |
| 5.2 oil |  |  |  |  |  |  |
| 5.3 flour |  |  |  |  |  |  |
| 5.4. others |  |  |  |  |  |  |
| 6. Appointment card |  |  |  |  |  |  |
| 7. Child card/record |  |  |  |  |  |  |
| B. Logistics |  |  |  |  |  |  |
| 12. Register |  |  |  |  |  |  |
| 13. Reporting format/Recording format |  |  |  |  |  |  |
| 14. IYCF materials |  |  |  |  |  |  |
| 15. counseling guide |  |  |  |  |  |  |
| 16. IYCF guideline |  |  |  |  |  |  |
| 17 AMYCIN guideline |  |  |  |  |  |  |
| 18. SAM guideline |  |  |  |  |  |  |

Name of Health center/Health post: _____________________

Observer name: ______________________ signature: _________________

**Observational checklist three: Observation of service provision at IMNCI**

Woreda: _____________________________

Name of Health center: _________________

Age of the service provider: _________________

Sex of the provider: _________________
Professional level: _________________

Trained (Yes/No)

Age of the child

| Observation of Activities | **IMNCI** | | | **Remark** |
| --- | --- | --- | --- | --- |
| **A. Explanation of Care /communication** | Y | N | N/A |  |
| 1. When the care was given to the mother or infant, did the medical personnel explain what he/she was doing? |  |  |  |  |
| 2. Allow the mother to ask questions? |  |  |  |  |
| 3. Did mother participate in the discussion? |  |  |  |  |
| 4. Give / Explain the outcome of the examination to the mother? |  |  |  |  |
| 5. Did the health care provider advice the mother on where to seek help in future if she experiences any problems? |  |  |  |  |
| 6. Did the medical personnel informed the mother on the next timing of the care? |  |  |  |  |
| **B. Counselling** |  |  |  |  |
| 8. Advised on Exclusively BF for 6 months? |  |  |  |  |
| 9. Advised on self-care and baby care? |  |  |  |  |
| 10. Advised on initiation of CF at 6 months |  |  |  |  |
| 11. Advised on Continuing frequent, on-demand breastfeeding until two years of age or beyond. |  |  |  |  |
| 12. Advised on the practice responsive feeding |  |  |  |  |
| 13. Advised on starting at six months of age with small amounts of food and increase the quantity as the child gets older, while maintaining frequent breastfeeding. |  |  |  |  |
| 14. Advised on increasing the number of times that the child is fed complementary foods as he/she gets older. |  |  |  |  |
| 15. Advised on gradually increasing food consistency(porridge) and variety as the infant gets older, adapting to the infant’s requirements and abilities. |  |  |  |  |
| 16. Advised given for Low-birth-weight (LBW) infants, including those with very low birth weight (VLBW), should be fed mother’s own milk. |  |  |  |  |
| 17. Advised on feeding frequency and diversity (locally available)  17.1 Grain, roots and tubers  17.2 Legumes and nuts  17.3 Fruits and vegetables  17.4 Animal source foods |  |  |  |  |
| 18. Advised on personal and Food Hygiene (hand washing on before food preparation, after vising toilet, feeding child |  |  |  |  |
| 19. Proper storage of food |  |  |  |  |
| 20. Advised on increasing fluid intake during illness, including more frequent breastfeeding, and encourage the child to eat soft, varied, appetizing, favorite foods. After illness, give food more often than usual and encourage the child to eat more. |  |  |  |  |
| 21. Use available IEC materials and counselling guide while advising the client |  |  |  |  |
| 22. Advised on iodized salt utilization |  |  |  |  |
| 23. Advised on sunlight exposure |  |  |  |  |
| 24. Advice on use of ITN |  |  |  |  |
| 25. Mother was given fliers or brochures at the end of the counseling |  |  |  |  |
| **C. Screening** |  |  |  |  |
| 25. Was the baby weight measured? |  |  |  |  |
| 26. Was the baby height/ length measured ? |  |  |  |  |
| 27. Was the baby MUAC measured? |  |  |  |  |
| 28. Was the baby checked for any nutritional complications(edema, skin infection, ..)? |  |  |  |  |
| 29. Was the client nutritional status correctly classified? |  |  |  |  |
| 30. Was the mother weight/height/MUAC measured ? |  |  |  |  |
| **D. Management** |  |  |  |  |
| 31. Was the client nutritional status managed appropriately(SAM/MAM)(clear)? |  |  |  |  |
| 32. Link to food support, PSNP and routine IYCN |  |  |  |  |
| 33. Referral / linkage for nutritional complications |  |  |  |  |
| 34. Advised for outpatient therapeutic feeding |  |  |  |  |
| 35. diarrhea treatment with zinc and ORS |  |  |  |  |
| 36. Assess, classify illness and treat according to the IMNCI algorithms |  |  |  |  |

| **E. Observations about Essential counseling skills** | | | |
| --- | --- | --- | --- |
| Service provider listens to what the client has to say |  |  |  |
| Service provider used responses and gestures that show interest |  |  |  |
| Service provider let the client talk through her concerns before correcting information |  |  |  |
| Service provider avoided using judging words |  |  |  |
| Service provider recognized and praised what the client is doing correctly |  |  |  |
| Service provider Identified key difficulties (if any) and selects with the client the most important one to work on |  |  |  |
| Service provider discussed options and key difficulties the client raised |  |  |  |
| Service provider recommends and negotiates do-able actions to help the client select the best option to try depending on her context and resources |  |  |  |
| Service provider helped the client agree to try one of the options and asks them to repeat the agreed-upon do-able action |  |  |  |
| Service provider recorded history of the client |  |  |  |

**Observational checklist four: Observation of service provision at Immunization**

Woreda: _____________________________

Name of Health center: _________________

Age of the service provider: _________________

Sex of the provider: _________________
Professional level: _________________

Trained (Yes/No)

Age of the child

|  | Observation of Activities | **Immunization** | | | **Remark** |
| --- | --- | --- | --- | --- | --- |
|  | **A. Explanation of Care /communication** | Y | N | N/A |  |
| 101 | When the care was given to the mother or infant, did the medical personnel explain what he/she was doing? |  |  |  |  |
| 102 | Allow the mother to ask questions? |  |  |  |  |
| 103 | Did mother participate in the discussion? |  |  |  |  |
| 104 | Give / Explain the outcome of the examination to the mother? |  |  |  |  |
| 105 | Did the health care provider advice the mother on where to seek help in future if she experiences any problems? |  |  |  |  |
| 106 | Did the medical personnel informed the mother on the next timing of the care? |  |  |  |  |
|  | **B. Counselling** |  |  |  |  |
| 107 | Advised on Exclusively BF for 6 months? |  |  |  |  |
| 108 | Advised on self-care and baby care? |  |  |  |  |
| 109 | Advised on initiation of CF at 6 months |  |  |  |  |
| 110 | Advised on Continuing frequent, on-demand breastfeeding until two years of age or beyond. |  |  |  |  |
| 111 | Advised on the practice of responsive feeding |  |  |  |  |
| 112 | Advised on starting at six months of age with small amounts of food and increase the quantity as the child gets older, while maintaining frequent breastfeeding. |  |  |  |  |
| 113 | Advised on increasing the number of times that the child is fed complementary foods as he/she gets older. |  |  |  |  |
| 114 | Advised on gradually increasing food consistency and variety as the infant gets older, adapting to the infant’s requirements and abilities. |  |  |  |  |
| 115 | Advised given for Low-birth-weight (LBW) infants, including those with very low birth weight (VLBW), should be fed mother’s own milk. |  |  |  |  |
| 116 | Advised on feeding frequency and diversity (locally available)  16.1 Grain, roots and tubers  16.2 Legumes and nuts  16.3 Fruits and vegetables  16.4 Animal source foods |  |  |  |  |
| 117 | Advised personal and Food Hygiene (hand washing on before food preparation, after vising toilet, feeding child |  |  |  |  |
| 118 | Advised on proper storage of food |  |  |  |  |
| 119 | Advised to increase fluid intake during illness, including more frequent breastfeeding, and encourage the child to eat soft, varied, appetizing, favorite foods. After illness, give food more often than usual and encourage the child to eat more. |  |  |  |  |
| 120 | Advised on iodized salt utilization |  |  |  |  |
| 121 | Advised on sunlight exposure |  |  |  |  |
| 122 | Advised on use of ITN |  |  |  |  |
| 123 | Use available IEC materials and counselling guide while advising the client |  |  |  |  |
| 124 | Mother was given fliers or brochures at the end of the counseling |  |  |  |  |
|  | **C. Screening** |  |  |  |  |
| 125 | Was the baby weight measured? |  |  |  |  |
| 126 | Was the baby height/ length measured ? |  |  |  |  |
| 127 | Was the baby MUAC measured? |  |  |  |  |
| 128 | Was the baby checked for any nutritional complications(edema, skin infection, ..)? |  |  |  |  |
| 129 | Was the client nutritional status correctly classified? |  |  |  |  |
| 130 | Was the mother weight/height/MUAC measured ? |  |  |  |  |

|  | **D. Observations about Essential counseling skills** | | | |
| --- | --- | --- | --- | --- |
| 131 | Service provider listens to what the client has to say |  |  |  |
| 132 | Service provider used responses and gestures that show interest |  |  |  |
| 133 | Service provider let the client talk through her concerns before correcting information |  |  |  |
| 134 | Service provider avoided using judging words |  |  |  |
| 135 | Service provider recognized and praised what the client is doing correctly |  |  |  |
| 136 | Service provider Identified key difficulties (if any) and selects with the client the most important one to work on |  |  |  |
| 137 | Service provider discussed options and key difficulties the client raised |  |  |  |
| 138 | Service provider recommends and negotiates do-able actions to help the client select the best option to try depending on her context and resources |  |  |  |
| 139 | Service provider helped the client agree to try one of the options and asks them to repeat the agreed-upon do-able action |  |  |  |
| 140 | Service provider recorded history of the client |  |  |  |

**Observational checklist five: Observation checklist for service during ANC follow up.**

Date: Observer name:

Woreda: Name of Health facility:

Age of the service provider: _________________

Sex of the provider: _________________

Professional level: _________________

Trained (Yes/No)

**Section one: observation of equipment and materials for ANC service**

| **[Tick where appropriate]** | **Yes** | | **No/NA** | **Remark** |
| --- | --- | --- | --- | --- |
|  | **Fun** | **Non-fun** |  |  |
| **Observation of equipment** |  |  |  |  |
| Weight scale |  |  |  |  |
| Height measuring board |  |  |  |  |
| MUAC tape |  |  |  |  |
| **Observation of registration, guide lines, manuals, SBCC materials** |  |  |  |  |
| ANC registration book/reporting format |  |  |  |  |
| ANC manual |  |  |  |  |
| ANC protocol |  |  |  |  |
| Maternal, newborn, and child health care manual |  |  |  |  |
| Adolescent, maternal and child nutrition guide line |  |  |  |  |
| National guide line for HIV/AIDS and nutrition |  |  |  |  |
| National micronutrient guide line |  |  |  |  |
| IYCF guide line |  |  |  |  |
| Family health guide line |  |  |  |  |
| Flier, brochures, counseling card, poster |  |  |  |  |

**Section two: Observation of service provision and counseling during ANC visit**

ANC visit (Circle where it applies)

Visit 1………………1

Visit 2………………2

Visit 3……………...3

Visit 4……………...4

|  | **Observation of service provision** | **Yes** | **No** | **Remark** |
| --- | --- | --- | --- | --- |
| 101 | Weight of the mother taken |  |  |  |
| 102 | Height of the mother taken |  |  |  |
| 103 | MUAC of the mother taken |  |  |  |
| 104 | Was the clients nutritional status correctly analyzed using MUAC (classified) |  |  |  |
| 105 | ANC provider informed the client about weekly gestational weight gain according to her pre pregnancy weight |  |  |  |
| 106 | ANC provider checked if the woman is keeping track of her gestational weight gain |  |  |  |
| 107 | Checked for pallor |  |  |  |
| 108 | Measured blood pressure |  |  |  |
| 109 | Uterine height measured |  |  |  |
| 110 | Checked her sero-status |  |  |  |
| 111 | Checked the mothers hemoglobin status (first and fourth visit) |  |  |  |
| 112 | The mother status was correctly classified using the hemoglobin result |  |  |  |
| 113 | Mother was provided with IFA supplementation adequate until her next appointment date |  |  |  |
| 114 | Mother was checked for adherence of IFA |  |  |  |
| 115 | Mother was given Mebendazole (Third and fourth visit) |  |  |  |
| 116 | Developed birth preparedness plan (*i.e. identification of place, means of transportation..*)fourth |  |  |  |
|  | **Observation of counselling given for the mother** |  |  |  |
| 117 | Advised to take IFA daily for six month |  |  |  |
| 118 | instruct mothers to take iron after eating meal |  |  |  |
| 119 | Advised mother to avoid tea and coffee at the same time when taking iron |  |  |  |
| 120 | Advised mother on the benefits of IFA |  |  |  |
| 121 | Advised the mother on possible side effect of iron (heartburn, constipation, dark stool) |  |  |  |
| 122 | Advised the mother to store the table on dry and cool place and in a place where children cannot get it |  |  |  |
| 123 | ANC provider informed the client that she should have extra meal/ snack every day |  |  |  |
| 124 | ANC provider advised the client to eat from variety of food groups using locally available food (Grains, white roots and tubers, and plantains, Pulses (beans, peas and lentils, Nuts and seeds, Dairy, Meat, poultry and fish. Eggs, Dark green leafy vegetables, Other vitamin A-rich fruits and vegetables, Other vegetables, Other fruits |  |  |  |
| 125 | ANC provider advised the client the importance of eating variety of food groups |  |  |  |
| 126 | ANC provider informed the client about having seasonal fruits and vegetables everyday |  |  |  |
| 127 | ANC provider informed the client about adequate fluid intake (*at least 8 glasses of fluid everyday*) |  |  |  |
| 128 | ANC provider informed the client about adding iodized salt when serving food (after cooking) |  |  |  |
| 129 | ANC provider informed the client about what to avoid *(i.e. Alcohol, raw/uncooked foods and vegetables,* |  |  |  |
| 130 | ANC provider informed the client about what to limit during pregnancy (*i.e. caffeine, fat )* |  |  |  |
| 131 | ANC provider informed the client about maternal complications of under nutrition during pregnancy |  |  |  |
| 132 | ANC provider informed the client about fetal complications of under nutrition during pregnancy |  |  |  |
| 133 | ANC provider gave information on the importance of early initiation of breast feeding (fourth visit) |  |  |  |
| 134 | ANC provider advised her on importance of feeding colostrum |  |  |  |
| 135 | Advised on Avoidance of pre-lacteal feeding (fourth visit) |  |  |  |
| 136 | Advised to expose the child to direct sun light (fourth visit) |  |  |  |
| 137 | ANC provider gave information on the importance of exclusive breast feeding for six month (fourth visit) |  |  |  |
| 138 | Advised the mother to use treated water for drinking (*i.e. with weha agar, or boiling and filtration..*) |  |  |  |
| 139 | Advised the mother to wash hand after toilet use, and before meal preparation |  |  |  |
| 140 | Advised the mother to keep utensils and cooking material clean |  |  |  |
| 141 | Advised the mother to keep personal hygiene *(i.e. bathing..)* |  |  |  |
| 142 | Advise on ITN use |  |  |  |
| 143 | Use available IEC materials and counseling guide while advising the client |  |  |  |
| 144 | Mother was given fliers or brochures at the end of the counseling |  |  |  |
|  | **Observations about Essential counseling skills** | | |  |
| 145 | ANC provider listens to what the client has to say |  |  |  |
| 146 | ANC provider used responses and gestures that show interest |  |  |  |
| 147 | ANC provider let the client talk through her concerns before correcting information |  |  |  |
| 148 | ANC provider avoided using judging words |  |  |  |
| 149 | ANC provider recognized and praised what the client is doing correctly |  |  |  |
| 150 | ANC provider Identified key difficulties (if any) and selects with the client the most important one to work on |  |  |  |
| 151 | ANC provider discussed options and key difficulties the client raised |  |  |  |
| 152 | ANC provider recommends and negotiates do-able actions to help the client select the best option to try depending on her context and resources |  |  |  |
| 153 | ANC provider helped the client agree to try one of the options and asks them to repeat the agreed-upon do-able action |  |  |  |
| 154 | ANC provider recorded history of the client |  |  |  |
| 155 | Made appointment for the next follow-up visit |  |  |  |

**Observational checklist six: Observation checklist for service during Postnatal care.**

Date: Observer name:

Woreda: Name of Health facility:

Age of the service provider: _________________

Sex of the provider: _________________

Professional level: _________________

Trained (Yes/No)

**Section one: observation of equipment and materials for PNC service**

| **[Tick where appropriate]** | **Yes** | | **No/NA** | **Remark** |
| --- | --- | --- | --- | --- |
|  | **Fun** | **Non-fun** |  |  |
| **Observation on equipment** |  |  |  |  |
| Weight scale |  |  |  |  |
| MUAC tape |  |  |  |  |
| Baby Weighing scale |  |  |  |  |
| Head circumference measuring tape |  |  |  |  |
| **Observation on registration, guide lines, manuals, SBCC materials** |  |  |  |  |
| PNC registration book/reporting format |  |  |  |  |
| PNC manual |  |  |  |  |
| Family planning guide line |  |  |  |  |
| Maternal, newborn, and child health care manual/BEMONC manual? |  |  |  |  |
| Adolescent, maternal and child nutrition guide line |  |  |  |  |
| National guide line for HIV/AIDS and nutrition |  |  |  |  |
| National micronutrient guide line |  |  |  |  |
| IYCF guide line |  |  |  |  |
| Flier, brochures, counseling card, poster |  |  |  |  |

**Section two: Observation of service provision and counseling during Postnatal care**

|  | **Observation of service provided for the mother during delivery** | **Yes** | **No** | **Remark** |
| --- | --- | --- | --- | --- |
| 101 | BP and temperature of the mother |  |  |  |
| 102 | Checked for pallor |  |  |  |
| 103 | Weight of the mother taken |  |  |  |
| 104 | Support initiation of breast feeding within 1 hour |  |  |  |
| 105 | Continued supplementation of micronutrients IFA |  |  |  |
| 106 | Assisted the mother on proper attachment and positioning |  |  |  |
| 107 | Supported the mother to frequently and exclusively breastfed |  |  |  |
| 108 | Mother provided with food as soon as she requested |  |  |  |
| 109 | Delayed cord clamping |  |  |  |
|  | **Observation of service provided for the baby during delivery** |  |  |  |
| 110 | Assessment of general condition, active ,feeding well, frequently of the baby |  |  |  |
| 111 | Birth weight was correctly measured |  |  |  |
| 112 | Baby was correctly classified based on birth weight |  |  |  |
| 113 | Head circumference was correctly measured |  |  |  |
| 114 | frequently and exclusive breast feeding |  |  |  |
|  | **Observation of counseling service provided for the mother during PNC** |  |  |  |
| 115 | \| Client counseled on two extra meal and rest during lactation \| \| --- \| |  |  |  |
| 116 | Advised the mother to eat a diet that is rich in protein and fluid |  |  |  |
| 117 | Advised the mother to eat food rich in iron |  |  |  |
| 118 | Advised mother to avoid all dietary restriction if any |  |  |  |
| 119 | Advised mother to exclusively breastfed up to six month |  |  |  |
| 120 | PNC provider gave information on the importance of exclusive breast feeding for six month |  |  |  |
| 121 | Advised mother to breast fed on demand |  |  |  |
| 122 | Advised mother on correct positioning and attachment during breast feeding |  |  |  |
| 123 | PNC provider advised the client to eat from variety of food groups using locally available food |  |  |  |
| 124 | PNC provider advised the client the importance of eating variety of food groups |  |  |  |
| 125 | PNC provider informed the client about having seasonal fruits and vegetables everyday |  |  |  |
| 126 | PNC provider informed the client about adequate fluid intake (at least 8 glasses of fluid everyday) |  |  |  |
| 127 | PNC provider informed the client about adding iodized salt when serving food (after cooking) |  |  |  |
| 128 | PNC provider informed the client about maternal complications of under nutrition during lactation |  |  |  |
| 129 | Advised the mother to use treated water for drinking (*i.e. with weha agar, or boiling and filtration..*) |  |  |  |
| 130 | Counseling on safe disposal of potentially infectious soiled pads or other materials |  |  |  |
| 131 | Advised the mother to wash hand after toilet use, and before meal preparation, after cleaning baby, before feeding ,before breast feeding |  |  |  |
| 132 | Advised the mother to keep utensils and cooking material clean |  |  |  |
| 133 | Advised the mother to keep personal hygiene *(i.e. bathing..)* |  |  |  |
| 134 | Advised the mother on family planning |  |  |  |
| 135 | Advised on ITN use |  |  |  |
| 136 | PNC provider used available IEC materials and counselling guide while advising the client |  |  |  |
| 137 | Mother was given fliers or brochures at the end of the counseling |  |  |  |
|  | **Observations about Essential counseling skills** | | | |
| 138 | PNC provider listens to what the client has to say |  |  |  |
| 139 | PNC provider used responses and gestures that show interest |  |  |  |
| 140 | PNC provider let the client talk through her concerns before correcting information |  |  |  |
| 141 | PNC provider avoided using judging words |  |  |  |
| 142 | PNC provider recognized and praised what the client is doing correctly |  |  |  |
| 143 | PNC provider Identified key difficulties (if any) and selects with the client the most important one to work on |  |  |  |
| 144 | PNC provider discussed options and key difficulties the client raised |  |  |  |
| 145 | PNC provider recommends and negotiates do-able actions to help the client select the best option to try depending on her context and resources |  |  |  |
| 146 | PNC provider helped the client agree to try one of the options and asks them to repeat the agreed-upon do-able action |  |  |  |
| 147 | PNC provider recorded history of the client |  |  |  |
| 148 | Made appointment for the next follow-up visit |  |  |  |

**ምልከታ ዝርዝር 1: የመድሀኒት አቅርቦቶች ምልከታ ዝርዝር**

**ቀን: የታዛቢ ስም:**

**ወረዳ :**  **የጤና ተቋም ስም:**

| የመድኃኒቶች / አቅርቦቶች ዝርዝር | ይገኛል | አይገኝም | ማስታወሻ |
| --- | --- | --- | --- |
| አልቤንዳዞል |  |  |  |
| መበንዳዞል |  |  |  |
| ቫይታሚን ኤ |  |  |  |
| መልቲ ቫይታሚን |  |  |  |
| RUTF-F75 |  |  |  |
| RUTF-F100 |  |  |  |
| RUSF |  |  |  |
| የበቆሎ ሶያ ድብልቅ ( CSB) |  |  |  |
| Multiple Micro Nutrient powder (ደስታ) |  |  |  |
| ዚንክ |  |  |  |
| ኦ.አር.ኤስ ( ORS) |  |  |  |
| Daily rations / TSF |  |  |  |
| አር.ዲ.ቲ. (RDT) |  |  |  |
| አንቲባዮቲክስ |  |  |  |
| የአይረን ፎሊክ አሲድ |  |  |  |
| ታዛቢዎች አስተያየት |  | | |

**ምልከታ ዝርዝር 2: ለሎጅስቲክስ ፣ ለመሣሪያዎች እና የማረጋገጫ ዝርዝር**

ቀን:____________________

ወረዳ: _____________________

ጤና ጣቢያ /ጤና ኬላ: _____________________

| **ለሎጅስቲክስ ፣ ለመሣሪያዎች እና የማረጋገጫ ዝርዝር** | **የክትባት አገልግሎት** | | | **የተቀናጀ ጨቅላ ህፃን እና የህፃናት ኢንፌክሽን አገልግሎት (IMNCI)** | | |
| --- | --- | --- | --- | --- | --- | --- |
| **[በተገቢው ቦታ ላይ ምልክት ያድርጉ]** | **ይገኛል** | | **አይገኝም** | **ይገኛል** | | **አይገኝም** |
|  | **ይሠራል** | **አይሰራም** |  | **ይሠራል** | **አይሰራም** |  |
| **ሀ. መሣሪያዎች** |  |  |  |  |  |  |
| 1. የህፃን ክብደት ሚዛን |  |  |  |  |  |  |
| 2. የአዋቂዎች ክብደት ሚዛን |  |  |  |  |  |  |
| 3. የመለኪያ ቁመት / ርዝመት ሰሌዳ |  |  |  |  |  |  |
| 4. የሙቀት መለኪያ |  |  |  |  |  |  |
| 4. የመካከለኛ የላይኛው ክንድ ክበብ መለኪያ |  |  |  |  |  |  |
| 5. የማሳያ መሳሪያዎች |  |  |  |  |  |  |
| 5.1 የማብሰያ ቁሳቁሶች |  |  |  |  |  |  |
| 5.2 ዘይት |  |  |  |  |  |  |
| 5.3 ዱቄት |  |  |  |  |  |  |
| 5.4. ሌሎች |  |  |  |  |  |  |
| 6. የቀጠሮ ካርድ |  |  |  |  |  |  |
| 7. የልጆች ካርድ / መዝገብ |  |  |  |  |  |  |
| ለ. ሎጅስቲክስ |  |  |  |  |  |  |
| 12. መዝገብ |  |  |  |  |  |  |
| 13. የሪፖርት ቅርጸት / መዝገብ ቅርጸት |  |  |  |  |  |  |
| 14. የ IYCF ቁሳቁሶች |  |  |  |  |  |  |
| 15. የምክር መመሪያ |  |  |  |  |  |  |
| 16. የIYCF መመሪያ |  |  |  |  |  |  |
| 17. የ AMYCIN መመሪያ |  |  |  |  |  |  |
| 18. የ ከባድ አጣዳፊ የተመጣጠነ ምግብ እጥረት  (SAM) መመሪያ |  |  |  |  |  |  |

የታዛቢ ስም: ______________________ ፊርማ: _________________

**ምልከታ ዝርዝር 3: የተቀናጅ ጨቅላ ህፃን እና የህፃናት ኢንፌክሽን ህክምና አገልግሎት የክትትል ማረጋገጫ**

ቀን: ________________

ወረዳ: _____________________________

የጤና ጣቢያ ስም_________________

የአገልግሎት አቅራቢ ዕድሜ_________________

የአገልግሎት አቅራቢ ፆታ_________________
የሙያ ደረጃ_________________

የአገልግሎት ሰጪው ስልጠና ውስዷል (አዎ/አይ) ________________

የህፃኑ እድሜ ________________

|  | **የእንቅስቃሴዎች ምልከታ** | | **የተቀናጀ ጨቅላ ህፃን እና የህፃናት ኢንፌክሽን ህክምና አገልግሎት (IMNCI)** | | | | | **አስተያየት** |  |
| --- | --- | --- | --- | --- | --- | --- | --- | --- | --- |
|  | **ስለ እንክብካቤ / ግንኙነት ማብራሪያ** | | **አዎ** | | | **አይ** | **ተፈፃሚ የማይሆን** |  |  |
| 101 | እንክብካቤ ለእናት ወይም ለህፃን በሚሰጥበት ጊዜ የሕክምና ባልደረቦቹ ምን እያደረገ እንደነበረ አስረድተዋል? | |  | | |  |  |  |  |
| 102 | እናት ጥያቄዎችን እንድትጠይቅ ይፍቀዱለት? | |  | | |  |  |  |  |
| 103 | እናት በውይይት ተሳትፋለች? | |  | | |  |  |  |  |
| 104 | የምርመራውን ውጤት ለእናቱ ሰጡ / አስረዱ? | |  | | |  |  |  |  |
| 105 | የጤና እንክብካቤ አቅራቢዎች እናት ችግር ካጋጠማት ወደፊት የት መጠየቅ እንዳለባት ምክር ሰጡ? | |  | | |  |  |  |  |
| 106 | የሕክምና ባልደረቦቹ በሚቀጥለው የእንክብካቤ ጊዜ ለእናቱ አሳውቀዋል | |  | | |  |  |  |  |
|  | **ምክር** | |  | | |  |  |  |  |
| 107 | ለመጀመሪያ ስድስት ወራት ጡት ብቻ መመገብ እንዳለባት መክሯታል | |  | | |  |  |  |  |
| 108 | በራስ እንክብካቤ እና በሕፃን እንክብካቤ ላይ ምክር ተሰጥቷል | |  | | |  |  |  |  |
| 109 | በ 6 ወር ላይተጨማሪ ምግብ እንድታስጀምር ምክር ተሰጥቷል | |  | | |  |  |  |  |
| 110 | እስከ 2 ዓመት ዕድሜ ድረስ ወይም ከዚያ በላይ በተከታታይ እና በልጅ ፍላጎት መሰረት ጡት ማጥባት እንድትቀጥል መክሯታል | |  | | |  |  |  |  |
| 111 | በተግባር ምላሽ ሰጪ (responsive feeding )አመጋገብ ላይ ተመክሯል | |  | | |  |  |  |  |
| 112 | ከ 6 ወር እድሜው ጀምሮ በትንሽ ምግብ በመጀመር እና ህፃኑ እያደገ ሲሄድ ብዛቱን እንዲጨምር ማድረግ ላይ፣ እንዲሁም ጡት ማጥባት እንድትቀጥል መክሯል | |  | | |  |  |  |  |
| 113 | ህፃኑ እያደገ ሲሄድ ህፃኑ በቀን ውስጥ ተጨማሪ ምግብ የሚመብበትን ቁጥር መጠን መጨመር ላይ ይመክራል | |  | | |  |  |  |  |
| 114 | የሕፃናትን ፍላጎት እና ችሎታዎች በማጣጣም ህፃኑ ዕድሜው እየገፋ ሲሄድ ቀስ በቀስ የምግብ ውፍረት እና አይነት እንዲጨምር ይመክራል | |  | | |  |  |  |  |
| 115 | ዝቅተኛ ክብደት ላላቸው እንዲሁም በጣም ዝቅተኛ ክብደት ላላቸውን ሕፃናት የሚሰጥ ምክር (የእናት ጡት ወተት እንዲመገቡ ለእናቶች የተሰጠ ምክር) | |  | | |  |  |  |  |
| 116 | የመመገቢያ ድግግሞሽ እና የምግብ አይነትን በተመለከተ የተሰጠ ምክር ​​(በአከባቢው የሚገኝ የምግብ አይነት)  16.1 እህል ፣ ሥሮች እና ሀረጎች  16.2 ጥራጥሬዎች እና ፍሬዎች  16.3 ፍራፍሬዎች እና አትክልቶች  16.4 የእንስሳት ምንጭ ምግቦች | |  | | |  |  |  |  |
| 117 | በግል እና በምግብ ንፅህና ዙሪያ መክራል (ከምግብ ዝግጅት በፊት እጅ መታጠብ ፣ መፀዳጃ ቤት በኋላ እጅ መታጠብ፣ ህፃን ከመመገብ በፊት እጅ መታጠብ) | |  | | |  |  |  |  |
| 118 | ስለ ትክክለኛ ምግብ አቀማመጥ ላይ ምክር ተሰጥቷል | |  | | |  |  |  |  |
| 119 | በሕመም ወቅት ፈሳሽ መውሰድ እንዲጨምር ምክር ይሰጣል ፣ ብዙ ጊዜ ጡት ማጥባትን ያጠቃልላል ፣ እና ልጁ ለስላሳ ፣ የተለያዩ እና ተወዳጅ የሆኑ ምግቦችን እንዲመገብ ያበረታቱ ፡፡ ከታመመ በኋላ ከተለመደው በላይ ብዙውን ጊዜ ምግብ ይሰጡ እና ህፃኑ ብዙ እንዲበላ ያበረታቱ ፡፡ | |  | | |  |  |  |  |
| 120 | በአዮዲን ጨው አጠቃቀም ላይ ምክር ተሰጥቷል | |  | | |  |  |  |  |
| 121 | ልጅን ለፀሐይ ብርሃን መጋለጥ ላይ ምክር ተሰጥቷል | |  | | |  |  |  |  |
| 122 | በፀረ-ተባይ መድሃኒት የታከመ የአልጋ-አጎበር አጠቃቀም ላይ ምክር ተሰጥቷል | |  | | |  |  |  |  |
| 123 | ደንበኛውን በሚመክሩበት ጊዜ የሚገኙትን የ የማስተማሪያ እና የተግባቦት መሰሪያዎችን (IEC) እና የምክር መመሪያዎችን ተጠቅሟል | |  | | |  |  |  |  |
| 124 | በምክክሩ ማብቂያ ላይ እናትየው በራሪ ወረቀቶች ወይም  ብሮሹሮች ተሰታለች | |  | | |  |  |  |  |
|  | **ማጣሪያ** | |  | | |  |  |  |  |
| 125 | የሕፃኑ ክብደት ተለክቷል? | |  | | |  |  |  |  |
| 126 | የሕፃኑ ቁመት / ርዝመት ተለክቷል? | |  | | |  |  |  |  |
| 127 | ህፃኑ የመካከለኛ የላይኛው ክንድ ክበብ (MUAC) ተለክቷል? | |  | | |  |  |  |  |
| 128 | ህፃኑ ለማንኛውም የአመጋገብ ችግሮች (እብጠት ፣ የቆዳ ኢንፌክሽን ፣ ..) ምርመራ ተደረጎለታል? | |  | | |  |  |  |  |
| 129 | የደንበኛው የስነ-ምግብ ሁኔታ/nutritional status/ በትክክል ተመድቧል? | |  | | |  |  |  |  |
| 130 | የእናት ክብደት / ቁመት / የመካከለኛ የላይኛው ክንድ ክበብ ተለካ? | |  | | |  |  |  |  |
|  | **አስተዳደር** | |  | | |  |  |  |  |
| 131 | የደንበኛው የአመጋገብ ሁኔታ(nutritional status) በተገቢው ሁኔታ ታክሞ ነበር (ከባድ አጣዳፊ የተመጣጠነ ምግብ እጥረት / መካከለኛ አጣዳፊ የተመጣጠነ ምግብ እጥረት) (ግልፅ)? | |  | | |  |  |  |  |
| 132 | ከምግብ ድጋፍ ፣ PSNP. እና መደበኛ IYCN ጋር ተገናኝቷል | |  | | |  |  |  |  |
| 133 | ለምግብ ችግሮች ውስብስብ / ሪፈራል / ትስስር ተደርጓል | |  | | |  |  |  |  |
| 134 | የተመላላሽ ታካሚ ቴራፒዩቲካል መመገብ ላይ ምክር ተሰቷል | |  | | |  |  |  |  |
| 135 | ተቅማጥ በዚንክ እና በኦ.አር.ኤስ የማከም ስራ ተሰርቷል | |  | | |  |  |  |  |
| 136 | በ IMNCI ስልተ ቀመሮች ምርመራ፣ ምደባና ህክምና ተሰቷል? | |  | | |  |  |  |  |
|  | **ስለ አስፈላጊ የምክር ችሎታ ምልከታዎች** | | | | | | | | |
| 137 | አገልግሎት ሰጪው ደንበኛው የሚናገረውን ያዳምጣል |  | |  |  | | | | |
| 138 | አገልግሎት ሰጪው ፍላጎትን የሚያሳዩ ምላሾችን እና ምልክቶችን ተጠቅሟል |  | |  |  | | | | |
| 139 | አገልግሎት ሰጭው መረጃውን ከማስተካከሉ (ከማረሙ) በፊት ደንበኛዋ ያሳሰባት ነገር እንድትናገር የፈቅድላታል |  | |  |  | | | | |
| 140 | አገልግሎት ሰጪው የመፍረድ ቃላትን ከመጠቀም ተቆጥቧል |  | |  |  | | | | |
| 141 | አገልግሎት ሰጭው ደንበኛው በትክክል የሚሰራውን ነገር ለይቶ አውቆ አመስግኗል |  | |  |  | | | | |
| 142 | የአገልግሎት አቅራቢ ቁልፍ ችግሮችን (ካለ) ለይቶ ከደንበኛው ጋር አብሮ ለመስራት በጣም አስፈላጊ የሆነውን ችግር መርጧል |  | |  |  | | | | |
| 143 | አገልግሎት ሰጪው ደንበኛው ባነሳቸው አማራጮች እና ቁልፍ ችግሮች ላይ ተወያይቷል |  | |  |  | | | | |
| 144 | የአገልግሎት አቅራቢ በደንበኛው ሁኔታ እና ግብዓት ላይ በመመርኮዝ በጣም ጥሩውን አማራጭ እንዲመርጥ ለመርዳት የሚችሉ እርምጃዎችን ይመክራል እንዲሁም ይደራደራል |  | |  |  | | | | |
| 145 | አገልግሎት ሰጪው ደንበኛው ከአማራጮቹ ውስጥ አንዱን ለመሞከር እንዲስማማ ረድቷታል እንዲሁም የተስማሙትን እርምጃ እንዲደግሙላቸው ጠይቋል |  | |  |  | | | | |
| 146 | የአገልግሎት አቅራቢው የደንበኛውን ታሪክ መዝግቧል |  | |  |  | | | | |

**ምልከታ ዝርዝር 4: በክትባት ግዜ የአገልግሎት የክትትል ማረጋገጫ ዝርዝር** ቀን: ________________

ወረዳ: _____________________________

የጤና ጣቢያ ስም_________________

የአገልግሎት አቅራቢ ዕድሜ_________________

የአገልግሎት አቅራቢ ፆታ_________________
የሙያ ደረጃ_________________

የአገልግሎት ሰጪው ስልጠና ውስዷል (አዎ/አይ) ________________

የህፃኑ እድሜ ________________

|  | **የእንቅስቃሴዎች ምልከታ** | | **የክትባት አገልግሎት** | | | | | **አስተያየት** |  |
| --- | --- | --- | --- | --- | --- | --- | --- | --- | --- |
|  | **ስለ እንክብካቤ / ግንኙነት ማብራሪያ** | | **አዎ** | | | **አይ** | **ተፈፃሚ የማይሆን** |  |  |
| 101 | እንክብካቤ ለእናት ወይም ለህፃን በሚሰጥበት ጊዜ የሕክምና ባልደረቦቹ ምን እያደረገ እንደነበረ አስረድተዋል? | |  | | |  |  |  |  |
| 102 | እናት ጥያቄዎችን እንድትጠይቅ ይፍቀዱለት? | |  | | |  |  |  |  |
| 103 | እናት በውይይት ተሳትፋለች? | |  | | |  |  |  |  |
| 104 | የምርመራውን ውጤት ለእናቱ ሰጡ / አስረዱ? | |  | | |  |  |  |  |
| 105 | የጤና እንክብካቤ አቅራቢዎች እናት ችግር ካጋጠማት ወደፊት የት መጠየቅ እንዳለባት ምክር ሰጡ? | |  | | |  |  |  |  |
| 106 | የሕክምና ባልደረቦቹ በሚቀጥለው የእንክብካቤ ጊዜ ለእናቱ አሳውቀዋል | |  | | |  |  |  |  |
|  | **ምክር** | |  | | |  |  |  |  |
| 107 | ለመጀመሪያ ስድስት ወራት ጡት ብቻ መመገብ እንዳለባት መክሯታል | |  | | |  |  |  |  |
| 108 | በራስ እንክብካቤ እና በሕፃን እንክብካቤ ላይ ምክር ተሰጥቷል | |  | | |  |  |  |  |
| 109 | በ 6 ወር ላይተጨማሪ ምግብ እንድታስጀምር ምክር ተሰጥቷል | |  | | |  |  |  |  |
| 110 | እስከ 2 ዓመት ዕድሜ ድረስ ወይም ከዚያ በላይ በተከታታይ እና በልጅ ፍላጎት መሰረት ጡት ማጥባት እንድትቀጥል መክሯታል | |  | | |  |  |  |  |
| 111 | በተግባር ምላሽ ሰጪ (responsive feeding )አመጋገብ ላይ ተመክሯል | |  | | |  |  |  |  |
| 112 | ከ 6 ወር እድሜው ጀምሮ በትንሽ ምግብ በመጀመር እና ህፃኑ እያደገ ሲሄድ ብዛቱን እንዲጨምር ማድረግ ላይ፣ እንዲሁም ጡት ማጥባት እንድትቀጥል መክሯል | |  | | |  |  |  |  |
| 113 | ህፃኑ እያደገ ሲሄድ ህፃኑ በቀን ውስጥ ተጨማሪ ምግብ የሚመብበትን ቁጥር መጠን መጨመር ላይ ይመክራል | |  | | |  |  |  |  |
| 114 | የሕፃናትን ፍላጎት እና ችሎታዎች በማጣጣም ህፃኑ ዕድሜው እየገፋ ሲሄድ ቀስ በቀስ የምግብ ውፍረት እና አይነት እንዲጨምር ይመክራል | |  | | |  |  |  |  |
| 115 | ዝቅተኛ ክብደት ላላቸው እንዲሁም በጣም ዝቅተኛ ክብደት ላላቸውን ሕፃናት የሚሰጥ ምክር (የእናት ጡት ወተት እንዲመገቡ ለእናቶች የተሰጠ ምክር) | |  | | |  |  |  |  |
| 116 | የመመገቢያ ድግግሞሽ እና የምግብ አይነትን በተመለከተ የተሰጠ ምክር ​​(በአከባቢው የሚገኝ የምግብ አይነት)  16.1 እህል ፣ ሥሮች እና ሀረጎች  16.2 ጥራጥሬዎች እና ፍሬዎች  16.3 ፍራፍሬዎች እና አትክልቶች  16.4 የእንስሳት ምንጭ ምግቦች | |  | | |  |  |  |  |
| 117 | በግል እና በምግብ ንፅህና ዙሪያ መክራል (ከምግብ ዝግጅት በፊት እጅ መታጠብ ፣ መፀዳጃ ቤት በኋላ እጅ መታጠብ፣ ህፃን ከመመገብ በፊት እጅ መታጠብ) | |  | | |  |  |  |  |
| 118 | ስለ ትክክለኛ ምግብ አቀማመጥ ላይ ምክር ተሰጥቷል | |  | | |  |  |  |  |
| 119 | በሕመም ወቅት ፈሳሽ መውሰድ እንዲጨምር ምክር ይሰጣል ፣ ብዙ ጊዜ ጡት ማጥባትን ያጠቃልላል ፣ እና ልጁ ለስላሳ ፣ የተለያዩ እና ተወዳጅ የሆኑ ምግቦችን እንዲመገብ ያበረታቱ ፡፡ ከታመመ በኋላ ከተለመደው በላይ ብዙውን ጊዜ ምግብ ይሰጡ እና ህፃኑ ብዙ እንዲበላ ያበረታቱ ፡፡ | |  | | |  |  |  |  |
| 120 | በአዮዲን ጨው አጠቃቀም ላይ ምክር ተሰጥቷል | |  | | |  |  |  |  |
| 121 | ልጅን ለፀሐይ ብርሃን መጋለጥ ላይ ምክር ተሰጥቷል | |  | | |  |  |  |  |
| 122 | በፀረ-ተባይ መድሃኒት የታከመ የአልጋ-አጎበር አጠቃቀም ላይ ምክር ተሰጥቷል | |  | | |  |  |  |  |
| 123 | ደንበኛውን በሚመክሩበት ጊዜ የሚገኙትን የ የማስተማሪያ እና የተግባቦት መሰሪያዎችን (IEC) እና የምክር መመሪያዎችን ተጠቅሟል | |  | | |  |  |  |  |
| 124 | በምክክሩ ማብቂያ ላይ እናትየው በራሪ ወረቀቶች ወይም  ብሮሹሮች ተሰታለች | |  | | |  |  |  |  |
|  | **ማጣሪያ** | |  | | |  |  |  |  |
| 125 | የሕፃኑ ክብደት ተለክቷል? | |  | | |  |  |  |  |
| 126 | የሕፃኑ ቁመት / ርዝመት ተለክቷል? | |  | | |  |  |  |  |
| 127 | ህፃኑ የመካከለኛ የላይኛው ክንድ ክበብ (MUAC) ተለክቷል? | |  | | |  |  |  |  |
| 128 | ህፃኑ ለማንኛውም የአመጋገብ ችግሮች (እብጠት ፣ የቆዳ ኢንፌክሽን ፣ ..) ምርመራ ተደረጎለታል? | |  | | |  |  |  |  |
| 129 | የደንበኛው የስነ-ምግብ ሁኔታ/nutritional status/ በትክክል ተመድቧል? | |  | | |  |  |  |  |
| 130 | የእናት ክብደት / ቁመት / የመካከለኛ የላይኛው ክንድ ክበብ ተለካ? | |  | | |  |  |  |  |
|  | **ስለ አስፈላጊ የምክር ችሎታ ምልከታዎች** | | | | | | | | |
| 131 | አገልግሎት ሰጪው ደንበኛው የሚናገረውን ያዳምጣል |  | |  |  | | | | |
| 132 | አገልግሎት ሰጪው ፍላጎትን የሚያሳዩ ምላሾችን እና ምልክቶችን ተጠቅሟል |  | |  |  | | | | |
| 133 | አገልግሎት ሰጭው መረጃውን ከማስተካከሉ (ከማረሙ) በፊት ደንበኛዋ ያሳሰባት ነገር እንድትናገር የፈቅድላታል |  | |  |  | | | | |
| 134 | አገልግሎት ሰጪው የመፍረድ ቃላትን ከመጠቀም ተቆጥቧል |  | |  |  | | | | |
| 135 | አገልግሎት ሰጭው ደንበኛው በትክክል የሚሰራውን ነገር ለይቶ አውቆ አመስግኗል |  | |  |  | | | | |
| 136 | የአገልግሎት አቅራቢ ቁልፍ ችግሮችን (ካለ) ለይቶ ከደንበኛው ጋር አብሮ ለመስራት በጣም አስፈላጊ የሆነውን ችግር መርጧል |  | |  |  | | | | |
| 137 | አገልግሎት ሰጪው ደንበኛው ባነሳቸው አማራጮች እና ቁልፍ ችግሮች ላይ ተወያይቷል |  | |  |  | | | | |
| 138 | የአገልግሎት አቅራቢ በደንበኛው ሁኔታ እና ግብዓት ላይ በመመርኮዝ በጣም ጥሩውን አማራጭ እንዲመርጥ ለመርዳት የሚችሉ እርምጃዎችን ይመክራል እንዲሁም ይደራደራል |  | |  |  | | | | |
| 139 | አገልግሎት ሰጪው ደንበኛው ከአማራጮቹ ውስጥ አንዱን ለመሞከር እንዲስማማ ረድቷታል እንዲሁም የተስማሙትን እርምጃ እንዲደግሙላቸው ጠይቋል |  | |  |  | | | | |
| 140 | የአገልግሎት አቅራቢው የደንበኛውን ታሪክ መዝግቧል |  | |  |  | | | | |

**ምልከታ ዝርዝር 5: በኤኤንሲ/ቅድመ ወሊድ ክትትል ወቅት የአገልግሎት ምልከታ ዝርዝር**

ቀን: የታዛቢ ስም:

ወረዳ: የጤና ተቋም ስም:

የአገልግሎት ሰጪው ዕድሜ: _________________

የአገልግሎት ሰጪው ጾታ: _________________

የአገልግሎት ሰጪው የሙያ ደረጃ: _________________

የአገልግሎት ሰጪው ስልጠና ውስዷል (አዎ/አይ)

**ክፍል አንድ፥ የኤኤንሲ አገልግሎት መስጫ የመሣሪያዎች እና ቁሳቁሶች ምልከታ**

| **[በተገቢው ቦታ ላይ ምልክት ያድርጉ]** | **አዎ** | | **አይ** | **ማስታወሻ** |
| --- | --- | --- | --- | --- |
|  | **ይሰራል** | **አይሰራም** |  |  |
| **የአገልግሎት መስጫ መሣሪያዎች ምልከታ** |  |  |  |  |
| የአዋቂ የክብደት ሚዛን |  |  |  |  |
| የቁመት የመለኪያ |  |  |  |  |
| የMUAC ቴፕ |  |  |  |  |
| **የመዝገብ ፣ የመመሪያ ፣ የማኑዋሎች ፣ የማህበረሰብ ባህሪ ለውጥ ተግባቦት ቁሳቁሶች ምልከታ** |  |  |  |  |
| የኤኤንሲ ክትትል መዝገብ |  |  |  |  |
| የኤኤንሲ መመሪያ ማንዋል |  |  |  |  |
| የኤኤንሲ ፕሮቶኮል |  |  |  |  |
| የእናቶች ፣ አራስ እና የህፃናት ጤና አጠባበቅ መመሪያ |  |  |  |  |
| የአፍላ ወጣት የእናቶች እና የልጆች አመጋገብ መመሪያ |  |  |  |  |
| የብሔራዊ የኤች.አይ.ቪ / ኤድስ አመጋገብ መመሪያ |  |  |  |  |
| የብሔራዊ የማይክሮ ንጥረ-ነገር መመሪያ |  |  |  |  |
| የ IYCF መመሪያ |  |  |  |  |
| የቤተሰብ ጤና መመሪያ |  |  |  |  |
| ፍላየር ፣ ብሮሹሮች ፣ የምክር ካርድ ፣ ፖስተር |  |  |  |  |

**ክፍል ሁለት፥ በኤኤንሲ ጉብኝት ወቅት የአገልግሎት አቅርቦት እና የምክር አገልግሎት ምልከታ**

የእርግዝና ክትትል ግዜ (አንዱን በማክበብ ይምረጡ)

የመጀመሪያ ጉብኝት………………..1

ሁለተኛ ጉብኝት……………………...2

ሶስተኛ ጉብኝት………………………3

አራተኛ ጉብኝት……………………….4

|  | **የአገልግሎት አቅርቦት ምልከታ** | **አዎ** | **አይ** | **ማስታወሻ** |
| --- | --- | --- | --- | --- |
| 101 | የእናት ክብደት ተለክቷል |  |  |  |
| 102 | የእናት ቁመት ተለክቷል |  |  |  |
| 103 | የእናት MUAC ተለክቷል |  |  |  |
| 104 | የደንበኛው የስነ-ምግብ ሁኔታ/nutritional status/ የ MUAC ልኬትን በመጠቀም በትክክል ተመድቧል? |  |  |  |
| 105 | የኤኤንሲ አገልግሎት ሰጬው በቅድመ እርግዝና ክብደቷ መሠረት ሳምንታዊ የእርግዝና ክብደት መጨመር ለደንበኛው አሳውቋል |  |  |  |
| 106 | የኤኤንሲ አገልግሎት ሰጪ እናትየው በእርግዝና ውቅት መጨመር ያለባትን ክብደቷን እየተከታተለች መሆኗን አጣርቷል |  |  |  |
| 107 | የድም ማነስ ምልክቶችን አጣርቷል |  |  |  |
| 108 | የደም ግፊት ተለክቷል |  |  |  |
| 109 | የማህፀን ቁመት ተለክቷል |  |  |  |
| 110 | የHIV ሁኔታ ተለክቷል |  |  |  |
| 111 | እናቶችን የሂሞግሎቢንን ሁኔታ አጣርቷል (የመጀመሪያ እና አራተኛ ጉብኝት) |  |  |  |
| 112 | የሂሞግሎቢንን ውጤት በመጠቀም የእናት ሁኔታ በትክክል ተመድቧል |  |  |  |
| 113 | እናትየው እስከ ቀጣዩ ቀጠሮ ድረስ የሚበቃ በቂ የአይረን ፎሊክ እንክብል ተሰቷታል |  |  |  |
| 114 | እናትየው የአይረን ፎሊክ እንክብል በተገቢው ሁኔታ እየወሰደች መሆኑን አጣርቷል |  |  |  |
| 115 | እናትየው መቤንዳዞል ተሰቷታል (ሦስተኛው እና አራተኛው ጉብኝት) |  |  |  |
| 116 | የወሊድ ዝግጁነት ዕቅድ አውጥተዋል (*ማለትም የቦታ መለየት ፣ የመጓጓዣ መንገዶች..*) አራተኛው ጉብኝት |  |  |  |
|  | **ለእናት የተሰጠ የምክር አገልግሎት ምልከታ** |  |  |  |
| 117 | IFA በየቀኑ ለስድስት ወር ያህል እንዲወስድ መክሯል |  |  |  |
| 118 | ምግብ ከተመገቡ በኋላ IFA እንዲወስዱ መክሯል |  |  |  |
| 119 | IFA በሚወስዱበት ጊዜ ሻይ እና ቡና በአንድ ጊዜ እንዳይወስዱ መክሯል |  |  |  |
| 120 | IFA ያለውን ጥቅሞች መክሯል |  |  |  |
| 121 | IFA ሊያስከትል የሚችለውን የጎንዮሽ ጉዳት ላይ መክሯል (የደረት ማቃጠል፣የሆድ ድርቀት፣ የሰገራ መጠቆር) |  |  |  |
| 122 | የ IFA እንክብሉን በደረቅ እና በቀዝቃዛ ቦታ እና ልጆች ሊያገኙት በማይችሉበት ቦታ ላይ እንዲያስቀምጡ መክሯል |  |  |  |
| 123 | የኤኤንሲ አገልግሎት ሰጪ ለደንበኛው በየቀኑ ተጨማሪ ምግብ / መክሰስ መመገብ እንደሚኖርባት አሳወቋል |  |  |  |
| 124 | የኤኤንሲ አገልግሎት አቅራቢ ደንበኛው በአካባቢ የሚገኘውን ምግብ በመጠቀም ከተለያዩ የምግብ ቡድኖች እንድትመገብ መክሯል (እህሎች ፣ ነጭ ሥሮች, ባቄላ ፣ አተር እና ምስር, ለውዝ ፣ የወተት ተዋጽኦ ፣ ሥጋ ፣ የዶሮ እርባታ እና ዓሳ. እንቁላል ፣ ጥቁር አረንጓዴ ቅጠላማ አትክልቶች ፣ ሌሎች በቫይታሚን ኤ የበለፀጉ ፍራፍሬዎች እና አትክልቶች ፣ ሌሎች አትክልቶች ፣ ሌሎች ፍራፍሬዎች |  |  |  |
| 125 | የኤኤንሲ አቅራቢ ለደንበኛው የተለያዩ የምግብ ቡድኖችን የመመገብን አስፈላጊነት/ጥቅም ምክር ሰቷል |  |  |  |
| 126 | የኤ.ኤን.ሲ አቅራቢ በየቀኑ ወቅታዊ ፍራፍሬዎችና አትክልቶች እንዲመገቡ ለደንበኛው አሳውቋል |  |  |  |
| 127 | የኤኤንሲ አገልግሎት ሰጪ ለደንበኛው ስለ በቂ ፈሳሽ መውሰድ አሳውቋል (በ*የቀኑ ቢያንስ 8 ብርጭቆዎች ፈሳሽ*) |  |  |  |
| 128 | የኤንኤሲ አገልግሎት ሰጭ ምግብ በሚቀርብብት ጊዜ (ምግብ ካበሰለ በኋላ) የአዮዲን ጨው ስለመጨመር ለደንበኛው አሳውቋል ፡፡ |  |  |  |
| 129 | የኤኤንሲ አገልግሎት አቅራቢ በእርግዝና ጊዜ ምን መወገድ እንዳለበት ለደንበኛው አሳውቋል (ማለትም አልኮሆል ፣ ጥሬ / ያልበሰሉ ምግቦች እና አትክልቶች ፣ |  |  |  |
| 130 | የኤኤንሲ አቅራቢ በእርግዝና ወቅት ምን መገደብ እንዳለበት ለደንበኛው አሳውቋል (ማለትም ካፌይን (ቡና፣ሻይ) ፣ ስብ) |  |  |  |
| 131 | የኤኤንሲ አገልግሎት ሰጪ የተመጣጠነ ምግብ እጥረት በእርግዝና ወቅት በእናቶች ላይ የሚያስከትለውን ችግሮች ለደንበኛው አሳውቋል |  |  |  |
| 132 | የኤኤንሲ አገልግሎት ሰጪ የተመጣጠነ ምግብ እጥረት በእርግዝና ወቅት በፅንስ ላይ የሚያስከትለውን ችግሮች ለደንበኛው አሳውቋል |  |  |  |
| 133 | የኤንኤንሲ አቅራቢ ልጅ በተወለደ በአንድ ሰአት ውስጥ ጡት ማጥባት መጀመር አስፈላጊነት ላይ መረጃ ሰቷል (አራተኛ ጉብኝት) |  |  |  |
| 134 | የኤኤንሲ አገልግሎት ሰጭ ኮልስትረም/እንገር የመመገብ አስፈላጊነት ላይ መክሯታል (አራተኛ ጉብኝት) |  |  |  |
| 135 | የቅድመ-ጡት ምግብን በማስወገድ ላይ ምክር ተሰጥቷል (አራተኛ ጉብኝት) |  |  |  |
| 136 | ልጇን በቀጥታ ለፀሐይ ብርሃን እንድታሞቀው ምክር ተሰጥቷል (አራተኛ ጉብኝት) |  |  |  |
| 137 | የኤንኤሲ አገልግሎት ሰጭ ለመጀመሪያው ስድስት ወር የእናት ጡት ብቻ መመገብ አስፈላጊነት ላይ መረጃ ሰቷል (አራተኛ ጉብኝት) |  |  |  |
| 138 | እናትየዋ የታከመ ውሃ ለመጠጥ እንድትጠጣ ተመክራለች (ማለትም ከውሀ አጋር ፣ ወይም የፈላ እና የማጣሪያ ውሀ ..) |  |  |  |
| 139 | ከመፀዳጃ ቤት በኋላ እና ከምግብ ዝግጅት በፊት እጇን እንድትታጠብ ተመክራለች |  |  |  |
| 140 | የመመገቢያ ዕቃዎች እና የማብሰያ ቁሳቁሶች በንፅህና እንዲጠብቁ ተመክራለች |  |  |  |
| 141 | እናትየዋ የግል ንፅህናን እንድትጠብቅ ተመክራለች (ማለትም ገላ መታጠብ ..) |  |  |  |
| 142 | በፀረ-ተባይ መድሃኒት የታከመ የአልጋ-አጎበር አጠቃቀም ላይ ምክር ተሰቷታል |  |  |  |
| 143 | ደንበኛውን በሚመክሩበት ጊዜ የሚገኙትን የ የማስተማሪያ እና የተግባቦት መሰሪያዎችን (IEC) እና የምክር መመሪያዎችን ተጠቅሟል |  |  |  |
| 144 | በምክክሩ ማብቂያ ላይ እናትየው በራሪ ወረቀቶች ወይም  ብሮሹሮች ተሰታለች |  |  |  |
|  | **ስለ አስፈላጊ የምክር ችሎታ ምልከታዎች** | | |  |
| 145 | አገልግሎት ሰጪው ደንበኛው የሚናገረውን ያዳምጣል |  |  |  |
| 146 | አገልግሎት ሰጪው ፍላጎትን የሚያሳዩ ምላሾችን እና ምልክቶችን ተጠቅሟል |  |  |  |
| 147 | አገልግሎት ሰጭው መረጃውን ከማስተካከሉ (ከማረሙ) በፊት ደንበኛዋ ያሳሰባት ነገር እንድትናገር የፈቅድላታል |  |  |  |
| 148 | አገልግሎት ሰጪው የመፍረድ ቃላትን ከመጠቀም ተቆጥቧል |  |  |  |
| 149 | አገልግሎት ሰጭው ደንበኛው በትክክል የሚሰራውን ነገር ለይቶ አውቆ አመስግኗል |  |  |  |
| 150 | የአገልግሎት አቅራቢ ቁልፍ ችግሮችን (ካለ) ለይቶ ከደንበኛው ጋር አብሮ ለመስራት በጣም አስፈላጊ የሆነውን ችግር መርጧል |  |  |  |
| 151 | አገልግሎት ሰጪው ደንበኛው ባነሳቸው አማራጮች እና ቁልፍ ችግሮች ላይ ተወያይቷል |  |  |  |
| 152 | የአገልግሎት አቅራቢ በደንበኛው ሁኔታ እና ግብዓት ላይ በመመርኮዝ በጣም ጥሩውን አማራጭ እንዲመርጥ ለመርዳት የሚችሉ እርምጃዎችን ይመክራል እንዲሁም ይደራደራል |  |  |  |
| 153 | አገልግሎት ሰጪው ደንበኛው ከአማራጮቹ ውስጥ አንዱን ለመሞከር እንዲስማማ ረድቷታል እንዲሁም የተስማሙትን እርምጃ እንዲደግሙላቸው ጠይቋል |  |  |  |
| 154 | የአገልግሎት አቅራቢው የደንበኛውን ታሪክ መዝግቧል |  |  |  |
| 155 | ለቀጣይ ክትትል ጉብኝት ቀጠሮ አሲዟል |  |  |  |

**ምልከታ ዝርዝር 6: በድህረ ወሊድ ወቅት ለሚሰጥ አገልግሎት ምልከታ ዝርዝር.**

ቀን: የታዛቢ ስም:

ወረዳ: የጤና ተቋም ስም:

የአገልግሎት ሰጪው ዕድሜ: _________________

የአገልግሎት ሰጪው ጾታ: _________________

የአገልግሎት ሰጪው የሙያ ደረጃ: _________________

የአገልግሎት ሰጪው ስልጠና ውስዷል (አዎ/አይ)

**ክፍል አንድ፥ የድህረ ወሊድ አገልግሎት መስጫ የመሣሪያዎች እና ቁሳቁሶች ምልከታ**

| **[በተገቢው ቦታ ላይ ምልክት ያድርጉ]** | **አዎ** | | **አይ** | **ማስታወሻ** |
| --- | --- | --- | --- | --- |
|  | **ይሰራል** | **አይሰራም** |  |  |
| **የአገልግሎት መስጫ መሣሪያዎች ምልከታ** |  |  |  |  |
| የአዋቂ የክብደት ሚዛን |  |  |  |  |
| MUAC ቴፕ |  |  |  |  |
| የቁመት የመለኪያ |  |  |  |  |
| የህፃናት የክብደት ሚዛን |  |  |  |  |
| የጭንቅላት ዙሪያ መለኪያ ቴፕ |  |  |  |  |
| **Observation on registration, guide lines, manuals, SBCC materials** |  |  |  |  |
| የድህረ ወሊድ ክትትል መዝገብ |  |  |  |  |
| የድህረ ወሊድ ክትትል መመሪያ ማንዋል |  |  |  |  |
| የቤተሰብ እቅድ መመሪያ |  |  |  |  |
| የእናቶች ፣ አራስ ልጆች እና የህፃናት ጤና አጠባበቅ መመሪያ / BEMONC/ |  |  |  |  |
| የአፍላ ወጣት የእናቶች እና የልጆች አመጋገብ መመሪያ |  |  |  |  |
| የብሔራዊ የኤች.አይ.ቪ / ኤድስ አመጋገብ መመሪያ |  |  |  |  |
| የብሔራዊ የማይክሮ ንጥረ-ነገር መመሪያ |  |  |  |  |
| የ IYCF መመሪያ |  |  |  |  |
| ፍላየር ፣ ብሮሹሮች ፣ የምክር ካርድ ፣ ፖስተር |  |  |  |  |

**ክፍል ሁለት፥ በድህረ ወሊድ አገልግሎት ወቅት የአገልግሎት አቅርቦት እና የምክር አገልግሎት ምልከታ**

|  | **ለእናት የተሰጠ የምክር አገልግሎት ምልከታ** |  |  |  |
| --- | --- | --- | --- | --- |
| 101 | ደንበኛው በጡት ማጥባት ጊዜ ሁለት ተጨማሪ ምግብ መውሰድ እንዳለባት እና በቂ እረፍት ማድረግ እንዳለባት ተመክራለች |  |  |  |
| 102 | እናት በፕሮቲን እና በፈሳሽ የበለፀገ ምግብ እንድትመገብ ምክር ተሰታለች |  |  |  |
| 103 | እናት በብረት መአድን የበለፀገ ምግብ እንድትመገብ ተመክራልች |  |  |  |
| 104 | እናት ምንም አይነት በአካባቢ ሊኖሩ የሚችሉ የአመጋገብ ገደቦችን እንድታስወግድ ተመክራለች |  |  |  |
| 105 | ለመጀመሪያው ስድስት ወር ጡት ብቻ እንድታጠባ ተማክራለች |  |  |  |
| 106 | የድህረ ወሊድ አገልግሎት ሰጪ ለመጀመሪያው ስድስት ወር ጡት ብቻ ማጥባት አስፈላጊነት/ጥቅም ላይ መረጃ ሰቷል |  |  |  |
| 107 | ሕፃኑ በፈለገ ግዜ ጡት እንድታጠባ ተመክራለች |  |  |  |
| 108 | ጡት በማጥባት ወቅት ስለ ትክክለኛው አቀማመጥ እና አያያዝ ላይ ተመክራለች |  |  |  |
| 109 | የድህረ ወሊድ አገልግሎት አቅራቢ ደንበኛው በአካባቢ የሚገኘውን ምግብ በመጠቀም ከተለያዩ የምግብ ቡድኖች እንድትመገብ መክሯል (እህሎች ፣ ነጭ ሥሮች, ባቄላ ፣ አተር እና ምስር, ለውዝ ፣ የወተት ተዋጽኦ ፣ ሥጋ ፣ የዶሮ እርባታ እና ዓሳ. እንቁላል ፣ ጥቁር አረንጓዴ ቅጠላማ አትክልቶች ፣ ሌሎች በቫይታሚን ኤ የበለፀጉ ፍራፍሬዎች እና አትክልቶች ፣ ሌሎች አትክልቶች ፣ ሌሎች ፍራፍሬዎች |  |  |  |
| 110 | የድህረ ወሊድ አገልግሎት አቅራቢ ለደንበኛው የተለያዩ የምግብ ቡድኖችን የመመገብን አስፈላጊነት/ጥቅም ምክር ሰቷል |  |  |  |
| 111 | የድህረ ወሊድ አገልግሎት አቅራቢ በየቀኑ ወቅታዊ ፍራፍሬዎችና አትክልቶች እንዲመገቡ ለደንበኛው አሳውቋል |  |  |  |
| 112 | የድህረ ወሊድ አገልግሎት ሰጪ ለደንበኛው ስለ በቂ ፈሳሽ መውሰድ አሳውቋል (በ*የቀኑ ቢያንስ 8 ብርጭቆዎች ፈሳሽ*) |  |  |  |
| 113 | የድህረ ወሊድ አገልግሎት ሰጭ ምግብ በሚቀርብብት ጊዜ (ምግብ ካበሰለ በኋላ) የአዮዲን ጨው ስለመጨመር ለደንበኛው አሳውቋል ፡፡ |  |  |  |
| 114 | የድህረ ወሊድ አገልግሎት ሰጪ በጡት ማጥባት ጊዜ የሚፈጠር የተመጣጠነ የምግብ እጥረት የሚያስከትለውን ችግሮች ለደንበኛው አሳውቋል |  |  |  |
| 115 | እናትየዋ የታከመ ውሃ ለመጠጥ እንድትጠጣ ተመክራለች (ማለትም ከውሀ አጋር ፣ ወይም የፈላ እና የማጣሪያ ውሀ ..) |  |  |  |
| 116 | ተላላፊ በሽታ ሊያስከትሉ የሚችሉ የንፅህና መጠበቂያ (pads) ወይም ሌሎች ቁሳቁሶችን ደህንነቱ በተጠበቀ ሁኔታ ስለማስወገድ ምክር ተሰቷል |  |  |  |
| 117 | እናትና መፀዳጃ ቤት ከተጠቀመች በኋላ ፣ከምግብ ዝግጅት በፊት ፣ ህፃን ካፀዳች በኋላ ፣ ከመመገብ በፊት ፣ ጡት ከመመገብ በፊት እጇን እንድትታጠብ ተመክራለች |  |  |  |
| 118 | የመመገቢያ ዕቃዎች እና የማብሰያ ቁሳቁሶች በንፅህና እንዲጠብቁ ተመክራለች |  |  |  |
| 119 | እናትየዋ የግል ንፅህናን እንድትጠብቅ ተመክራለች (ማለትም ገላ መታጠብ ..) |  |  |  |
| 120 | በቤተሰብ እቅድ ላይ ተመክራለች |  |  |  |
| 121 | በፀረ-ተባይ መድሃኒት የታከመ የአልጋ-አጎበር አጠቃቀም ላይ ምክር ተሰቷታል |  |  |  |
| 122 | ደንበኛውን በሚመክሩበት ጊዜ የሚገኙትን የ የማስተማሪያ እና የተግባቦት መሰሪያዎችን (IEC) እና የምክር መመሪያዎችን ተጠቅሟል |  |  |  |
| 123 | በምክክሩ ማብቂያ ላይ እናትየው በራሪ ወረቀቶች ወይም  ብሮሹሮች ተሰታለች |  |  |  |
|  | **ስለ አስፈላጊ የምክር ችሎታ ምልከታዎች** | | | |
| 124 | አገልግሎት ሰጪው ደንበኛው የሚናገረውን ያዳምጣል |  |  |  |
| 125 | አገልግሎት ሰጪው ፍላጎትን የሚያሳዩ ምላሾችን እና ምልክቶችን ተጠቅሟል |  |  |  |
| 126 | አገልግሎት ሰጭው መረጃውን ከማስተካከሉ (ከማረሙ) በፊት ደንበኛዋ ያሳሰባት ነገር እንድትናገር የፈቅድላታል |  |  |  |
| 127 | አገልግሎት ሰጪው የመፍረድ ቃላትን ከመጠቀም ተቆጥቧል |  |  |  |
| 128 | አገልግሎት ሰጭው ደንበኛው በትክክል የሚሰራውን ነገር ለይቶ አውቆ አመስግኗል |  |  |  |
| 129 | የአገልግሎት አቅራቢ ቁልፍ ችግሮችን (ካለ) ለይቶ ከደንበኛው ጋር አብሮ ለመስራት በጣም አስፈላጊ የሆነውን ችግር መርጧል |  |  |  |
| 130 | አገልግሎት ሰጪው ደንበኛው ባነሳቸው አማራጮች እና ቁልፍ ችግሮች ላይ ተወያይቷል |  |  |  |
| 131 | የአገልግሎት አቅራቢ በደንበኛው ሁኔታ እና ግብዓት ላይ በመመርኮዝ በጣም ጥሩውን አማራጭ እንዲመርጥ ለመርዳት የሚችሉ እርምጃዎችን ይመክራል እንዲሁም ይደራደራል |  |  |  |
| 132 | አገልግሎት ሰጪው ደንበኛው ከአማራጮቹ ውስጥ አንዱን ለመሞከር እንዲስማማ ረድቷታል እንዲሁም የተስማሙትን እርምጃ እንዲደግሙላቸው ጠይቋል |  |  |  |
| 133 | የአገልግሎት አቅራቢው የደንበኛውን ታሪክ መዝግቧል |  |  |  |
| 134 | ለቀጣይ ክትትል ጉብኝት ቀጠሮ አሲዟል |  |  |  |
