# Supplementary material for: Landscape analysis of nutrition services at Primary Health Care Units (PHCUs) in four districts of Ethiopia
Source: PLoS One. 2020 Dec 3;15(12):e0243240. doi: 10.1371/journal.pone.0243240 (PMC7714176; doi:10.1371/journal.pone.0243240)
Supplement: S1 File — (DOCX) [file pone.0243240.s003.docx]

**Key informant interview guide for Woreda officials**

**Discussion goal:**

The goal of this discussion is to assess the overall implementation of comprehensive and integrated nutrition services in the Woreda. This assessment will serve as a rapid situational analysis to understand the implementation process, to identify the gaps and success which will serve as a stepping stone to further improve service provision.

**Facilitator/ Note-Taker Instructions**

- Modify/adapt questions as appropriate to the interviewee.
- For each question, facilitators should probe as indicated and appropriate. Please note that the questions are intended to serve as a guide, not a script.
- Keep within the allotted time.
- Take detailed notes on responses, focusing on key points, using the department template.

**Background script**

Hello my name is …………. I am member of the 1000 plus day project for scale up nutritional project create a model Woreda.

As was mentioned, we are doing a project to create a model woredas, nutritional interventions We are conducting group discussion with you to identify some of the challenges you are facing in delivering comprehensive and integrated nutritional services at the Woreda level so that we can come up with a better delivering strategy?

I want you to know there are no right or wrong answers during our discussion. We want to know your opinion.  Please feel free to share your opinions, both positive and negative. We greatly appreciate your honesty and openness with us so we can paint as accurate a picture as possible for the assessment. Our discussion will last about 45 minutes.  What we learn through these discussions will help the project in the nutritional service assessment and planning process at the woreda level. key themes will be incorporated into the rapid assessment report. No names or organizations will be connected to anything that any  person said in the discussion.  Any quotes we put in the report will be presented anonymously.  Additionally, nothing sensitive that can be connected to any organization or individual will be discussed in the report. However, at the end for the report we will describe the organizations that contributed in this assessment.

Do you mind if we record audio of our session today so we can make sure our notes are accurate? We will erase the recording once we complete our notes. This is optional, so if  this makes you feel uncomfortable, we will just take handwritten notes.

Do you have any questions before we begin? Before we begin, if you haven’t already, please turn off your cell phones or put them on vibrate mode.  Again, thank you for being here and sharing feedback with us. Let’s get started.

**Introductions**

Let’s start by getting to know one another

**Discussion**

1. How do you explain the overall nutrition service in the Woreda?

- *probe: CINS*

1. How do you describe the multi-sectoral collaboration regarding nutritional services and programs?

- *Existence and functionality of WNCB/WNTC*
- *Who are the member of the coordination body?*
- *What are the sectors involved? Why not others?*
- *What is the role and responsibility of each sectors involved in the coordination body?*
- *How frequent and regular is the meeting?*
- *Which sectors are actively engaged? Why not others?*

1. How do you asses the planning and monitoring of nutrition service?

- *Is the planning participatory, (annual, quarter plan)*
- *What is the baseline?*
- *What are the indicators?*
- *How do you evaluate the activities done by the coordination body/technical committee?*

1. How is the supportive supervision on nutritional programs?

- *Direction of supervision*
- *Frequency of supervision (regularity)*
- *How do you document (do they have action plan based on supervision finding?)*
- *Follow up (checking for improvement from previous feedback)*
- *What are the supports*
- *Gaps identified*
- *Feedback from the supervising body?*

1. What are challenges and success have you encountered regarding human resource

- *Ratio, staff turnover,*
- *Motivation, capacity gap, training needs, etc.*

1. Can you please explain the logistic management related with nutrition service?

- *IFA, VAS, Albendazole, tablet or supplement, antibiotics, ITN, tally sheets, register, guidelines/manuals, reporting formats, SBCC materials, rooms (GMP, SC, mothers Waiting, etc.) nutritional screening apparatus)*
- *How frequent is the stock out?*
- *Which item is commonly stocked out? why?*
- *What is the main reason for stock out?*
- *How do they manage to prevent stock out?*
- *How do they cope when there is stock out?*

1. How do you integrate nutrition interventions with other programs and what challenges exists?

- *Probe: what could be done to improve integration among different stakeholders*

1. How is the registration and reporting process

- *Probe: who is registering,*
- *Completeness, legibility, timeliness*
- *Challenge*

1. Challenges, best practice and recommendation?

CLOSING: Thank you so much for your time and for sharing your opinion. We really value your feedback and help in making the rapid assessment successful. Before we end the discussion is there anything that you wanted to add that you did not get the chance to bring up earlier? On behalf of Addis Ababa university school of public health coordinating team, I want to thank you once again for your time

**Key informant interview guide for health professionals**

**Discussion goal:**

The goal of this discussion is to assess the overall comprehensive and integrated nutritional health services which will serve as a situational assessment and to understand the overall nutritional programs and services undergoing in this health facilitiy.

**Facilitator/ Note-Taker Instructions**

- Modify/adapt questions as appropriate to the interviewee.
- For each question, facilitators should probe as indicated and appropriate. Please note that the questions are intended to serve as a guide, not a script.
- Keep within the allotted time.
- Take detailed notes on responses, focusing on key points, using the department template.

**Background script**

Hello my name is …………. I am member of the 1000 plus day project for scale up nutritional project create a model Woreda.

As was mentioned, we are doing a project to create a model woredas, nutritional interventions

We are conducting this interview with you to identify some of the challenges you are facing in delivering comprehensive and integrated nutritional services at the Woreda level so that we can come up with a better delivering strategy?

I want you to know there are no right or wrong answers during our discussion. We want to know your opinion.  Please feel free to share your opinions, both positive and negative. We greatly appreciate your honesty and openness with us so we can paint as accurate a picture as possible for the assessment.

Our discussion will last about 45 minutes. What we learn through theses discussions will help the project in nutritional service assessment and planning process at the woreda level. Key themes will be incorporated into the rapid assessment report. No names or organizations will be connected to anything that any person said in the discussion. Any quotes we put in the report will be presented anonymously. Additionally, nothing sensitive that can be connected to any organization or individual will be discussed in the report. However, at the end of the report we will describe the organization that contributed in this assessment.

Do you mind if we record audio of our session today so we can make sure our notes are accurate? We will erase the recording once we complete our notes. This is optional, so if  this makes you feel uncomfortable, we will just take handwritten notes.

Do you have any questions before we begin? Before we begin, if you haven’t already, please turn off your cell phone or put them on vibrate mode.  Again, thank you for being here and sharing feedback with us. Let’s get started.

**Introductions**

Let’s start by getting to know one another

**Discussion points**

1. How do you describe the nutritional services in this health facility? (*probe:*

- *Detail description of the service given and the process(quality) in different contact point e.g. ANC, GMP, immunization..)*

1. How do you see the integration of the different nutrition services that you have mentioned above in your health facility?

- *How they managed to avoid missed opportunity, e.g. has every child anthropometric measurement has been taken and linked with appropriate service..)*
- *Is there a platform that gather nutrition service provider in your health facility?*

1. How do you explain the referral modalities that exist in your health facility?

- *Referral to hospital or health center*

1. How do you perceive the man power that provide nutrition service in your health facility?

- *Adequacy of staff, trained staff, capacity of the provider, motivation*
- *In and pre service training opportunity (frequency, who are selected, duration of training)*
- *Rotation of staff*
- *Case load as compared to the number of man power*

1. How do you describe the multi-sectoral collaboration regarding nutritional services and programs?

- *What are the coordination points (school, agriculture..)*
- *Who are the participants? Why not others?*
- *What is the role of your health facility/ yours?*
- *How frequent do you conduct review meetings?*

1. How is the supportive supervision on nutritional programs?

- *Direction of supervision*
- *Frequency of supervision (regularity)*
- *How do you document (action plan based on supervision finding)*
- *Follow up (checking for improvement from previous feedback)*
- *What are the supports?*
- *Gaps identified*
- *Feedback from the supervising body?*

1. How do you asses the planning and monitoring of nutrition service?

- *Is the planning participatory, (annual, quarter plan)*
- *What is the baseline*
- *What are the indicators*

1. How is the registration and reporting process?

- *who is registering, completeness, legibility, timeliness, challenge)?*

1. Can you please explain the logistic management related with nutrition service?

- *Tablets, or supplements, antibiotics, nutritional screening apparatus*
- *Use of bin card, internal audit*
- *How frequent is the stock out?*
- *Which item is common stocked out?*
- *What is the main reason for stock out?*
- *How do they manage to prevent stock out?*
- *How do they cope when there is stock out?*

1. Before closing would like to share challenges, best practice and recommendations that you have for us?

CLOSING: Thank you so much for your time and for sharing your opinion. We really value your feedback and help in making the rapid assessment successful. Before we end the discussion is there anything that you wanted to add that you did not get the chance to bring up earlier? On behalf of Addis Ababa university school of public health coordinating team, I want to thank you once again for your time.

**ከወረዳ ባለሙያ ቁልፍ መረጃ ሰጭ ጋር ለሚደረግ ቃለ መጠይቅ መመሪያ**

**የቃለመጠይቁ ግብ:**

የዚህ ውይይት ግብ አጠቃላይ የተቀናጀ የስነ ምግብ እና የጤና አገልግሎቶችን በመገምገም እና በዚህ ወረዳ የሚከናወኑትን አጠቃላይ የስነምግብ መርሃግብሮች እና የጤና አገልግሎቶችን በመገንዘብ ለሁለንተናዊ ምዘና የሚያገለግሉ መረጃዎችን ለመሰብሰብ ነው።.

**ቃለ መጠይቅ አድራጊ/ማስታወሻ ወሳጅ የሚረዳ መመሪያ**

- ጥያቄዎችን በተገቢው መንግድ ማስተካከል ወይም መቀየር ይቻላል
- ለእያንዳንዱ ጥያቄ ቃለ መጠይቅ አድራጊው በተጠቀሰው እና በተገቢው ሁኔታ በጥልቅ መመርመር አለባቸው. ጥያቄዎቹ እስክሪፕት/የማይቀየሩ ሳይሆን እንደ መመሪያ ለማገልገል የታሰቡ መሆናቸውን እባክዎ ልብ ይበሉ ፡፡
- በተጠቀሰው ጊዜ ውስጥ ይቆዩ
- የመምሪያውን አብነት በመጠቀም በቁልፍ ነጥቦች ላይ በማተኮር በምላሾች ላይ ዝርዝር ማስታወሻዎችን ይያዙ.

**የጀርባ ጽሑፍ**

ጤና ይስጥልኝ ስሜ …………. ይባላል። ሞዴል ወረዳን ለመፍጠር የሚሰራው የ 1000 ቀን (1000 days plus) ፕሮጀክት አባል ነኝ።.

እንደተጠቀሰው በስነ ምግብ ዙሪያ ሞዴል ወረዳዎችን ለመፍጠር ፕሮጀክት እየሰራን ነው

ይህንን ቃለ መጠይቅ ከእርስዎ ጋር እያደረግን ያለው ሁሉን አቀፍ እና የተቀናጀ የስነምግብ አገልግሎቶችን በማቅረብ ረገድ የሚያጋጥሙዎትን ተግዳሮቶች የተወሰኑትን ለመለየት እና የተሻለ የማዳበሪያ ስትራቴጂ ይዘን እንድንመጣ ነው፡፡

በውይይታችን ወቅት ትክክለኛ ወይም የተሳሳቱ መልሶች እንደሌሉ እንድታውቁ እፈልጋለሁ ፡፡ የእርስዎን አስተያየት ማወቅ እንፈልጋለን ፡፡ አስተያየቶችዎን አዎንታዊም ይሁን አፍራሽ ለማጋራት ነፃነት ይሰማዎ ፡፡ ለግምገማው በተቻለ መጠን ትክክለኛውን ስዕል መሳል እንድንችል ከእኛ ጋር ያለውን ቅንነት እና ግልጽነት በጣም እናደንቃለን ፡፡

ለ 45 ደቂቃ ያህል ይቆያል ፡፡ በእነዚህ ውይይቶች የምንማረው ነገር በወረዳው ደረጃ በስነምግብ አገልግሎት ምዘና እና እቅድ ሂደት ውስጥ እንዲሁም ለፕሮጀክቱ ግብአትነት ይረዳል ቁልፍ ጭብጦች በፈጣን ግምገማ ሪፖርቱ ውስጥ ይካተታሉ በውይይቱ ውስጥ ማንም ሰው ከተናገረው ማንኛውም ነገር ጋር ስሞች ወይም ድርጅቶች አይገናኙም።  በሪፖርቱ ውስጥ የምናስቀምጣቸው ማናቸውም ጥቅሶች ማንነትን የሚገልፅ ነገር አይኖራቸውም።  በተጨማሪም በሪፖርቱ ውስጥ ከማንኛውም ድርጅት ወይም ግለሰብ ጋር ሊገናኝ እና ችግር ሊፈጥር የሚችል ማንኛውም ጉዳይ አይገለፅም። ሆኖም በሪፖርቱ መጨረሻ በዚህ ግምገማ ውስጥ አስተዋፅዖ ያደረጉትን ድርጅቶች እንገልፃለን ፡፡

ማስታወሻዎቻችን ትክክለኛ መሆናቸውን ማረጋገጥ እንድንችል የውይይታችንን ድምፅ ብንቀዳ ቅር ይልዎታል? ማስታወሻዎቻችንን እንደጨረስን ቀረጻውን እናጠፋለን ፡፡ ይህ እንደ አማራጭ ነው ፣ ስለሆነም ይህ ምቾት የማይሰማዎት ከሆነ በእጅ የተጻፉ ማስታወሻዎችን ብቻ እንወስዳለን።

ከመጀመራችን በፊት ጥያቄዎች አሉዎት? ከመጀመራችን በፊት እስካሁን ካላደረጉ እባክዎን ሞባይል ስልካችሁን ያጥፉ ወይም በንዝረት ሞድ ላይ ያኑሯቸው ፡፡ እንደገና ፣ እዚህ ስለነበሩ እና ከእኛ ጋር ግብረመልስ ስላጋሩ እናመሰግናለን ፡፡ እንጀምር.

**መግቢያዎች**

እርስ በእርስ በመተዋወቅ እንጀምር

**የውይይት ነጥቦች**

1. በዚህ ወረዳ ውስጥ ያሉ የስነ-ምግብ አገልግሎቶችን እንዴት ይገልፁታል?

- (ምርመራ: ስለ ተቀናጀ የስነ-ምግብ አገልግሎት (CINS) ይመርምሩ

1. የስነ-ምግብ አገልግሎቶችን እና ፕሮግራሞችን በተመለከተ የብዙ ዘርፎች ትብብር/ቅንጅትን/ (multi-sectoral collaboration) እንዴት ይመለከታሉ?

- *የወረዳ ኮኦርድኔሽን ቦዲ እና ቴክኒካል ኮሚቴ (WNCB/WNTC) መኖር እና ተግባራዊነት (ስራ ላይ መዋል)*
- *የዚህ አካል አባል የሆኑት እነማን ናቸው?*
- *በዚህ አካል ላይ ተሳታፊዎቹ እነማን ናቸው? ሌሎች ለምን አይሳተፉም?*
- *በዚህ አካል ውስጥ የተሳተፉት እያንዳንዱ ዘርፎች/ ሴክተሮች ሚና እና ኃላፊነት ምንድነው?*
- *ስብሰባዎች በምን ያህል ጊዜ ይካሄዳሉ እና ምን ያህል መደበኛ ነው?*
- *የትኞቹ ዘርፎች/ሴክተሮች በንቃት ተሰማርተዋል? ሌሎች ለምን አልተሰማሩም ?*

1. የስነ-ምግብ አገልግሎቶችን እቅድ እና ቁጥጥር እንዴት ይረዱዎታል/ይመለከቱታል?

- *ዕቅዱ አሳታፊ ነው?(አመታዊ፣የሩብ አመት እቅድ)*
- *ለእቅዱ መነሻው ምንድ ነው (baseline)*
- *አመላካቾች (indicators) እነማን ናቸው?*
- *በወረዳ ኮኦርድኔሽን ቦዲ / ቴክኒካል ኮሚቴ የተከናወኑ ተግባራት እንዴት ይገመግማሉ?*

1. በስነ-ምግብ መርሃግብሮች ላይ ያለ የድጋፍ ቁጥጥር እንዴት ነው?

- የድጋፍ ቁጥጥር አቅጣጫ እንዴት ነው
- *የክትትል ድግግሞሽ (መደበኛነት)*
- *እንዴት ይመዘገባሉ (በክትትል ግኝት ላይ የተመሠረተ እቅድ ማቀድ)*
- *የመከታተል ነገርስ እንዴት ነው(ከቀዳሚው ግብረመልስ የተሻሻል ነገር ስለመኖሩ የመፈተሽ ስራስ)*
- *ምን አይነት ድጋፎቹ ይሰጣሉ*
- *ምን አይነት ክፍተቶች ተለይተዋል*
- *ከተቆጣጣሪው አካል ምን አይነት ግብረመልስ ተሰቷል*

1. የሰው ኃይልን በተመለከተ ምን ችግሮች እና ስኬት አጋጥመውዎታል?

- *የአገልግሎት ተጠቃሚው ቁጥር ከሰው ኃይል ቁጥር ጋር ሲነፃፀር*
- *የሰራተኛ ከስራ የመልቀቅ ነገር*
- *የሥልጠና ዕድል ፣ የሰራተኛ ተነሳሽነት፣ የአገልገሎት ሰጪው አቅም/ብቃት*

1. ከስነ-ምግብ አገልግሎት ጋር የተያያዘ የግብአት አያያዝን ማስረዳት ይችላሉ?

- *አይ.ኤፍ.ኤ(IFA), ቫይታሚን ኤ, አልቤንዳዞል ፣ ታብሌት፣ አንቲባዮቲክስ, አጎበር, መዝገቦች, መመሪያ ማኑዋሎች, የሪፖርት ቅፆች, የምክር መስጫ መሳሪያዎች፣ የተለያዩ የቁመት፣ ክብደት እንዲሁም MUAC መለኪያ መሳሪያዎች*
- *የግብአት እጥረት በምን ያህል ፍጥነት/ድግግሞሽ ያጋጥማል?*
- *የትኛው ግብአት በይበልጥ ብዙ ግዜ እጥረት ይገጥማል? ለምን?*
- *የግብአት እጥረት ለመፈጠሩ ዋነኛው ምክንያት ምንድን ነው?*
- *የግብአት እጥረት እንዳይፈጠር ምን አይነት ስራዎች ይሰራሉ?*
- *የግብአት እጥረት ሲገጥም ሰራዎች ሳይቋረጡ እንዲሰሩ ምን ነገሮች ይደረጋሉ?*

1. የስነ-ምግብ መርሃግብሮችን/ፕሮግራሞች ከሌሎች ፕሮግራሞች ጋር እንዴት ያዋህዳሉ/እንዴት በተቀናጅ ሁኔታ እንዲሰራ ይደረጋሉ? ምን አይነት ችግሮችስ አሉ?

- *መርምር: በተለያዩ ባለድርሻ አካላት መካከል ውህደትን/ቅንጅትን ለማሻሻል ምን ሊደረግ ይችላል*

1. የምዝገባ እና ሪፖርት አሰራር ሂደት እንዴት ነው??

- *ማነው ምዝገባውን የሚያካሂደው፣ ምሉነት ፣ ተነባቢነት ፣ ወቅታዊነት*
- *ከዚህ ጋር በተያያዘ ያሉ ተግዳሮቶች*

1. በስተመጨረሻ ምን አይነት ተግዳሮቶች፣ ምርጥ ልምምዶች እና ምክሮችን ሊያካፍሉን የወዳሉ?

መዝጊያ: ስለ ጊዜዎ እና አስተያየትዎን ስላካፈሉን በጣም አመሰግናለሁ። ፈጣን ምዘናውን ስኬታማ ለማድረግ ስለሰጡን ምላሽ በእውነት ከፍ አድርገን እናመሰግኖታለን። ውይይቱን ከማጠናቀቃችን በፊት ቀደም ብለው የማቅረብ እድሉን ያላገኙበት ለመጨመር የሚፈለጉት ሀሳብ አለ? በአዲስ አበባ ዩኒቨርስቲ የህብረተሰብ ጤና ትምህርት ክፍል እንዲሁም በፕሮጀክቱ አስተባባሪ ቡድን ስም ፣ ስለ ጊዜዎ በድጋሚ ላመሰግናችሁ እፈልጋለሁ ፡፡

**ከጤና ባለሙያ ቁልፍ መረጃ ሰጭ ጋር ለሚደረግ ቃለ መጠይቅ መመሪያ**

**የቃለመጠይቁ ግብ:**

የዚህ ውይይት ግብ አጠቃላይ የተቀናጀ የስነ ምግብ እና የጤና አገልግሎቶችን በመገምገም እና በዚህ የጤና ተቋማት ውስጥ የሚከናወኑትን አጠቃላይ የስነምግብ መርሃግብሮች እና የጤና አገልግሎቶችን በመገንዘብ ለሁለንተናዊ ምዘና የሚያገለግሉ መረጃዎችን ለመሰብሰብ ነው ፡፡

**ቃለ መጠይቅ አድራጊ/ማስታወሻ ወሳጅ የሚረዳ መመሪያ**

- ጥያቄዎችን በተገቢው መንግድ ማስተካከል ወይም መቀየር ይቻላል
- ለእያንዳንዱ ጥያቄ ቃለ መጠይቅ አድራጊው በተጠቀሰው እና በተገቢው ሁኔታ በጥልቅ መመርመር አለባቸው. ጥያቄዎቹ እስክሪፕት/የማይቀየሩ ሳይሆን እንደ መመሪያ ለማገልገል የታሰቡ መሆናቸውን እባክዎ ልብ ይበሉ ፡፡
- በተጠቀሰው ጊዜ ውስጥ ይቆዩ
- የመምሪያውን አብነት በመጠቀም በቁልፍ ነጥቦች ላይ በማተኮር በምላሾች ላይ ዝርዝር ማስታወሻዎችን ይያዙ.

**የጀርባ ጽሑፍ**

ጤና ይስጥልኝ ስሜ …………. ይባላል። ሞዴል ወረዳን ለመፍጠር የሚሰራው የ 1000 ቀን (1000 days plus) ፕሮጀክት አባል ነኝ።.

እንደተጠቀሰው በስነ ምግብ ዙሪያ ሞዴል ወረዳዎችን ለመፍጠር ፕሮጀክት እየሰራን ነው

ይህንን ቃለ መጠይቅ ከእርስዎ ጋር እያደረግን ያለው ሁሉን አቀፍ እና የተቀናጀ የስነምግብ አገልግሎቶችን በማቅረብ ረገድ የሚያጋጥሙዎትን ተግዳሮቶች የተወሰኑትን ለመለየት እና የተሻለ የማዳበሪያ ስትራቴጂ ይዘን እንድንመጣ ነው፡፡

በውይይታችን ወቅት ትክክለኛ ወይም የተሳሳቱ መልሶች እንደሌሉ እንድታውቁ እፈልጋለሁ ፡፡ የእርስዎን አስተያየት ማወቅ እንፈልጋለን ፡፡ አስተያየቶችዎን አዎንታዊም ይሁን አፍራሽ ለማጋራት ነፃነት ይሰማዎ ፡፡ ለግምገማው በተቻለ መጠን ትክክለኛውን ስዕል መሳል እንድንችል ከእኛ ጋር ያለውን ቅንነት እና ግልጽነት በጣም እናደንቃለን ፡፡

ውይይታችን ለ 45 ደቂቃ ያህል ይቆያል ፡፡ በእነዚህ ውይይቶች የምንማረው ነገር በወረዳው ደረጃ በስነምግብ አገልግሎት ምዘና እና እቅድ ሂደት ውስጥ እንዲሁም ለፕሮጀክቱ ግብአትነት ይረዳል ቁልፍ ጭብጦች በፈጣን ግምገማ ሪፖርቱ ውስጥ ይካተታሉ በውይይቱ ውስጥ ማንም ሰው ከተናገረው ማንኛውም ነገር ጋር ስሞች ወይም ድርጅቶች አይገናኙም።  በሪፖርቱ ውስጥ የምናስቀምጣቸው ማናቸውም ጥቅሶች ማንነትን የሚገልፅ ነገር አይኖራቸውም።  በተጨማሪም በሪፖርቱ ውስጥ ከማንኛውም ድርጅት ወይም ግለሰብ ጋር ሊገናኝ እና ችግር ሊፈጥር የሚችል ማንኛውም ጉዳይ አይገለፅም። ሆኖም በሪፖርቱ መጨረሻ በዚህ ግምገማ ውስጥ አስተዋፅዖ ያደረጉትን ድርጅቶች እንገልፃለን ፡፡

ማስታወሻዎቻችን ትክክለኛ መሆናቸውን ማረጋገጥ እንድንችል የውይይታችንን ድምፅ ብንቀዳ ቅር ይልዎታል? ማስታወሻዎቻችንን እንደጨረስን ቀረጻውን እናጠፋለን ፡፡ ይህ እንደ አማራጭ ነው ፣ ስለሆነም ይህ ምቾት የማይሰማዎት ከሆነ በእጅ የተጻፉ ማስታወሻዎችን ብቻ እንወስዳለን።

ከመጀመራችን በፊት ጥያቄዎች አሉዎት? ከመጀመራችን በፊት እስካሁን ካላደረጉ እባክዎን ሞባይል ስልካችሁን ያጥፉ ወይም በንዝረት ሞድ ላይ ያኑሯቸው ፡፡ እንደገና ፣ እዚህ ስለነበሩ እና ከእኛ ጋር ግብረመልስ ስላጋሩ እናመሰግናለን ፡፡ እንጀምር.

**መግቢያዎች**

እርስ በእርስ በመተዋወቅ እንጀምር

**የውይይት ነጥቦች**

1. በዚህ የጤና ተቋም ውስጥ ያሉ የስነ-ምግብ አገልግሎቶችን እንዴት ይገልፁታል? (*ምርመራ:*

- *በተለያየ ክፍል ውስጥ የሚሰጠው አገልግሎት ዝርዝር መግለጫ እና የሂደቱ (ጥራት) ለምሳሌ ቅድመ ወሊድ ክትትል ክፍል፣ የክትባት ክፍል….)*

1. ከላይ የጠቀሷቸውን የተለያዩ የስነ-ምግብ አገልግሎቶችን በጤና ተቋምዎ ውስጥ በተቀናጀ ሁኔታ የመሰጠቱን ነገር እንዴት ያዩታል?

- *ማንም ሰው አገልግሎቱን ሳያገኝ እንዳይሄድ ምን አይነት ነገሮች ይሰራሉ? ለምሳሌ እያንዳንዱ ልጆች የአንትሮፖሜትሪክ ልኬት ተወስዶላቸው ከተገቢው አገልግሎት ጋር ግንኙነት እንዲፈጠር ተሰርቷል...)*
- *በጤና ተቋምዎ ውስጥ የስነ-ምግብ አገልግሎት ሰጪን የሚሰበስብ መድረክ አለ?*

1. በጤና ተቋምዎ ውስጥ ያሉትን የማጣቀሻ ዘዴዎች (referral modalities) እንዴት ያብራራሉ?

- *ወደ ሆስፒታል ወይም ወደ ጤና ጣቢያ መላክ*

1. ጤና ተቋምዎ ውስጥ የስነ- ምግብ አገልግሎት የሚሰጡትን ሰው ኃይል እንዴት ይመለከታሉ?

- *የሰራተኞች ቁጥር ብዛት በቂነት ፣ በቂ የሰለጠኑ ሰራተኞች መኖር ፣ የአገልገሎት ሰጪው አቅም/ብቃት ፣ ተነሳሽነት*
- *በአገልግሎት ውስጥ እያሉ እና የቅድመ አገልግሎት ሥልጠና ዕድል (ድግግሞሽ ፣ የሥልጠና ጊዜ፣ የሚመረጡ ስዎች አይነት)*
- *የሰራተኞች መቀያየር*
- *የአገልግሎት ተጠቃሚው ቁጥር ከሰው ኃይል ቁጥር ጋር ሲነፃፀር*

1. የስነ-ምግብ አገልግሎቶችን እና ፕሮግራሞችን በተመለከተ የብዙ ዘርፎች ትብብር/ቅንጅትን/ (multi-sectoral collaboration) እንዴት ይመለከታሉ?

- *የ*ቅንጅት *ነጥቦቹ ምንድን ናቸው (ትምህርት ቤት ፣ ግብርና ..)*
- *በዚህ አካል ላይ ተሳታፊዎቹ እነማን ናቸው? ሌሎች ለምን አይሳተፉም?*
- *የጤና ተቋምዎ / የእርስዎ ድርሻ ምንድነው?*
- *የግምገማ ስብሰባዎችን በምን ያህል ጊዜ ያካሂዳሉ?*

1. በስነ-ምግብ መርሃግብሮች ላይ ያለ የድጋፍ ቁጥጥር እንዴት ነው?

- የድጋፍ ቁጥጥር አቅጣጫ እንዴት ነው
- *የክትትል ድግግሞሽ (መደበኛነት)*
- *እንዴት ይመዘገባሉ (በክትትል ግኝት ላይ የተመሠረተ እቅድ ማቀድ)*
- *የመከታተል ነገርስ እንዴት ነው(ከቀዳሚው ግብረመልስ የተሻሻል ነገር ስለመኖሩ የመፈተሽ ስራስ)*
- *ምን አይነት ድጋፎቹ ይሰጣሉ*
- *ምን አይነት ክፍተቶች ተለይተዋል*
- *ከተቆጣጣሪው አካል ምን አይነት ግብረመልስ ተሰቷል*

1. የስነ-ምግብ አገልግሎቶችን እቅድ እና ቁጥጥር እንዴት ይረዱዎታል/ይመለከቱታል?

- *ዕቅዱ አሳታፊ ነው?(አመታዊ፣የሩብ አመት እቅድ)*
- *ለእቅዱ መነሻው ምንድ ነው (baseline)*
- *አመላካቾች (indicators) እነማን ናቸው?*

1. የምዝገባ እና ሪፖርት አሰራር ሂደት እንዴት ነው??

- *ማነው ምዝገባውን የሚያካሂደው፣ ምሉነት ፣ ተነባቢነት ፣ ወቅታዊነት*
- *ከዚህ ጋር በተያያዘ ያሉ ተግዳሮቶች*

1. ከስነ-ምግብ አገልግሎት ጋር የተያያዘ የግብአት አያያዝን ማስረዳት ይችላሉ??

- *እንክብሎች፣ አንቲባዮቲኮች፣ ሰፕልመንቶች፣ የተለያዩ የቁመት፣ ክብደት እንዲሁም MUAC መለኪያ መሳሪያዎች*
- *የቢን ካርድ አጠቃቀም ፣ የውስጥ ኦዲት*
- *የግብአት እጥረት በምን ያህል ፍጥነት/ድግግሞሽ ያጋጥማል?*
- *የትኛው ግብአት በይበልጥ ብዙ ግዜ እጥረት ይገጥማል ?ለምን?*
- *የግብአት እጥረት ለመፈጠሩ ዋነኛው ምክንያት ምንድን ነው?*
- *የግብአት እጥረት እንዳይፈጠር ምን አይነት ስራዎች ይሰራሉ?*
- *የግብአት እጥረት ሲገጥም ሰራዎች ሳይቋረጡ እንዲሰሩ ምን ነገሮች ይደረጋሉ?*

1. በስተመጨረሻ ምን አይነት ተግዳሮቶች፣ ምርጥ ልምምዶች እና ምክሮችን ሊያካፍሉን የወዳሉ?

መዝጊያ: ስለ ጊዜዎ እና አስተያየትዎን ስላካፈሉን በጣም አመሰግናለሁ። ፈጣን ምዘናውን ስኬታማ ለማድረግ ስለሰጡን ምላሽ በእውነት ከፍ አድርገን እናመሰግኖታለን። ውይይቱን ከማጠናቀቃችን በፊት ቀደም ብለው የማቅረብ እድሉን ያላገኙበት ለመጨመር የሚፈለጉት ሀሳብ አለ? በአዲስ አበባ ዩኒቨርስቲ የህብረተሰብ ጤና ትምህርት ክፍል እንዲሁም በፕሮጀክቱ አስተባባሪ ቡድን ስም ፣ ስለ ጊዜዎ በድጋሚ ላመሰግናችሁ እፈልጋለሁ ፡፡
