# Supplementary material for: Landscape analysis of nutrition services at Primary Health Care Units (PHCUs) in four districts of Ethiopia
Source: PLoS One. 2020 Dec 3;15(12):e0243240. doi: 10.1371/journal.pone.0243240 (PMC7714176; doi:10.1371/journal.pone.0243240)
Supplement: Attachment — Submitted filename: PONE-D-20-17292_R2_KKS.pdf [file pone.0243240.s006.pdf]

PLOS ONE

Landscape analysis of nutrition services at Primary Health Care Units (PHCUs) in four districts of Ethiopia.

--Manuscript Draft--

**Manuscript Number:**	PONE-D-20-17292R2
**Article Type:**	Research Article
**Full Title:**	Landscape analysis of nutrition services at Primary Health Care Units (PHCUs) in four districts of Ethiopia.
**Short Title:**	Landscape analysis of nutrition services at Primary Health Care Units (PHCUs) in four districts of Ethiopia.
**Corresponding Author:**	Esete Habtemariam Fenta, MPH<br>Addis Ababa University<br>Addis Ababa, ETHIOPIA
**Keywords:**	1000days nutrition, Landscape analysis, Nutrition service evaluation, PHCU, Ethiopia
**Abstract:**	Background: Good nutrition and healthy growth during the first 1000days have lasting benefit throughout life. For this, equally important is the structural readiness of health facilities. However, structural readiness and nutrition services provision during the first 1000 days in Ethiopia is not well understood. The present study was part of a broader implementation research aimed at developing model nutrition districts by implementing evidence based, high impact and cost-effective package of nutrition interventions through the continuum of care. This study was aimed at assessing structural readiness of health facilities and the extent of nutrition service provision in the implementation districts.<br>Methods: This assessment was conducted in four districts of Ethiopia. We used mixed method; a quantitative study followed by qualitative exploration. The quantitative part of the study addressed two-dimensions, structural readiness and process of nutrition service delivery. The first dimension assessed attributes of context in which care is delivered by observing availability of essential logistics. The second dimension assessed the service provision through direct observation of care at different units of health facilities. For these dimensions, we conducted a total of 380 observations in 23 health centers and 33 health posts. The observations were conducted at the Integrated Management of Neonatal and Childhood Illnesses unit, immunization unit, Antenatal care unit and Postnatal care unit. The qualitative part included a total of 60 key informant interviews with key stakeholders and service providers.<br>Result: We assessed structural readiness of 56 health facilities. Both quantitative and qualitative findings revealed poor structural readiness and gap in nutrition services provision. Health facilities lack essential logistics which was found to be more prominent at health posts compared to health centers. The process evaluation showed a critical missed opportunity for anthropometric assessment and preventive nutrition counselling at different contact points. This was particularly prominent at immunization unit (where only 16.4% of children had their weight measured and only 16.2% of mothers with children under six month of age were counselled about exclusive breastfeeding). Although 90.4% of pregnant women who came for antenatal care were prescribed iron and folic acid supplementation, only 57.7% were counselled about the benefit and 42.4% were counselled about the side effect. The qualitative findings showed major service provision bottlenecks including non-functionality of the existing district nutrition coordination body and technical committees, training gaps, staff shortage, high staff turnover resulting in work related burden, fatigue and poor motivation among service providers.<br>Conclusion: We found a considerable poor structural readiness and gaps in delivering integrated nutrition services with a significant missed opportunity in nutrition screening and counselling. Ensuring availability of logistics and improving access to training might improve delivery of nutrition services. In addition, ensuring adequate human resource might reduce missed opportunity and enable providers to provide a thorough preventive counselling service.
**Order of Authors:**	Esete Habtemariam Fenta, MPH
	Bilal Shikur Endris

	Yalemwork Getenet Mengistu
	Fekadu Elias Sadamo
	Endashaw Hailu Gelan
	Tsegaye Gebrezgher Beyene
	Seifu Hagos Gebreyesus
**Opposed Reviewers:**	
**Response to Reviewers:**	November 13, 2020<br>Dear Editor<br>PLOS ONE Journal<br>we would like to thank you for reviewing the manuscript and giving us constructive comments and recommendations. We appreciate the opportunity given to us to revise and resubmit this manuscript. Below are our responses to the comments from the reviewers and a description of what changes we have made (texts in bullet points and italic font).<br><br>Reviewer comments:<br>Reviewer 1:<br>1.Thank you for submitting a revised manuscript titled "Landscape analysis of nutrition services at Primary Health Care Units (PHCUs) in four districts of Ethiopia". I participated as a reviewer for the initial evaluation of this manuscript. I have reviewed the revised manuscript against the reviewer's comments on the earlier version. In my view, all reviewer's comments have been addressed properly in the current version, thank you. However, the manuscript, in my view, will be benefitted if you could address the minor editorial issues throughout the manuscript. Below are a few examples but it would be good if a thorough editing can be done throughout the manuscript. In 37: 'sixty' should be '60'; Ln 120-130: latitude/altitude information may be omitted; Ln 216 and 218: 'equipment's' should be 'equipment'; Ln 225: 'RUTF's' should be 'RUTF'; Ln 228: Table 3, row 1 - add a few examples of antibiotics; Ln 265 and 274: Table 5 and 6, row 1 - 'Exclusively' should be 'exclusive', row 2 - 'Continuing' should be 'continuing'<br><br>•We have made editorials in line 60, 216, 218, 225 accordingly. We omitted the latitude/altitude information in line 120-130. We have added example of antibiotics in table 3. We have also edited the comments raised in table 5 and 6. In addition, we have made editorials throughout the manuscript.
**Additional Information:**	
**Question**	**Response**
**Financial Disclosure**<br><br>Enter a financial disclosure statement that describes the sources of funding for the work included in this submission. Review the submission guidelines for detailed requirements. View published research articles from *PLOS ONE* for specific examples.<br><br>This statement is required for submission and **will appear in the published article** if the submission is accepted. Please make sure it is accurate.	SHG and BSE have received one grant from Irish Aid Ethiopia (https://www.irishaid.ie/what-we-do/countries-where-we-work/our-partner-countries/ethiopia/) through Ministry of Health, Ethiopia. The funders had no role in the study design, data collection and analysis, decision to publish, or preparation of the manuscript.

**Unfunded studies**<br>Enter: *The author(s) received no specific funding for this work.*<br><br>**Funded studies**<br>Enter a statement with the following details:<br>• Initials of the authors who received each award<br>• Grant numbers awarded to each author<br>• The full name of each funder<br>• URL of each funder website<br>• Did the sponsors or funders play any role in the study design, data collection and analysis, decision to publish, or preparation of the manuscript?<br>• **NO** - Include this sentence at the end of your statement: *The funders had no role in study design, data collection and analysis, decision to publish, or preparation of the manuscript.*<br>• **YES** - Specify the role(s) played.<br><br>* typeset	
**Competing Interests**<br><br>Use the instructions below to enter a competing interest statement for this submission. On behalf of all authors, disclose any competing interests that could be perceived to bias this work—acknowledging all financial support and any other relevant financial or non-financial competing interests.<br><br>This statement **will appear in the published article** if the submission is accepted. Please make sure it is accurate. View published research articles from *PLOS ONE* for specific examples.	The authors have declared that no competing interests exist.

**NO authors have competing interests**<br><br>Enter: *The authors have declared that no competing interests exist.*<br><br>**Authors with competing interests**<br><br>Enter competing interest details beginning with this statement:<br><br>*I have read the journal's policy and the authors of this manuscript have the following competing interests: [insert competing interests here]*<br><br>* typeset	
**Ethics Statement**<br><br>Enter an ethics statement for this submission. This statement is required if the study involved:<br><br>• Human participants<br>• Human specimens or tissue<br>• Vertebrate animals or cephalopods<br>• Vertebrate embryos or tissues<br>• Field research<br><br>Write "N/A" if the submission does not require an ethics statement.<br><br>General guidance is provided below. Consult the submission guidelines for detailed instructions. **Make sure that all information entered here is included in the Methods section of the manuscript.**	Ethical clearance was obtained from the Research Ethical Committee of School of Public Health, Addis Ababa University. In addition, permission was obtained from relevant Federal and local government offices. Written informed consent was obtained from the participants after the necessary explanation about the purpose, procedures, benefits and risk of the study have been made. The respondents' right to refuse few or all of the questions was respected at all times. Privacy of participants and confidentiality of the information found was kept.

**Format for specific study types**<br><br>**Human Subject Research (involving human participants and/or tissue)**<br>• Give the name of the institutional review board or ethics committee that approved the study<br>• Include the approval number and/or a statement indicating approval of this research<br>• Indicate the form of consent obtained (written/oral) or the reason that consent was not obtained (e.g. the data were analyzed anonymously)<br><br>**Animal Research (involving vertebrate animals, embryos or tissues)**<br>• Provide the name of the Institutional Animal Care and Use Committee (IACUC) or other relevant ethics board that reviewed the study protocol, and indicate whether they approved this research or granted a formal waiver of ethical approval<br>• Include an approval number if one was obtained<br>• If the study involved *non-human primates*, add *additional details* about animal welfare and steps taken to ameliorate suffering<br>• If anesthesia, euthanasia, or any kind of animal sacrifice is part of the study, include briefly which substances and/or methods were applied<br><br>**Field Research**<br><br>Include the following details if this study involves the collection of plant, animal, or other materials from a natural setting:<br>• Field permit number<br>• Name of the institution or relevant body that granted permission	
**Data Availability**<br><br>Authors are required to make all data underlying the findings described fully available, without restriction, and from the time of publication. PLOS allows rare exceptions to address legal and ethical concerns. See the PLOS Data Policy and FAQ for detailed information.	Yes - all data are fully available without restriction

A Data Availability Statement describing where the data can be found is required at submission. Your answers to this question constitute the Data Availability Statement and **will be published in the article**, if accepted.<br><br>**Important:** Stating 'data available on request from the author' is not sufficient. If your data are only available upon request, select 'No' for the first question and explain your exceptional situation in the text box.<br><br>Do the authors confirm that all data underlying the findings described in their manuscript are fully available without restriction?	
**Describe where the data may be found in full sentences. If you are copying our sample text, replace any instances of XXX with the appropriate details.**<br><br>• If the data are **held or will be held in a public repository**, include URLs, accession numbers or DOIs. If this information will only be available after acceptance, indicate this by ticking the box below. For example: *All XXX files are available from the XXX database (accession number(s) XXX, XXX.*).<br>• If the data are all contained **within the manuscript and/or Supporting Information files**, enter the following: *All relevant data are within the manuscript and its Supporting Information files.*<br>• If neither of these applies but you are able to provide **details of access elsewhere**, with or without limitations, please do so. For example:<br><br>*Data cannot be shared publicly because of [XXX]. Data are available from the XXX Institutional Data Access / Ethics Committee (contact via XXX) for researchers who meet the criteria for access to confidential data.*<br><br>*The data underlying the results presented in the study are available from (include the name of the third party*	All relevant data are within the manuscript and its Supporting Information files.

Powered by Editorial Manager® and ProduXion Manager® from Aries Systems Corporation

and contact information or URL).<br>• This text is appropriate if the data are owned by a third party and authors do not have permission to share the data.<br><br>* typeset	
Additional data availability information:	

Cover letter

October 13, 2020

Editorial Department of PLOS ONE

Dear Editor-in-Chief

We are submitting the revised version of our original research article entitled, "Landscape analysis of nutrition services at Primary Health Care Units (PHCUs) in four districts of Ethiopia." for consideration by PLOS ONE.

Evidence suggests the first 1,000 days represent a window of opportunity from conception through first 2 years after birth. Although good nutrition and healthy growth during the first 1000days have lasting benefit throughout life, the structural readiness of health facilities and nutrition services provision during the first 1000 days in Ethiopia is not well understood

We have assessed the structural readiness of health facilities and the extent of nutrition service provision in four regions of Ethiopia. Our findings revealed poor structural readiness and gap in nutrition services provision.

We confirm that this work is original and has not been published elsewhere, nor is it currently under consideration for publication elsewhere. Additionally, all of the authors have approved the contents of this paper and have agreed to the PLOS ONE 's submission policies. We have uploaded the minimal data set of the study as a supporting information.

We have no conflicts of interest to disclose.

Thank you for your consideration of this manuscript.

Sincerely,

Esete Habtemariam, MPH
Department of Nutrition and Dietetics, School of Public Health,
Addis Ababa University

Ethiopia.

Landscape analysis of nutrition services at Primary Health Care Units (PHCUs) in four districts of Ethiopia.

Esete Habtemariam Fenta[1*], Bilal Shikur Endris[1], Yalemwork Getenet Mengistu[1], Fekadu Elias Sadamo[2], Endashaw Hailu Gelan[1], Tsegaye Gebrezgher Beyene[1], Seifu Hagos Gebreyesus[1]

[1] Department of Nutrition and Dietetics, School of Public Health, College of Health Sciences, Addis Ababa University.

[2] Department of Reproductive Health and Nutrition, School of Public Health, College of Health Sciences, Wolaita Sodo University

[*] esete.f@gmail.com (EHF)

Abstract

Background: Good nutrition and healthy growth during the first 1000days have lasting benefit throughout life. For this, equally important is the structural readiness of health facilities. However, structural readiness and nutrition services provision during the first 1000 days in Ethiopia is not well understood. The present study was part of a broader implementation research aimed at developing model nutrition districts by implementing evidence based, high impact and cost-effective package of nutrition interventions through the continuum of care. This study was aimed at assessing structural readiness of health facilities and the extent of nutrition service provision in the implementation districts.

Methods: This assessment was conducted in four districts of Ethiopia. We used mixed method; a quantitative study followed by qualitative exploration. The quantitative part of the study addressed two-dimensions, structural readiness and process of nutrition service delivery. The first dimension assessed attributes of context in which care is delivered by observing availability of essential logistics. The second dimension assessed the service provision through direct observation of care at different units of health facilities. For these dimensions, we conducted a total of 380 observations in 23 health centers and 33 health posts. The observations were conducted at the Integrated Management of Neonatal and Childhood Illnesses unit, immunization unit, Antenatal care unit and Postnatal care unit. The qualitative part included a total of 60 key informant interviews with key stakeholders and service providers.

Result: We assessed structural readiness of 56 health facilities. Both quantitative and qualitative findings revealed poor structural readiness and gap in nutrition services provision. Health facilities lack essential logistics which was found to be more prominent at health posts compared to health

centers. The process evaluation showed a critical missed opportunity for anthropometric assessment and preventive nutrition counselling at different contact points. This was particularly prominent at immunization unit (where only 16.4% of children had their weight measured and only 16.2% of mothers with children under six month of age were counselled about exclusive breastfeeding). Although 90.4% of pregnant women who came for antenatal care were prescribed iron and folic acid supplementation, only 57.7% were counselled about the benefit and 42.4% were counselled about the side effect. The qualitative findings showed major service provision bottlenecks including non-functionality of the existing district nutrition coordination body and technical committees, training gaps, staff shortage, high staff turnover resulting in work related burden, fatigue and poor motivation among service providers.

Conclusion: We found a considerable poor structural readiness and gaps in delivering integrated nutrition services with a significant missed opportunity in nutrition screening and counselling. Ensuring availability of logistics and improving access to training might improve delivery of nutrition services. In addition, ensuring adequate human resource might reduce missed opportunity and enable providers to provide a thorough preventive counselling service.

Key words: 1000days nutrition, Landscape analysis, Nutrition service evaluation, PHCU, Ethiopia

Background

Maternal and child undernutrition remain prevalent with important consequence in low-income and middle-income countries [1,2]. According to the 2018 World Health Organization (WHO) African region data, between 2000 and 2015 nine countries have prevalence rate of maternal underweight above 15% and exceeds 20% in Ethiopia [3]. Undernutrition is responsible for 45% of all under five child deaths, representing more than 3 million deaths per year [1]. Ethiopia has recorded remarkable improvements in health status and human centered development over the past two decades. Under five mortality rate decreased from 203 per 1000 live birth in 1990 to 58 per 1000 live birth in 2016 [4]. With respect to nutrition indicators, between 2000 and 2016, the national stunting prevalence rate declined by 20% from 58% to 38%, whilst underweight declined by 17% from 41% to 24%, and wasting from 12% to 10% [5].

Even though there is an improvement in undernutrition over the years, stunting, wasting and underweight remain to be prevalent and important public health problem in Ethiopia. In an effort to accelerate the reduction of undernutrition, the Government of Ethiopia developed national nutrition strategy and national nutrition programs (NNP). The revised NNP aims to strategically address nutrition problem by focusing on the life cycle approach to map key actions needed to improve the nutritional status of women and children starting with the first thousand days including nutrition actions for in and out of school adolescents [6]. The first 1,000 days represent a window of opportunity from conception through first 2 years after birth [7,8]. It represents a life window where nutritional deficiencies in the first 1000days can exert an overwhelming impacts [9–11].

It has been identified good nutrition and healthy growth during this period have lasting benefit throughout life by helping avert child mortality and lifelong disease burden, maximizing growth, enabling children to reach their cognitive and developmental potential, particularly when combined with psychosocial stimulation [8,9,11–16]. However, it is not enough to know that a nutrition intervention is efficacious; it is also necessary to identify and address barriers and enablers for effective implementation of interventions under large-scale, real-world conditions.

To this effect, Addis Ababa University with the Federal Ministry of Health (FMOH) of Ethiopia and Irish Aid conducted an implementation research to develop four model nutrition districts. The overarching goal of the project was to create four model districts which implement evidence based, high impact and cost-effective package of nutrition interventions through the continuum of care; during delivery and through to infancy and up to 24 months old, from adolescent to pregnancy (motherhood). This study was conducted as part of the implementation research with the aim of assessing structural readiness of health facility and extent of provision of nutrition services at different contact points in four implementation districts.

Materials and methods

Study setting

The present study was part of a broader implementation research by Addis Ababa University in collaboration with the FMOH of Ethiopia and Irish Aid. The aims of the implementation research were; (i) Understanding barriers and facilitators of comprehensive and integrated nutrition service at primary health care unit (PHCU) level;(ii) Developing implementation protocol based on the finding from desk review, rapid assessment and barrier and facilitator analysis; (iii) Piloting the

implementation protocol on selected kebeles; and (iv) Program learning and optimizing the implementation to create model nutrition districts.

This study was conducted with the aim of assessing the overall nutritional services provision in selected districts. It was conducted in four regions of Ethiopia namely Amhara, Oromia, Tigray and Southern Nations, Nationalities and Peoples' Region (SNNPR). The districts included were South Achefer, Kombolcha, Raya Azebo and Offa respectively. The estimated catchment population for these districts were 161,644, 189,945, 159,347 and 135,136 respectively. There were a total of 26 health centers and 84 health posts in the four selected districts. Of which, 23 health centers and 33 health posts were selected randomly for this study. The health centers were staffed by health officers, nurses, pharmacists, laboratory technicians and midwives while the health posts were staffed by health extension workers.

South Achefer district is located in West Gojjam Zone Administration, North-West, Ethiopia. Subsistence agriculture is the main economic activity, and the income of majority of the households depends on agriculture [17] . Kombolcha district is located in Eastern Hararghe Zone, Oromia National Regional State (ONRS) 542 km east of Addis Ababa. About 8% of the population professed Ethiopian Orthodox Christianity [18]. Crop-livestock farming activity is the main source of income and employment to the society, where crop production takes the principal part of income of the society [19]. Raya Azebo district is located in Tigray region 652 km away from Addis Ababa. Ninety percent of the district is considered "midland" (1500–2300 m) and 10% is considered "lowland" (< 1500 m) [20].

Offa district is one of 16 districts in Wolaita Zone and is located at 383 km south of Addis Ababa on the Sodo Gofa main road. The Offa district, altitude ranges 1200 around river Gogara in the south, 2800 m above sea level in the north. [21].

Study design and period

We used a mixed method approach employing a quantitative study followed by a qualitative study. This study was conducted between June-August, 2018.

Sample size and sampling procedure

The sample size for this study was estimated using a sample size formula for a single proportion. The assumptions used to calculate the sample size were; (i) percentage of women who received nutritional counseling about exclusive breast feeding during postnatal care PNC (P) at 50%, (ii) a 95% confidence level and (iii) 5% margin of error. The calculated sample size was 384. We allocated the sample size proportionally across the four districts.

For an in-depth understanding about the overall nutrition service provision, we conducted a total of 60 key informant interviews in four districts. The key informants were district administration head (n = 1), district health office head (n = 2), deputy district health office head (n = 2), district nutrition focal person (n = 4), program officers (n = 6), service providers at health centers (n =24), religious leader (n = 1), women development army (WDA) leader (n = 1), health extension workers (HEW) (n = 9) and PHCU directors (n = 10). We employed a purposeful sampling method to recruit key informants based on experience on nutrition service provision and management.

Data collection tool and procedure

Observation of services

The data collection addressed various dimensions of the comprehensive integrated nutrition services (CINS) provision at different contact points. The first dimension evaluated was the structural readiness. In this dimension, we assessed attributes of context in which care is delivered by observing availability of equipment, guidelines, social behavioral change communication (SBCC) materials and registration and reporting formats. Structural readiness was assessed in a total of 23 health centers and 33 health posts. We also assessed the characteristics of service providers including sex, age, level of education and nutrition training.

The second dimension evaluated was the process of service delivery. In this dimension, we observed the service provision against standard service guideline. This was done through direct observation of service providers while providing nutrition services at difference contact points. We observed contact points including Integrated Management of Neonatal and Childhood Illnesses (IMNCI) unit, immunization unit, antenatal care (ANC) unit and Post-Natal care (PNC) unit. We observed a total of 103 IMNCI, 73 immunization, 94 ANC and 79 PNC service provision. Some of the observed services include anthropometric assessment and interpretation, provision of IFA supplementation, provision of nutrition counselling and essential counselling skill of service providers. We used the CINS guide published by the FMOH to develop observation checklists (S2 Text). We also pretested the tools and made all the necessary modifications prior to data collection.

Key informant interviews

Interview guides were developed with open-ended questions to have high degree of flexibility. Interview guides covered topics on multi-sectoral collaboration, integration of nutrition services, planning of nutrition services, registration and reporting of service provision, supportive supervision, human resource and motivation of service providers (S3 Text).

The date, time and place of interview were arranged following what was most favorable and comfortable for the participants. Interviews were conducted in a quite office and privacy was ensured to enable participants to feel free and express their opinions. All interviews were audio taped and took about an hour on average. All data were collected by the project coordinators assigned in the four districts. All data were reviewed for completeness and consistency.

Statistical analysis

Quantitative analysis

We used Epi data version 3.1 for data entry and the statistical software package STATA version 14 for data cleaning and analysis. Descriptive statistics was run on the different components of the nutritional services. Frequency tables and graphs were used to further describe the data set. Differences in service provider's counselling skill across contact points were reported using Pearson's chi-square (X^2) test. Statistical significance was set at $p<0.05$.

Qualitative data analysis

To analyze the qualitative data, we initially developed field note template. Using this template, we wrote an expanded field note for each interview. Then we developed analysis matrix where the

rows consist the subgroup selected for the study i.e. the respondents and the columns stands for the topic of study (i.e. the question asked). Using this matrix, we then extracted key findings from the field notes. Quotations were used to further illustrate the responses of the respondents on important issues.

Ethics approval and consent to participate

Ethical clearance was obtained from the Research Ethical Committee of School of Public Health, Addis Ababa University. In addition, permission was obtained from relevant Federal and local government offices. Written informed consent was obtained from the participants after the necessary explanation about the purpose, procedures, benefits and risk of the study have been made. The respondent's right to refuse few or all of the questions was respected at all times. In addition, privacy of participants and confidentiality of the information obtained was kept.

Results

Structural readiness

Availability of essential equipment at health facility. The structural readiness of health centers for nutrition service delivery is presented in table 1. As indicated, the majority of ANC consultancy room (21/23) were equipped with weighing scale, while only (17/23) and (14/23) IMNCI and immunization room were equipped with weighing scale respectively. Height scale was available in (16/23) IMNCI and ANC consultation room while only (8/23) immunization room were equipped with height scale.

Table 1: Availability of equipment, guidelines and record keeping registers at health centers (n=23).

	Immunization	IMNCI	ANC	Delivery
**Anthropometric equipment**				
Baby weighing scale	18	18	NA	23
Adult weighing scale	14	17	21	14
Height/length board	8	16	16	0
MUAC tape	19	23	19	16
**Counselling guide**				
AMIYCN guideline	10	10	8	8
**Registration book**				
Registration book and reporting format	23	23	23	23

*NA not applicable

The structural readiness of health post for nutrition service delivery is shown in table 2. Most of the health posts had baby weighing scale (32/33), MUAC tape (33/33) and reporting formats (30/33). We also observed a critical shortage of equipment and guidelines and only few of the health posts had Adolescent, Maternal, Infant and Young Child Nutrition (AMIYCN) guideline (9/33), height board (8/33) and demonstration equipment (6/33).

Table 2: Availability of equipment, guidelines and record keeping registers at health posts (n=33)

Equipment	Available and functional	Not available
Baby weighing scale	32	1
MUAC tape	33	-
Reporting format	30	3
ANC registration book	27	6
Child card/record	23	10

Adult weighing scale	20	13
SAM guide line	19	14
PNC registration book	15	18
AMIYCN guide line	9	24
Height/length board	8	25
Demonstration equipment	6	27

Table 3 shows availability of medical supplies for nutrition service in health centers and health posts. We observed that most of the health centers and health posts had adequate supply of drugs such as antibiotics, ORS, Iron folic acid, Zinc and Vitamin A. However, supplies such as Albendazole and RUTF were not available in nearly quarter of the health centers observed. It is important to note that Daily ration is nearly absent in all health centers and health posts. Only one health center and seven health posts had the daily ration at the time of observation.

Table 3: Availability of drug supply for nutrition services at health centers and health posts.

List of drugs	Health center (n=23)	Health post (n=33)
Antibiotics (Amoxicillin, Cotrimoxazole)	23	33
ORS	23	32
Iron folic acid (60mg elemental iron + 0.4µg folic acid)	23	31
Zinc (20mg)	23	30
Vitamin A (50,000IU)	21	30
Albendazole (400mg)	17	29
RUSF	16	20
RUTF F-75 (75kcal/100ml)	10	NA
RUTF F-100 (100kcal/100ml)	8	NA

Daily rations (TSF)	1	7

*NA not applicable

Nutrition training of health care providers: As shown in table 4, more than half (54.5%) of ANC service providers were clinical nurses/midwives and were predominantly female (80.7%). In addition, 53.1% of service provider in IMNCI were clinical nurses/midwives and were predominantly male (68.7%). Less than half of service providers in ANC (40.4%), PNC (40.4%) and IMNCI (42.2%) received training on nutrition.

Table 4: Characteristics of health care providers delivering ANC, PNC, immunization and IMNCI at health centers and health posts

	**ANC (n=57)**	**PNC (n=52)**	**Immunization (n=41)**	**IMNCI (n=64)**
**Sex of providers**				
Male	11 (19.3%)	9 (17.3%)	12 (29.3%)	44 (68.7%)
Female	46 (80.7%)	43 (82.7%)	29 (70.7%)	20 (31.3%)
**Provider's Age (mean ±SD)**	26.4 ± 3.68	27.3± 4.26	28.75± 5.05	28.2± 3.35
**Level of education**				
Bsc (Health officer, nurse and midwife	15 (26.3%)	9 (17.3%)	4 (9.7%)	22 (34.4%)
Diploma (Nurse and midwife	31 (54.4%)	30 (57.7%)	18 (43.9%)	34 (53.1%)
HEW	11 (19.3%)	13 (25%)	19 (46.4%)	8 (12.5%)
**Nutrition training**				
Trained	23 (40.4%)	21 (40.4%)	27 (65.8%)	27 (42.2%)
Not trained	34 (59.6%)	31 (59.6%)	14 (34.2%)	37 (57.8%)

Process evaluation

Anthropometric assessment

We assessed the nutritional service delivery by observing a total of 103 IMNCI, 73 immunization, 94 ANC and 79 PNC service provisions. Figure 1 shows result from observation of anthropometric assessment at IMCNI service point. We found that, only 62 (60.2%) children had their weight measured, 38 (36.9%) had their height measured and 60 (58.3%) children had their MUAC measured. Of those children whose anthropometric measurement was taken, correct interpretation was conducted for only 32 (51.6%) children using the weight for age Z-score and for 28 (73.7%) children using height for age Z-score. Correct interpretation using MUAC cutoff point was conducted for only 39 (65%) children. At immunization service unit, only 12 children had their weight measured while none of the children had their height measured.

We observed a total of 94 ANC and 79 PNC service provision to assess the nutritional service delivery for mothers. We found that only 75 (79.8%) mothers had their weight measured and 41 (43.6%) had their MUAC measured at the ANC service point. In addition, only 15 (15.9%) mothers had their height measured. Of those mothers whose MUAC was measured, correct interpretation using MUAC cutoff was conducted for only 34 (82.9%) mothers.

Nutrition counselling provision

Table 5 shows nutrition counselling provided to mothers who came for IMNCI service. Out of 31 mothers with children under six months of age who visited IMNCI, only 19 (61.3%) were counselled on exclusive breastfeeding for the first six months. We also found that counselling on complementary feeding is suboptimal. For example, of the total number of children illegible for counselling on initiation of complementary feeding, only 13 (42%) mothers were counselled about

initiation of complementary food at six months. In addition, only 15 (20.8%) were advised on diversity i.e. to feed a child from animal source foods, and 15 (17.5%) were advised on increasing the frequency of feeding with age.

Table 5: Proportion of mother receiving nutrition counselling at IMNCI service provision point.

Nutrition counselling during IMNCI service (n=103)	Frequency (%)
**Counselling on breastfeeding**	
Advised on exclusive breastfeeding for the first 6 months (n=31)	19 (61.3%)
Advised on continuing frequent, on-demand breastfeeding until 2 years of age (n=84)	27(32.1%)
**Counselling on complementary feeding**	
Advised on initiation of complementary food at 6 months (n=31)	13(41.9%)
Advised on diversity to feed a child from grain, roots and tubers (n=72)	25(34.7%)
Advised on diversity to feed a child from legumes and nuts (n=72)	23(31.9%)
Advised on increasing fluid intake during illness, frequent breastfeeding, and encourage the child to eat soft, varied, appetizing, favorite foods (n=103)	26(25.2%)
Advised on diversity to feed a child from fruits and vegetables (n=72)	18(25.0%)
Advised on diversity to feed a child from animal source foods (n=72)	15(20.8%)
Advised on gradually increasing food consistency as the infant gets older (n=72)	16(22.2%)
Advised on the practice responsive feeding (n=103)	18(20.8%)
Advised on increasing the frequency of feeding with age(n=72)	15(17.5%)
**Counselling on WASH**	
Advised on personal and food hygiene (hand washing before food preparation, after using toilet, during feeding a child (n=103)	15(14.6%)
Advised on proper storage of food (n=103)	10(9.7%)

Table 6 shows the proportion of mothers receiving nutrition counselling at the immunization service point. We found that, out of 68 mothers with children under six months of age who visited immunization unit, only 11 (16.2%) were counselled to exclusively breastfeed their child for six months and only 9 (13.2%) mothers were counselled about initiation of complementary feeding at 6 months. During the observation at immunization unit, we only had 5 mothers with children above the age of six months and no nutritional counselling was given for these mothers.

Table 6: Proportion of mothers receiving nutrition counselling at the immunization service provision point.

Nutrition counselling during Immunization service (n=73)	Frequency (%)
**Counselling on breastfeeding**	
Advised on exclusive breastfeeding for the first 6 months (n=68)	11(16.2%)
Advised on continuing frequent, on-demand breastfeeding until 2 years of age (n=73)	5(6.9%)
**Counselling on complementary feeding**	
Advised on initiation of complementary food at 6 months (n=68)	9(13.2%)
Advised on increasing fluid intake during illness, frequent breastfeeding, and encourage the child to eat soft, varied, appetizing, favorite foods (n=73)	4(5.5%)
Advised on the practice responsive feeding (n=73)	3(4.1%)
Advised on increasing the frequency of feeding with age(n=5)	
Advised on gradually increasing food consistency as the infant gets older (n=5)	0(0.0%)
Advised on diversity to feed a child from grain, roots and tubers (n=5)	0(0.0%)
Advised on diversity to feed a child from legumes and nuts (n=5)	0(0.0%)
Advised on diversity to feed a child from fruits and vegetables (n=5)	0(0.0%)
Advised on diversity to feed a child from animal source foods (n=5)	0(0.0%)
**Counselling on WASH**	

Advised on personal and food hygiene (hand washing before food preparation, after using toilet, during feeding of a child (n=73)	4(5.5%)
Advised on proper storage of food (n=73)	1(1.4%)

Table 7 shows various components of nutrition counselling provided to mothers who came for ANC follow-up. We observed suboptimal nutrition counselling service provided to pregnant mothers against the counselling standards set at ANC clinics. A total of 74 (78.7%) mothers were counselled on having one extra meal every day. While 46 (48.9%) mothers were counselled on having variety of food, 39 (41.5%) were counseled on the importance of having variety of food. Counselling on what to avoid such as eating raw food and what to limit such as caffeine was given for only for 4/94 and 3/94 mothers respectively. We have also observed that only 18 (19.2%) mothers were counselled on maternal health complications due to under nutrition during pregnancy.

Table 7: Proportion of mothers receiving nutritional counselling at antenatal care service provision point.

**Nutrition counselling (n=94)**	**Frequency (%)**
Advised the mother to have one extra meal/ snack every day	74 (78.7%)
Advised the mother to eat from variety of food groups using locally available food	46 (48.9%)
Advised the mother about the importance of eating variety of food groups	39 (41.5 %)
Advised the mother to have adequate fluid intake (*at least 8 glasses of fluid everyday*)	29 (30.9%)
Advised the mother about fetal complications of under nutrition during pregnancy	20 (21.3%)
Advised the mother about having seasonal fruits and vegetables everyday	19 (20.2%)
Advised the mother about maternal complications of under nutrition	18 (19.2%)

Advised the mother about the importance of exclusive breastfeeding for six month (4th visit) (n=21)	8 (38.1%)
Advised the mother on the importance of early initiation of breastfeeding (4th visit) (n=21)	4 (19%)
Advised the mother to avoid pre-lacteal feeding (4th visit) (n=21)	3 (14.3%)
Advised the mother about adding iodized salt when serving food (after cooking)	13 (13.8%)
Advised the mother on importance of feeding colostrum (4th visit) (n=21)	1 (4.8%)
Advised the mother to expose the child to direct sun light (4th visit) (n=21)	1 (4.8%)
Advised the mother what to avoid during pregnancy *(i.e. Alcohol, raw/uncooked foods and vegetables)*	4 (4.3%)
Advised the mother what to limit during pregnancy (*i.e. caffeine, fat)*	3 (3.2%)
**Counselling on WASH (n=94)**	
Advised the mother to keep personal hygiene	46 (48.9%)
Advised the mother to wash hand after toilet use, and before meal preparation	11 (11.7%)
Advised the mother to keep utensils and cooking material clean	9 (9.6%)
Advised the mother to use treated water for drinking	3 (3.2%)
**Counselling on ITN (n=94)**	
Advised on ITN use	34 (36.2%)

Figure 2 shows counselling provided to mother on IFA supplementation during ANC follow up. We found that majority of the mothers 85 (90.4%) who came for ANC were provided IFA prescription. However, of those who were given prescription, only 73 (85.9%) were counselled to take IFA daily for six months. In addition, only 49 (57.7%) mothers were counselled about the benefit of IFA and 36 (42.4%) were counselled on the possible side effect of IFA.

Table 8 shows various components of nutrition counselling provided to mothers who came for PNC service. We have observed 75 (94.9%) mothers were counselled to exclusively breastfeed up

to six months. Regarding maternal nutrition, 63 (79.8%) mothers were counseled to have two extra meal and rest during lactation. Counselling about maternal complication of undernutrition was given for only 27 (34.2%) mothers.

Table 8: Proportion of mother receiving nutritional counselling at postnatal care service provision point.

Nutritional counselling (n=79)	Frequency (%)
Advised mother to exclusively breastfeed up to six month	75 (94.9%)
Advised the mother about extra meal and rest during lactation	63 (79.8%)
Advised mother to breastfeed on demand	62 (78.5%)
Advised the mother on the importance of exclusive breastfeeding for six month	56 (70.9%)
Advised mother on correct positioning and attachment during breastfeeding	54 (68.4%)
Advised the mother to eat from variety of food groups (locally available food)	48 (60.8%)
Advised the mother to eat food rich in iron	42 (53.2%)
Advised the mother to eat a diet that is rich in protein and fluid	38 (48.1%)
Advised the mother on the importance of eating variety of food groups	37 (46.8%)
Advised mother to avoid all dietary restriction if any	35 (44.3%)
Advised the mother about maternal complications of under nutrition during lactation	27 (34.2%)
Advised the mother to have adequate fluid intake (at least 8 glasses everyday)	21 (26.6%)
Advised the mother to have seasonal fruits and vegetables everyday	14 (17.7%)
Advised the mother about adding iodized salt when serving food (after cooking)	13 (16.5%)
**Counselling on WASH (n=79)**	
Advised the mother to use treated water for drinking	15 (18.9%)
Advised the mother about safe disposal of potentially infectious soiled pads or other materials	14 (17.7%)
Advised the mother to wash hand after toilet use, and before meal preparation, after cleaning baby, before feeding ,before breast feeding	12 (15.2%)
Advised the mother to keep utensils and cooking material clean	15 (18.9%)
Advised the mother to keep personal hygiene	40 (50.6%)

Counselling skill of service providers

Table 9 shows the counselling skill of service providers working at IMNCI and immunization service points. Listening to what the clients have to say was higher among service providers at IMNCI 91.2% compared to service providers at immunization 75.3% *(P<0.05)*. Similarly, recognizing and praising what clients were doing correctly was higher among service providers at IMNCI 23.3% compared to service providers at immunization 9.6% *(P<0.05)*. During counselling, providers identified key difficulties for only 20.4% and 6.8% of the clients at IMNCI and immunization respectively *(P<0.05)*.

Table 9: Counselling skill of service providers during provision of counseling at IMNCI and immunization service points.

**Counselling**	**IMNCI (n=103)**	**Immunization (n=73)**	**Chi2 P-value**
Service provider listens to what the client has to say	94 (91.3%)	55 (75.3%)	**0.004**
Service provider recorded history of the client	87 (84.5%)	51 (69.9%)	**0.020**
Service provider used responses and gestures that show interest	84 (81.6%)	54 (73.9%)	0.228
Service provider avoided using judging words	84 (81.6%)	52 (71.2%)	0.107
Service provider Identified key difficulties (if any) and selects with the client the most important one to work on	21 (20.4%)	5 (6.8%)	**0.013**
Of those mothers with key difficulties service provider discussed options on key difficulties the client raised	17 (80.9%)*	5 (100%)*	0.056
Service provider recommends and negotiates do-able actions to help the client select the best option to try depending on her context and resources	16 (76.2%)*	5 (100%)*	0.080

Service provider helped the client agree to try one of the options and asks them to repeat the agreed-upon do-able action	15 (71.4%)*	5 (100%)*	0.112
Service provider let the client talk through her concerns before correcting information	59 (57.3%)	31 (42.5%)	0.053
Service provider recognized and praised what the client is doing correctly	24 (23.3%)	7 (9.6%)	**0.019**

* The denominator is the number of clients identified with key difficulties

Table 10 shows the counselling skill of service providers working at ANC and PNC service points. Listening to what the clients have to say was higher among service providers at ANC 97.9% compared to service providers at PNC 89.9% *(P<0.05)*. Similarly, identifying key difficulties the clients have was higher among service providers at ANC 40.4% compared to service providers at PNC 16.5% *(P<0.001)*. However, discussing about options for the identified difficulties was higher among service providers at PNC 92.3% compared to service providers at ANC 73.7% *(P<0.05)*. Similarly, recommending doable action for the identified problem was higher among service providers at PNC 76.9% compared to service providers at ANC 68.4% (*P<0.05*).

Table 10: Counselling skill of service providers during provision of counseling at ANC and PNC service points.

**Counselling**	**ANC (n= 94)**	**PNC (n=79)**	**Chi2 P-value**
Service provider listens to what the client has to say	92 (97.9 %)	71 (89.9%)	**0.045**
Service provider used responses and gestures that show interest	89 (94.7%)	71 (89.9%)	0.232
Service provider let the client talk through her concerns before correcting information	84 (89.4%)	63 (79.8%)	0.078

Service provider avoided using judging words	83 (88.3%)	63 (79.8%)	0.123
Service provider Identified key difficulties (if any) and selects with the client the most important one to work on	38 (40.4%)	13 (16.5%)	**0.001**
Of those mothers with key difficulties service provider discussed options on key difficulties the client raised	28 (73.7%)*	12 (92.3%)*	**0.023**
Service provider recommends and negotiates do-able actions to help the client select the best option to try depending on her context and resources	26 (68.4%)*	10 (76.9) *	**0.015**
Service provider helped the client agree to try one of the options and asks them to repeat the agreed-upon do-able action	26 (68.4%)*	9 (75%)*	0.491
Service provider recognized and praised what the client is doing correctly	43 (45.7%)	25 (31.7%)	0.059
Service providers recorded history of the client	71 (70.9%)	56 (70.9%)	0.083

* The denominator is the number of clients identified with key difficulties

Qualitative Result

A total of 60 key informants participated in this study. The participants include district administrative head, district health office head, district health office deputy head, district nutrition focal person, program officers, service providers at health centers, religious leader, WDA, HEW and PHCU heads.

Multi-sectoral collaboration

We found that the Woreda nutrition coordination body (WNCB), Woreda nutrition technical committee (WNTC) and Kebele nutrition technical committee (KNTC) are in place in the four districts. These structures were established following the 1000 days plus program launching. Key informant interview indicated that the issue is not related to the presence of the multi-sectoral

coordination bodies rather it is related to its functionality. It is important to note that neither the coordination body nor the technical committee meet regularly and are productive enough.

One of the key informants explained about the functionality of WNCB, WNTC and KNTC as follows:

> *" The WNCB meetings are not conducted regularly. There is usually third body request to have the meeting. It is not scheduled ahead and usually done hastily. There is unequal commitment of sectors, not planning together and there are no terms of reference. I can say WNTC almost does not exist and needs reestablishment. Regarding KNTC; the HEWs have capacity gap, they are not committed and motivated because of carrier structure problem, inadequate salary, no recognizing/rewarding mechanism, work overload etc. The kebele leaders are not committed as well."*

The functionality of the structure is mainly dependent up on the existence of nutrition projects in the district. All key informants believe that having WNCB is very important for the success of nutrition services. However, there is lack of ownership among different members.

One participant explained the issue of ownership as follows:

> *"There is a tendency of pushing this job aside by the health sector due to different emerging activities and other priorities. In addition, the other sectors are dependent on an external initiation and they don't own this structure as part of their routine job. Apart from this there is also lack of logistic to conduct the meeting effectively…. you now like incentives."*

Another important bottleneck related to the functionality of the WNCB is the absence of shared definitive roles and responsibilities among the members. The participants of this study indicated that the members in the coordination body externalize their responsibility to the health sector and there is an assumption that nutrition is the concern of health sector only.

One of the participant expressed the following:

"Because of other political priorities the coordination body is not operating to the envisioned extent. So mainly, it is the technical committee that is working better currently. However, there are certain still limitation on the technical committee as well. There is a high challenge with ownership of the body as they feel this task is solely the responsibility of the health sector".

Integration of nutrition services

Our assessment shows that there is sub optimal and poor integration of nutrition services with in child and maternal health services at the health centers and health posts. We documented a physical rather than functional integration of nutrition services. For instance, growth monitoring and promotion (GMP), immunization and IMNCI service are given in one room usually by one service provider. However, there is missed opportunity of providing GMP for children who came for immunization and IMNCI service.

"There is no separate room for GMP, IMNCI and immunization at health center level. These three activities are offered in a room by a provider. The same is true for ANC, Delivery and PNC .... However; service providers are not using this opportunity to give nutrition service in an integrated manner."

Human resource, training and motivation of service providers

We have assessed staff adequacy, training and motivation of service providers at different levels in the districts. According to our finding, there is critical staff shortage at each level from district health office to the health post. This staff shortage was more profound at remote health facilities.

" I myself focal for three positions, work in regular and emergency outpatient department (OPD), the health center head also works in OPDs, other staffs also have burden and

generally there is staff shortage; there has been very critical shortage though yesterday one health officer joined the health center. There are only 5 staffs including the head and me. There is shortage of staffs in all departments."

Even though there is shortage at health centers, it was more significant at health posts. The number of the HEWs was not comparable with the number of households living in their catchment area. One participant explained:

"Based on the standard one health extension worker gives service to 2500 populations in her catchment area (1:2500). But the ratio in our woreda is 1: 10,000 to 11,000 populations. This is similar in the health center as well, there is not enough service provider compared to service seekers."

Besides staff shortage, we documented high staff turnover. Similar to staff shortage, the turnover was more prominent in rural health facilities. The turnover was most common among midwives, health officers and HEW.

"This year 8 HEWs left their job and moved to different cities by changing their job. It became a big challenge for the woreda. Some health posts have only one HEW. Currently Zemo health post have no HEW as a result it is closed."

We have identified training gaps in AMIYCN, out-patient therapeutic program (OTP), community-based nutrition (CBN), management of sever acute malnutrition, blended integrated nutrition and counselling skill. Besides, the training opportunities mainly depend on the partner's or training provider organization's objective rather than the need of the health sector in the district. Furthermore, we have identified poor opportunities for health care providers to further their education and future career development.

" …there is no study opportunity. As I mentioned for you earlier, I joined this health center nearly five years back. It is very difficult to talk on this and there is no chance to pursue our education. Even when there are opportunities, there is no fair selection of candidates."

We also evaluated motivation of service provider. We have found poor motivation among service providers. Participants described variety of factors affecting their motivation including access to training, access to further education, timely duty payment, work overload, and inadequate salary.

Planning of nutrition services

The five-year strategic health sector plan is used as a baseline for district health office planning purpose. During planning HEWs, health center heads, personnel from district health office, and delegates from other sectors such as children and women affairs, agriculture, finance etc. are usually involved. However, the planning followed top to bottom approach. This approach does not take in to account the existing situation on the ground. Because of this, service providers had to deal with numbers sent from the top-level officials that doesn't really exist in their catchment area. They have explained this as follows:

"We are facing challenge during planning as we breakdown the figures we took from the woreda to each health post by using conversion factors. Therefore, the plan is not based on the existing situation of the PHCU."

"There was a time where they give us 5000 plans while we have 3000 in our catchment and vice versa so don't get surprised when you see 205% achievement."

Registration and reporting of nutrition services

We observed multiple registration books being used by professionals for different services. Registration format were not available at facilities according to the standard set by the FMOH. We documented shortage of registration books especially at health post level.

> *“We are using self-made registry. This is a burden for us since making the register is tiresome. It also has drawback as we might omit some important variables”.*

Another challenge with registration book was related with portability. Health extension workers used a plain paper to document activities during outreach services because it is difficult to carry the registration books to outreach sites. This has drawback as the HEWs often forget to copy to the registers resulting in poor documentation and inconsistency among reports and registers. In general, there is poor documentation especially at health post level.

> *"There is no documentation problem at health center level. We perform activities and record accordingly......... but there is a gap at health post level. Last time the health center head and I along with kebele leader came across poor documentation. This might be because the HEW are newly assigned since senior HEWs have left their job for different reasons."*

Similar to registration, we have identified challenges related with reporting as well. The challenges were lack of timeliness, reliability, and completeness of the reports.

> *“There was an incident where one of the health extension worker sent 0 ANC on the HMIS report and 20 ANC on the disease report, so we will cross check for such kind of errors and give feedback on how to improve the report in the future”.*

Supportive supervision

The district health office provides supportive supervision to the health centers and health centers provide supportive supervision to health posts. There were two forms of supportive supervision in the district; integrated supportive supervision and program specific supportive supervision. As the name implies, integrated supportive supervision was conducted every quarter by integrating different case team leaders from the district health office. The program specific supervision was conducted whenever the need arises by individuals assigned to a specific program. This type of supervision focuses on specific programs.

> *"There is Integrated Supportive Supervision (ISS) this will be done every quarter where different officers from the Woreda health office supervise health centers, health posts and at least 10 sample households at community level."*

Discussion

The landscape analysis was intended to assess service readiness and nutrition service provision at different service provision points. The result from this study indicated poor structural readiness and gap in nutrition service provisions. Health facilities lacked essential anthropometric instruments, nutrition guidelines, registration and reporting formats. Poor structural readiness was more prominent at health posts compared to health centers. Our process evaluation indicated that there is missed opportunity of anthropometric assessment and preventive nutrition counselling at different contact points. Such problem was more critical at immunization unit than other units. In our qualitative assessment, we found that there is an existing but non-functional Woreda nutrition coordination body and technical committee. Training gaps, staff shortage and high staff turnover

were the main challenges faced by service providers resulting in burden, fatigue and poor motivation.

We have observed poor structural readiness in both health centers and health posts. However, overall facility preparedness was better in health centers compared to health posts. This might be because the health centers provide comprehensive primary health care including promotive, curative and rehabilitative services as compared to health posts. Even though health centers exhibited better structural readiness, the level of readiness varied across different units. For instance, there were greater availability of weighing scale at ANC units than other units. Such type of problems were also common in other countries like Bangladesh where ANC rooms have better structural readiness than other units [22]. This might be because ANC has been practiced well as part of the health service implementation compared to the recently introduced nutrition service implementation in IMNCI [22].

Integration of nutrition service is expected at different contact points such as IMNCI. It is assumed that if screening is performed routinely in curative clinics, it creates a better opportunity for timely detection and intervention. However, our assessment of service provision at IMNCI indicated a missed opportunity for basic nutrition screening and counselling. Indeed, of the total number of children who came for IMNCI service, only 60.2% had their weight measured. It is important to note that the current IMCNI algorithm asks for weight of the child and yet 40% of the children were not weighed. Similarly, a study done in Botswana showed limited nutrition and dietary screening in curative clinics [23]. This missed opportunity could be attributed to lack of anthropometric equipment, poor motivation of staffs, shortage of staff and lack of supervision.

Poor interpretation of anthropometric assessments were also the findings from this study. Without nutrition assessment and correct interpretation, it is unlikely there would be appropriate management of undernutrition and appropriate treatment of other childhood illness [24]. Of those children whose weight was measured at IMNCI unit, correct interpretation using weight for age Z-score was done for only 51.6% of the children. This might be related with capacity gaps, poor commitment and workload among service providers.

The other challenge that we identified was related to provision of preventive services such as proper counselling during clinical contact. Poor nutrition counselling could be a result of high caseload, staff shortage and capacity gap which led to prioritization of the immediate clinical concern rather than focusing on preventive intervention. However, overall nutrition service at IMNCI was relatively better as compared to immunization unit. We have identified a critical missed opportunity at immunization unit where only 16.4% of children had their weight measured while none of the children had their height measured. Lack of essential anthropometric instruments such as weight scale and stadiometer could have been possible reason for lack of appropriate nutritional assessment. In addition to lack of essential equipment, high caseload during immunization day could also be the possible reason for missed opportunity. The trend of provision of immunization was using a fixed day resulting in high caseload making immunization one of the most rushed services. This has resulted in poor nutrition counseling service at immunization unit. Of all the services, the least preventive counselling service was observed in this unit.

Immunization services are often widely available and potentially can support and be supported by additional health interventions. The combined delivery or integration of linked health interventions including GMP, nutrition advises and preventive cares for a more effective way of achieving common health goals [25]. In the study area, there was an opportunity to integrate nutrition

services as there was a physical integration of IMNCI, Immunization and GMP services. These services were given in a single room and by a single provider in most of the health facilities. However, the service provision did not progress beyond the physical integration which resulted in missed opportunities.

The level of provision of essential nutrition services was better at ANC and PNC unit as compared to IMNCI and immunization unit. This is reflected by improved nutrition counselling at ANC and PNC unit. Nutritional assessment was also better at ANC unit. For instance, majority (79.79%) of the mothers at ANC were weighed. We have also observed a relatively better counselling at ANC unit. This improved nutrition service might be due to the preventive nature of ANC service. Since ANC service is preventive than curative it makes it conducive to provide nutrition counselling than other services.

Our study showed prescription of IFA was high however; counselling related to IFA was poor. This finding was similar with a report conducted with the aim of describing and comparing nutrition intervention using service readiness and delivery across different countries. The finding from the study indicated prescription/provision of IFA was higher than counselling service related with IFA. Prescription ranged from 62.2% in Nepal to 87.5% in Senegal. Nonetheless, counselling on benefit of IFA ranged from 18% in Nepal to 72.6% in Tanzania [26]. Counselling on possible side effects was very low ranging from 1.8% in Senegal to 14.2% in Tanzania [26]. Inadequate tools and skill in counselling to support and monitor adherence could be possible reason for the observed result [27]. Poor counselling service about benefits, possible side effects, severity and magnitude of the problem has been identified as barriers for non-adherence of IFA [26,28–30]. A systematic review has identified fear of side effect as the commonest reason for non-adherence which was 46.4% [31]. As a result, counselling addressing benefit and possible side effect with

mitigation strategy to overcome the side effect should be incorporated other than mere provision of the supplement to ensure adherence.

During our assessment, we have identified major challenges faced by service providers. Training gap, staff shortage and high staff turnover were the commonly identified challenges. The training gap was not uniform in all unit; it was more pronounced among health care providers at maternity unit. The other challenge with training is that it was not need based rather it mainly depend on the objective of the organization providing the training. Similar to training, staff shortage and turnover was also not uniform. It was more common at rural health facilities. This might be due personal reasons and service provider's desire to live in town or near to town. Such problems could be possible reasons for poor implementation of services at different unit.

Motivation of staff was one factor for effective delivery of nutrition services. Health extension workers are overloaded with health and other non-health related commitments such as political tasks. This has resulted in poor motivation and fatigue among health extension workers. Apart from the burden of work, lack of access to trainings, lack of timely duty payment, lack of advancement in national career structure and inadequate salary were factors for poor motivation of service providers.

Because of the multi-sectoral nature of nutrition, various stakeholders outside the health needs to be involved, coordinated and linked to gain the synergistic impact for achievement of nutrition interventions [6]. Even though nutrition coordinating bodies and technical committees were established in all the districts, it is not functioning to the desired level. The primary reason for this could be engagement of different sectors in other urgent and political commitments. Even though the sectors will be housed in health sector all member of the coordinating body and technical

committee should be equally accountable [32]. However, this was not the reality in all districts. There was poor ownership and accountability among sectors reflected by externalizing every responsibility to the health sector. This might be a result of lack of strong monitoring and evaluation system in place. In addition, lack of coordination from the federal and regional governments has been identified as a constraint to improve nutrition service delivery in Ethiopia [33]. In order to ensure functionality of coordination body and technical committee, there should be a mechanism to capture data and triangulate from all relevant sectors.

We believe the current study has limitation that needs to be acknowledged. Since we used direct observation, it is difficult to rule out desirability bias. Direct observation during service provision may have encouraged service providers to offer a more thorough service for their clients. However, we believe the impact is minimal since our finding on both the structural readiness and process evaluation were low. The strength of this study was the assessment of both health centers and health posts and the use of multiple methods of data collection techniques including observation, and key informant interviews to assess the structural readiness and provision of nutrition services.

Conclusion and Recommendation

We documented poor structural readiness and gaps to deliver integrated nutrition services in all four districts. There were significant missed opportunities for different nutrition services including nutrition screening and counselling. Poor commitment and accountability of coordination body and technical committee towards nutrition intervention was common. Improving logistics and training may improve delivery of nutrition service. Improving health human resource might reduce missed opportunity that resulted because of high caseload.. It might also ensure adequate time to provide thorough preventive counselling service at all units. Ensuring a clear structure of

accountability along with reporting mechanisms in the existing system might ensure functionality and accountability of the coordinating body and technical committee.

Supporting information

S1 Dataset. The minimal data set of this study

S2 Text. Observation checklist used in this study.

S3 Text. Key informant interview guide used in this study.

Acknowledgment

We are very grateful for the financial support of Irish Aid Ethiopia and the Federal Ministry of Health of Ethiopia. We would like to thank Addis Ababa University, School of Public Health for facilitating the conduct of the research. We appreciate the managers and health care providers for their assistance and cooperation during the assessment. Finally, we would like to thank all study participants who took part in this study.

Reference

1. Black RE, Allen LH, Bhutta ZA, Caulfield LE, de Onis M, Ezzati M, et al. Maternal and child undernutrition: global and regional exposures and health consequences. Lancet. 2008;371(9608):243–60.

2. Black RE, Victora CG, Walker SP, Bhutta ZA, Christian P, De Onis M, et al. Maternal and child undernutrition and overweight in low-income and middle-income countries. Lancet. 2013;382(9890):427–51.

3. World Health Organization. Nutrition in the WHO African Region.

4. UNICEF, WHO World Bank Group and United, Levels and Trends in Child Mortality Report, 2017.

5. Ethiopia Demographic and Health Survey 2016 Central Statistical Agency Addis Ababa, Ethiopia ICF International Calverton, Maryland, USA, 2017.

6. Federal Democratic Republic of Ethiopia - National Nutrition Program, 2016-2020.

7. Bhatia J, Bhutta ZA, Kalhan SC, editors. Maternal and child nutrition: the first 1,000 days. Karger Medical and Scientific Publishers; 2013.

8. RE Black, J Hopkins Bloomberg The lancet series on maternal and child nutrition, executive summary, Lancet 372, 1-10, 2008.

9. Karakochuck CD, Whitfield KC, Green TJ, Kraemer K. The biology of the first 1,000 days. CRC Press; 2017 Sep 22.

10. Schwarzenberg SJ, Georgieff MK. Advocacy for improving nutrition in the first 1000 days to support childhood development and adult health. Pediatrics. 2018;141(2).

11. Beluska-Turkan K, Korczak R, Hartell B, Moskal K, Maukonen J, Alexander DE, et al. Nutritional gaps and supplementation in the first 1000 days. Nutrients. 2019;11(12):1–50.

12. Bellieni C V. The Golden 1,000 Days. J Gen Pract. 2016;04(02):2–5.

13. Maalouf-Manasseh Z, Oot L, Sethuraman K. Food and Nutrition Technical Assistance III Project Giving Children the Best Start in Life: Integrating Nutrition and Early Childhood Development Programming within the First 1,000 Days. SecurenutritionOrg [Internet]. 2016;(January).

14. Lowensohn RI, Stadler DD, Naze C. Current concepts of maternal nutrition. Obstetrical & gynecological survey. Pediatr Emerg Care. 2017;33(12):792–3.

15. Wu G, Bazer F, Cudd T, Meininger C. Recent Advances in Nutritional Sciences-Maternal Nutrition and Fetal Development. Nutr [Internet]. 2004;(13):2169–72.

16. Abu-Saad K, Fraser D. Maternal nutrition and birth outcomes. Epidemiol Rev. 2010;32(1):5–25.

17. Sibhatu KT, Qaim M. Rural food security, subsistence agriculture, and seasonality. PLoS One. 2017;12(10):1–15.

18. CSA (Central Statistical Agency) (2006): Ethiopia Demographic and Health Survey 2005. Addis Ababa, Ethiopia. (2008): Statistical Abstract 2007. Addis Ababa, Ethiopia.

19. BOARD (Bureau of Agriculture and Rural Development) (2009): Compiled Report for Kombolcha. Eastern Hararghe Zone, Oromiya Regional State, Ethiopia.

20. Tesfaye G, Gebresamuel G, Gebretsadik A, Gebrelibanos A, Gebremeskel Y, Hagos T. Participatory rural appraisal report: Raya-Azebo Woreda, Tigray region; 2014.

21. Offa Woreda Burau of Agriculture and Rural Development, 2016.

22. Billah SM, Saha KK, Khan ANS, Chowdhury AH, Garnett SP, Arifeen S El, et al. Quality of nutrition services in primary health care facilities: Implications for integrating nutrition into the health system in Bangladesh. PLoS One. 2017;12(5):1–16.

23. Nnyepi MS. Dietary and Nutrition Screening for Children Seeking curative care in health facilities in Botswana. African Journal of Food, Agriculture, Nutrition and Development. 2006;6(2).

24. Arifeen SE, Bryce J, Gouws E, Baqui AH, Black RE, Hoque DME, et al. Quality of care for under-fives in first-level health facilities in one district of Bangladesh. Bull World Health Organ. 2005;83(4):260–7.

25. World Health Orgnization. GIVS: Global Immunization Vision and Strategy 2006-2015.

World Health Organization;2005.
26. Wang W, Benedict RK, Mallick L. The Role of Health Facilities in Supporting Adherence to Iron-Folic Acid Supplementation During Pregnancy: A Case Study Using DHS and SPA Data in Haiti and Malawi. DHS Working Paper No.160. Rockville, Maryland, USA: ICF. 2019;(August).
27. Siekmans K, Roche M, Kung'u JK, Desrochers RE, De-Regil LM. Barriers and enablers for iron folic acid (IFA) supplementation in pregnant women. Matern Child Nutr. 2018;14(July 2017):1–13.
28. Taye B, Abeje G, Mekonen A. Factors associated with compliance of prenatal iron folate supplementation among women in Mecha district, Western Amhara: A cross-sectional study. Pan Afr Med J. 2015;20:1–7.
29. Assefa H, Abebe SM, Sisay M. Magnitude and factors associated with adherence to Iron and folic acid supplementation among pregnant women in Aykel town, Northwest Ethiopia. BMC Pregnancy Childbirth. 2019;19(1):1–8.
30. Arega Sadore A, Abebe Gebretsadik L, Aman Hussen M. Compliance with iron-folate supplement and associated factors among antenatal care attendant mothers in Misha District, South Ethiopia: Community based cross-sectional study. J Environ Public Health. 2015;2015:1–7.
31. Desta M, Kassie B, Chanie H, Mulugeta H, Yirga T, Temesgen H, et al. Adherence of iron and folic acid supplementation and determinants among pregnant women in Ethiopia: A systematic review and meta-analysis. Reprod Health. 2019;16(1):1-14.
32. Government of the Federal Democratic Republic Of Ethiopia Program Implementation Manual of National Nutrition Program (NNP) - I July 2008 – June 2013 Federal Ministry

of Health. 2013;(June).

33. Sustainability S, Nutrition OF, Assessment IN, Program S. Programming and Integrating Nutrition Assessment ,2018.

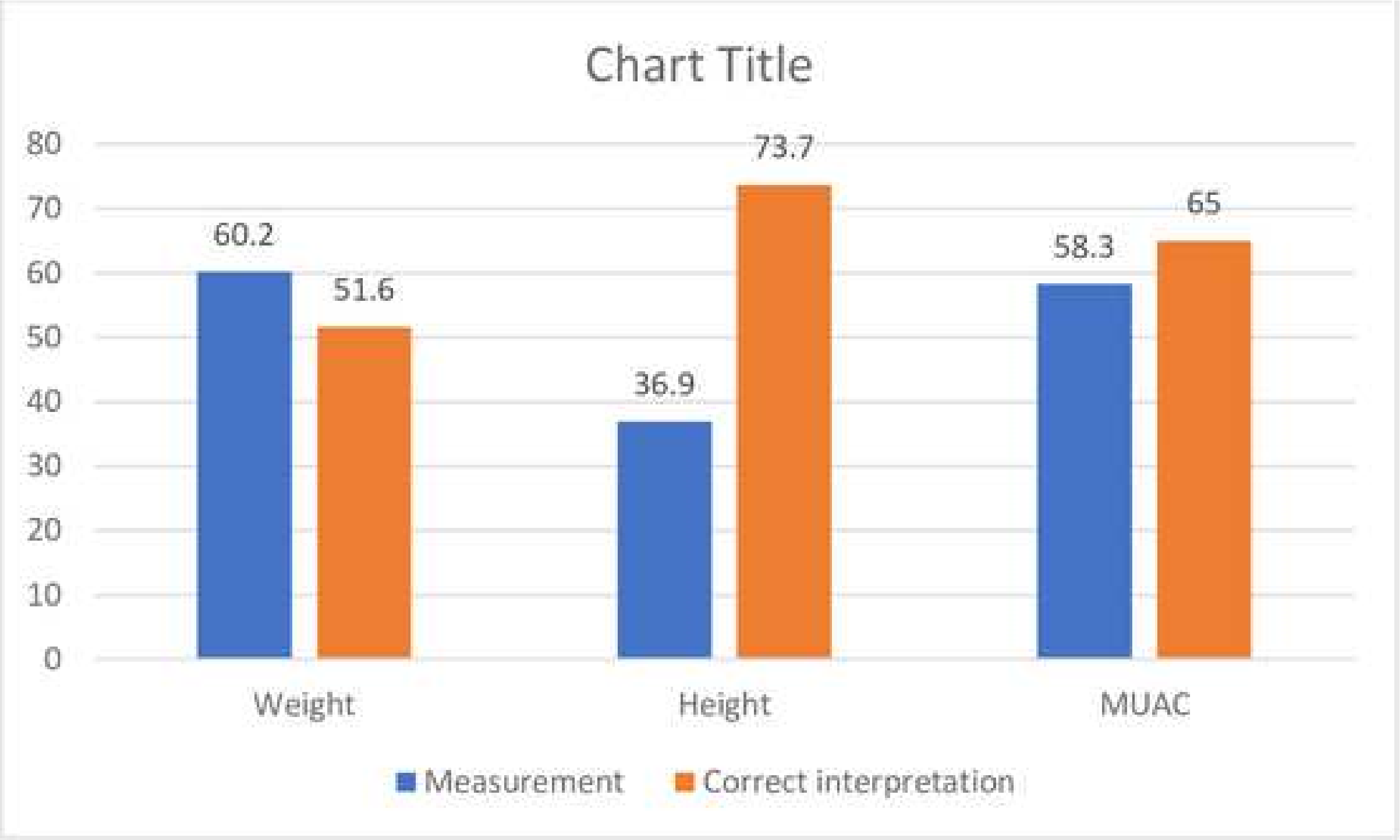

Figure 1: Proportion of children receiving anthropometric measurement during IMNCI service provision

 Click here to access/download;Figure;Figure 2.tif

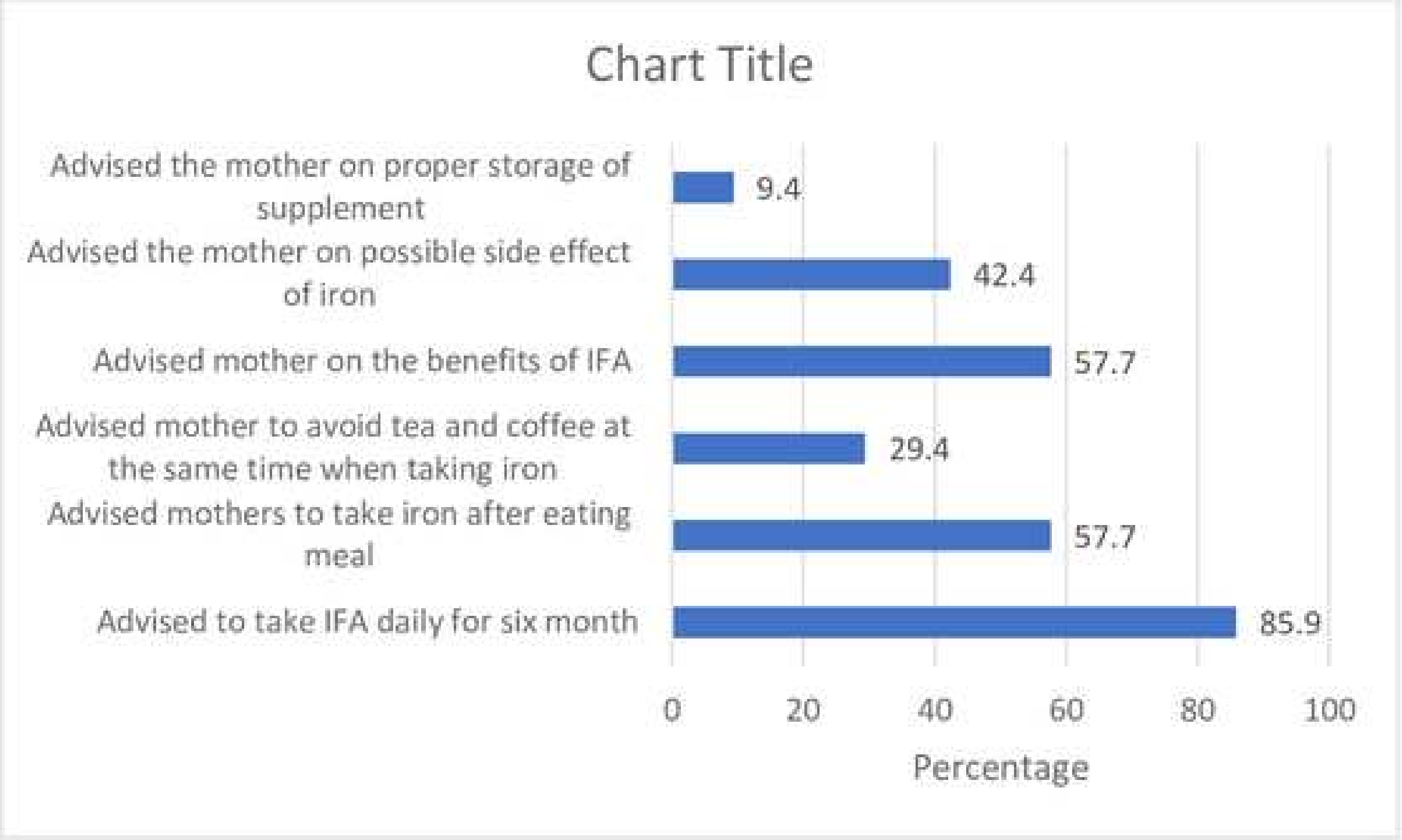

Figure 2: Proportion of pregnant mother receiving counselling on Iron folic acid supplementation during antenatal care service provision.

Dataset

Click here to access/download
Supporting Information
Dataset.xlsx

Observation checklist

Click here to access/download
Supporting Information
Observation check list.docx

Click here to access/download
Supporting Information
Key informant interview guide.docx

Revised Manuscript with Track Changes

Landscape analysis of nutrition services at Primary Health Care Units (PHCUs) in four districts of Ethiopia.

Esete Habtemariam Fenta[1*], Bilal Shikur Endris[1], Yalemwork Getenet Mengistu[1], Fekadu Elias Sadamo[2], Endashaw Hailu Gelan[1], Tsegaye Gebrezgher Beyene[1], Seifu Hagos Gebreyesus[1]

[1] Department of Nutrition and Dietetics, School of Public Health, College of Health Sciences, Addis Ababa University.

[2] Department of Reproductive Health and Nutrition, School of Public Health, College of Health Sciences, Wolaita Sodo University

[*] esete.f@gmail.com (EHF)

Abstract

Background: Good nutrition and healthy growth during the first 1000days have lasting benefit throughout life. For this, equally important is the structural readiness of health facilities. However, structural readiness and nutrition services provision during the first 1000 days in Ethiopia is not well understood. The present study was part of a broader implementation research aimed at developing model nutrition districts~~services~~ by implementing evidence based, high impact and cost-effective package of nutrition interventions through the continuum of care. This study was aimed at assessing structural readiness of health facilities and the extent of nutrition service provision in the implementation districts.

Methods: This assessment was conducted in four districts of Ethiopia. We used mixed method; a quantitative study followed by qualitative exploration. The quantitative part of the study addressed two-dimensions, structural readiness and process of nutrition service delivery. The first dimension assessed attributes of context in which care is delivered by observing availability of essential logistics. The second dimension assessed the service provision through direct observation of care at different units of health facilities. For these dimensions, we conducted a total of 380 observations in 23 health centers and 33 health posts. The observations were conducted at the Integrated Management of Neonatal and Childhood Illnesses unit, immunization unit, Antenatal care unit and Postnatal care unit. The qualitative part included a total of 60~~sixty~~ key informant interviews with key stakeholders and service providers.

Result: We assessed structural readiness of 56 health facilities. Both quantitative and qualitative findings revealed poor structural readiness and gap in nutrition services provision. Health facilities lack essential logistics which was found to be more prominent at health posts ~~level~~ compared to

health centers. The process evaluation showed a critical missed opportunity for anthropometric assessment and preventive nutrition counselling at different contact points. This was particularly prominent at immunization unit (where only 16.4% of children had their weight measured and only 16.2% of mothers with children under six month of age were counselled about exclusive breastfeeding). Although 90.4% of pregnant women who came for antenatal care were prescribed iron and folic acid supplementation, only 57.7% were counselled about the benefit and 42.4% were counselled about the side effect. The qualitative findings showed major service provision bottlenecks including non-functionality of the existing district nutrition coordination body and technical committees, training gaps, staff shortage, high staff turnover resulting in work related burden, fatigue and poor motivation among service providers.

Conclusion: We found a considerable poor structural readiness and gaps in delivering integrated nutrition services with a significant missed opportunity in nutrition screening and counselling. Ensuring availability of logistics and improving access to training might improve delivery of nutrition services. In addition, ensuring adequate human resource might reduce missed opportunity and enable providers to provide a thorough preventive counselling service.

Key words: 1000days nutrition, Landscape analysis, Nutrition service evaluation, PHCU, Ethiopia

Background

Maternal and child undernutrition remain prevalent with important consequence in low-income and middle-income countries [1,2]. According to the 2018 World Health Organization (WHO) African region data, between 2000 and 2015 nine countries have prevalence rate of maternal underweight above 15% and exceeds 20% in Ethiopia [3]. Undernutrition is responsible for 45% of all under five child deaths, representing more than 3 million deaths per year [1]. Ethiopia has recorded remarkable improvements in health status and human centered development over the past two decades. Under five mortality rate decreased from 203 per 1000 live birth in 1990 to 58 per 1000 live birth in 2016 [4]. With respect to nutrition indicators, between 2000 and 2016, the national stunting prevalence rate declined by 20% from 58% to 38%, whilst underweight declined by 17% from 41% to 24%, and wasting from 12% to 10% [5].

Even though there is an improvement in undernutrition over the years, stunting, wasting and underweight remain to be prevalent and important public health problem in Ethiopia. In an effort to accelerate the reduction of undernutrition, the Government of Ethiopia developed national nutrition strategy and national nutrition programs (NNP). The revised NNP aims to strategically address nutrition problem by focusing on the life cycle approach to map key actions needed to improve the nutritional status of women and children starting with the first thousand days including nutrition actions for in and out of school adolescents [6]. The first 1,000 days represent a window of opportunity from conception through first 2 years after birth [7,8]. It represents a life window where nutritional deficiencies in the first 1000days can exert an overwhelming impacts [9–11].

It has been identified good nutrition and healthy growth during this period have lasting benefit throughout life by helping avert child mortality and lifelong disease burden, maximizing growth, enabling children to reach their cognitive and developmental potential, particularly when combined with psychosocial stimulation [8,9,11–16]. However, it is not enough to know that a nutrition intervention is efficacious; it is also necessary to identify and address barriers and enablers for effective implementation of interventions under large-scale, real-world conditions.

To this effect, Addis Ababa University with the Federal Ministry of Health (FMOH) of Ethiopia and Irish Aid conducted an implementation research to develop four model nutrition districts. The overarching goal of the project was to create four model districts which implement evidence based, high impact and cost-effective package of nutrition interventions through the continuum of care; during delivery and through to infancy and up to 24 months old, from adolescent to pregnancy (motherhood). This study was conducted as part of the implementation research with the aim of assessing structural readiness of health facility and extent of provision of nutrition services at different contact points in four implementation districts.

Materials and methods

Study setting

The present study was part of a broader implementation research by Addis Ababa University in collaboration with the FMOH of Ethiopia and Irish Aid. The aims of the implementation research were; (i) Understanding barriers and facilitators of comprehensive and integrated nutrition service at primary health care unit (PHCU) level;(ii) Developing implementation protocol based on the finding from desk review, rapid assessment and barrier and facilitator analysis; (iii) Piloting the

implementation protocol on selected kebeles; and (iv) Program learning and optimizing the implementation to create model nutrition districts.

This study was conducted with the aim of assessing the overall nutritional services provision in selected districts. It was conducted in four regions of Ethiopia namely Amhara, Oromia, Tigray and Southern Nations, Nationalities and Peoples' Region (SNNPR). The districts included were South Achefer, Kombolcha, Raya Azebo and Offa respectively. The estimated catchment population for these districts were 161,644, 189,945, 159,347 and 135,136 respectively. There were a total of 26 health centers and 84 health posts in the four selected districts. Of which, 23 health centers and 33 health posts were selected randomly for this study. The health centers were staffed by ~~H~~health officers, nurses, pharmacists, laboratory technicians and midwives while the health posts were staffed by health extension workers.

South Achefer ~~D~~district is located in West Gojjam Zone Administration, North-West, Ethiopia. Subsistence agriculture is the main economic activity, and the income of majority of the households depends on agriculture [17] . Kombolcha district is located in Eastern Hararghe Zone, Oromia National Regional State (ONRS)~~-~~ ~~and~~ 542 km east of Addis Ababa.~~ with latitude 42° 07' 0'' E and longitude of 9° 25' 60'' N~~. About 8% of the population professed Ethiopian Orthodox Christianity [18]. Crop-livestock farming activity is the main source of income and employment to the society, where crop production takes the principal part of income of the society [19]. Raya Azebo district is located in ~~at~~ Tigray region ~~and~~ 652 km away from ~~capital~~ Addis Ababa.~~ with an altitude range of 930 to 2300 meters above sea level (masl)~~. Ninety percent of the district is considered "midland" (1500–2300 m) and 10% is considered "lowland" (< 1500 m) [20].

Offa district is one of 16 districts (Woredas) in Wolaita Zone and is located at 383 km south of Addis Ababa on the Sodo Gofa main road. The Offa district, altitude ranges 1200 around river Gogara in the south, 2800 m above sea level in the north. Geographically Offa is located in Latitude of 6° 44′ 59.99" N and Longitude of 37° 29′ 59.99" E [21].

Study design and period

We used a mixed method approach employing a quantitative study followed by a qualitative study. This study was conducted between June-August, 2018.

Sample size and sampling procedure

The sample size for this study was estimated using a sample size formula for a single proportion. The assumptions used to calculate the sample size were; (i) percentage of women who received nutritional counseling about exclusive breast feeding during postnatal care PNC (P) at 50%, (ii) a 95% confidence level and (iii) 5% margin of error. The calculated sample size was 384. We allocated the sample size proportionally across the four districts.

For an in-depth understanding about the overall nutrition service provision, we conducted a total of sixty (60) key informant interviews in four districts. The key informants were; district administration head (*n* = 1), district health office head (*n* = 2), deputy district health office head (*n* = 2), district nutrition focal person (*n* = 4), program officers (*n* = 6), service providers at health center (*n* =24), religious leader (*n* = 1), women development army (WDA) leader (*n* = 1), health extension workers (HEW) (*n* = 9) and PHCU directors (*n* = 10). We employed a purposeful

sampling method to recruit key informants based on experience on nutrition service provision and management.

Data collection tool and procedure

Observation of services

The data collection addressed various dimensions of the comprehensive integrated nutrition services (CINS) provision at different contact points. The first dimension evaluated was the structural readiness. In this dimension, we assessed attributes of context in which care is delivered by observing availability of equipment, guidelines, social behavioral change communication (SBCC) materials and registration and reporting formats. Structural readiness was assessed in a total of 23 health centers and 33 health posts. We also assessed the characteristics of service providers including sex, age, level of education and nutrition training.

The second dimension evaluated was the process of service delivery. In this dimension, we observed the service provision against standard service guideline. This was done through direct observation of service providers while providing nutrition services at difference contact points. We ~~O~~observed contact points including Integrated Management of Neonatal and Childhood Illnesses (IMNCI) unit, immunization unit, antenatal care (ANC) unit~~,~~ and Post-Natal care (PNC) unit. We observed a total of 103 IMNCI, 73 immunization, 94 ANC and 79 PNC service provision. Some of the observed services include anthropometric assessment and interpretation, provision of IFA supplementation, provision of nutrition counselling and essential counselling skill of service providers. We used the CINS guide published by the FMOH to develop observation checklists (S2 Text). We also pretested the tools and made all the necessary modifications ~~were made~~ prior to data collection.

Key informant interviews

Interview guides were developed with open-ended questions to have high degree of flexibility. Interview guides covered topics on multi-sectoral collaboration, integration of nutrition services, planning of nutrition services, registration and reporting of service provision, supportive supervision, human resource and motivation of service providers (S3 Text).

The date, time and place of interview were arranged following what was most favorable and comfortable for the participants. Interviews were conducted in a quite office and privacy was ensured to enable participants to feel free and express their opinions. All interviews were audio taped and took about an hour on average. All data were collected by the project coordinators assigned in the four districts. All data were reviewed for completeness and consistency.

Statistical analysis

Quantitative analysis

We used Epi data version 3.1 for data entry and the statistical software package STATA version 14 for data cleaning and analysis. Descriptive statistics was run on the different components of the nutritional services. Frequency tables and graphs were used to further describe the data set. Differences in service provider's counselling skill across contact points were reported using Pearson's chi-square (X^2) test. Statistical significance was set at $p<0.05$.

Qualitative data analysis

To analyze the qualitative data, we initially developed field note template. Using this template, we wrote an expanded field note for each interview. Then we developed analysis matrix where the rows consist the subgroup selected for the study i.e. the respondents and the columns stands for the topic of study (i.e. the question asked). Using this matrix, we then extracted key findings from the field notes. Quotations were used to further illustrate the responses of the respondents on important issues.

Ethics approval and consent to participate

Ethical clearance was obtained from the Research Ethical Committee of School of Public Health, Addis Ababa University. In addition, permission was obtained from relevant Federal and local government offices. Written informed consent was obtained from the participants after the necessary explanation about the purpose, procedures, benefits and risk of the study have been made. The respondent’s~~s’~~ right to refuse few or all of the questions was respected at all times. In addition, ~~P~~privacy of participants and confidentiality of the information~~ found~~ obtained was kept.

Results

Structural readiness

Availability of essential equipment at health facility. The structural readiness of health centers for nutrition service delivery is presented in table 1. As indicated, the majority of ANC consultancy room (21/23) were equipped with weighing scale, while only (17/23) and (14/23) IMNCI and immunization room were equipped with weighing scale respectively. Height scale was available

in (16/23) IMNCI and ANC consultation room while only (8/23) immunization room were equipped with height scale.

Table 1: Availability of equipment, guidelines and record keeping registers at health centers (n=23).

	Immunization	IMNCI	ANC	Delivery
**Anthropometric equipment**				
Baby weighing scale	18	18	NA	23
Adult weighing scale	14	17	21	14
Height/length board	8	16	16	0
MUAC tape	19	23	19	16
**Counselling guide**				
AMIYCN guideline	10	10	8	8
**Registration book**				
Registration book and reporting format	23	23	23	23

*NA not applicable

The structural readiness of health post for nutrition service delivery is shown in table 2. Most of the health posts had baby weighing scale (32/33), MUAC tape (33/33) and reporting formats (30/33). We also observed a critical shortage of equipment~~’s~~ and guidelines and only few of the health posts had Adolescent, Maternal, Infant and Young Child Nutrition (AMIYCN) guideline (9/33), height board (8/33) and demonstration equipment~~’s~~ (6/33).

Table 2: Availability of equipment, guidelines and record keeping registers at health posts (n=33)

Equipment	Available and functional	Not available
Baby weighing scale	32	1

MUAC tape	33	-
Reporting format	30	3
ANC ~~R~~registration book	27	6
Child card/record	23	10
Adult weighing scale	20	13
SAM guide line	19	14
PNC registration book	15	18
AMIYCN guide line	9	24
Height/length board	8	25
Demonstration equipment	6	27

Table 3 shows availability of medical supplies for nutrition service in health centers and health posts. We observed that most of the health centers and health posts had adequate supply of drugs such as antibiotics, ORS, Iron folic acid, Zinc and Vitamin A. However, supplies such as Albendazole and RUTF ~~'s~~ were not available in nearly quarter of the health centers observed. It is important to note that Daily ration is nearly absent in all health centers and health posts. Only one health center and seven health posts had the daily ration at the time of observation.

Table 3: Availability of drug supply for nutrition services at health centers and health posts.

List of drugs	Health center (n=23)	Health post (n=33)
Antibiotics (Amoxicillin, Cotrimoxazole)	23	33
ORS	23	32
Iron folic acid (60mg elemental iron + 0.4µg folic acid)	23	31
Zinc (20mg)	23	30
Vitamin A (50,000IU)	21	30

Albendazole (400mg)	17	29
RUSF	16	20
RUTF F-75 (75kcal/100ml)	10	NA
RUTF F-100 (100kcal/100ml)	8	NA
Daily rations (TSF)	1	7

*NA not applicable

Nutrition training of health care providers: As shown in table 4, more than half (54.5%) of ANC service providers were clinical nurses/midwives and were predominantly female (80.7%). In addition, 53.1% of service provider in IMNCI were clinical nurses/midwives and were predominantly male (68.7%). Less than half of service providers in ANC (40.4%), PNC (40.4%) and IMNCI (42.2%) received training on nutrition.

Table 4: Characteristics of health care providers delivering ANC, PNC, immunization and IMNCI at health centers and health posts

	ANC (n=57)	PNC (n=52)	Immunization (n=41)	IMNCI (n=64)
**Sex of providers**				
Male	11 (19.3%)	9 (17.3%)	12 (29.3%)	44 (68.7%)
Female	46 (80.7%)	43 (82.7%)	29 (70.7%)	20 (31.3%)
**Provider's Age (mean ±SD)**	26.4 ± 3.68	27.3± 4.26	28.75± 5.05	28.2± 3.35
**Level of education**				
Bsc (Health officer, nurse and midwife	15 (26.3%)	9 (17.3%)	4 (9.7%)	22 (34.4%)
Diploma (Nurse and ~~M~~midwife	31 (54.4%)	30 (57.7%)	18 (43.9%)	34 (53.1%)
HEW	11 (19.3%)	13 (25%)	19 (46.4%)	8 (12.5%)
**Nutrition training**				
Trained	23 (40.4%)	21 (40.4%)	27 (65.8%)	27 (42.2%)

	Not trained	34 (59.6%)	31 (59.6%)	14 (34.2%)	37 (57.8%)

Process evaluation

Anthropometric assessment

We assessed the nutritional service delivery by observing a total of 103 IMNCI, 73 immunizations, 94 ANC and 79 PNC service provisions. Figure 1 shows result from observation of anthropometric assessment at IMCNI service point. We found that, only 62 (60.2%) children had their weight measured, 38 (36.9%) had their height measured and 60 (58.3%) children had their MUAC measured. Of those children whose anthropometric measurement was taken, correct interpretation was conducted for only 32 (51.6%) children using the weight for age z-score and for 28 (73.7%) children using height for age Z-score. Correct interpretation using MUAC cutoff point was conducted for only 39 (65%) children. At immunization service unit, only 12 children had their weight measured while none of the children had their height measured.

We observed a total of 94 ANC and 79 PNC service provision to assess the nutritional service delivery ~~and provision~~ for mothers. ~~.~~ We found that only 75 (79.8%) mothers had their weight measured and 41 (43.6%) had their MUAC measured at the ANC service point ~~provision~~. In addition, only 15 (15.9%) mothers had their height measured. Of those mothers whose MUAC was measured, correct interpretation using MUAC cutoff was conducted for only 34 (82.9%) mothers.

Nutrition counselling provision

Table 5 shows nutrition counselling provided to mothers who came for IMNCI service. Out of 31 mothers with children under six months of age who visited IMNCI, only 19 (61.3%) were counselled on exclusive breastfeeding for the first six months. We also found that counselling on

complementary feeding is suboptimal. For example, of the total number of children illegible for counselling on initiation of complementary feeding, only 13 (42%) mothers were counselled about initiation of complementary food at six months. ~~,~~In ~~only~~addition, only 15 (20.8%) were advised on diversity i.e. to feed a child from animal source foods, and 15 (17.5%) were advised on increasing the frequency of feeding with age. ~~as the child gets older~~.

Table 5: Proportion of mother receiving nutrition counselling at IMNCI service provision point.

**Nutrition~~al~~ counselling during IMNCI service (n=103)**	**Frequency (%)**
**Counselling on breastfeeding**	
Advised on ~~E~~exclusive~~ly~~ breastfeeding ~~BF~~ for the first 6 months (n=31)	19 (61.3%)
Advised on ~~C~~continuing frequent, on-demand breastfeeding until 2 years of age (n=84)	27(32.1%)
**Counselling on complementary feeding**	
Advised on initiation of complementary food~~CF~~ at 6 months (n=31)	13(41.9%)
Advised on diversity to feed a child from grain, roots and tubers (n=72)	25(34.7%)
Advised on diversity to feed a child from legumes and nuts (n=72)	23(31.9%)
Advised on increasing fluid intake during illness, frequent breastfeeding, and encourage the child to eat soft, varied, appetizing, favorite foods (n=103)	26(25.2%)
Advised on diversity to feed a child from fruits and vegetables (n=72)	18(25.0%)
Advised on diversity to feed a child from animal source foods (n=72)	15(20.8%)
Advised on gradually increasing food consistency as the infant gets older (n=72)	16(22.2%)
Advised on the practice responsive feeding (n=103)	18(20.8%)
Advised on increasing the frequency of feeding with age~~as the child gets older~~ (n=72)	15(17.5%)
**Counselling on WASH**	
Advised on personal and ~~F~~food ~~H~~hygiene (hand washing before food preparation, after using toilet, during feeding a child (n=103)	15(14.6%)
Advised on ~~P~~proper storage of food (n=103)	10(9.7%)

Table 6 shows the proportion of mothers receiving nutrition counselling at the immunization service point. We found that, out of 68 mothers with children under six months of age who visited immunization unit, only 11 (16.2%) were counselled to exclusively breastfeed their child for six months and only 9 (13.2%) mothers were counselled about initiation of complementary feeding at 6 months. During the observation at immunization unit, we only had 5 mothers with children ~~who were~~ above the age of six months and no nutritional counselling was given for these~~s~~ mothers.

Table 6: Proportion of mothers receiving nutrition counselling at the immunization service provision point.

**Nutrition~~al~~ counselling during Immunization service (n=73)**	**Frequency (%)**
**Counselling on breastfeeding**	
Advised on ~~E~~exclusive~~ly~~ breastfeeding~~BF~~ for the first 6 months (n=68)	11(16.2%)
Advised on ~~C~~continuing frequent, on-demand breastfeeding until 2 years of age (n=73)	5(6.9%)
**Counselling on complementary feeding**	
Advised on initiation of complementary food ~~CF~~ at 6 months (n=68)	9(13.2%)
Advised on increasing fluid intake during illness, frequent breastfeeding, and encourage the child to eat soft, varied, appetizing, favorite foods (n=73)	4(5.5%)
Advised on the practice responsive feeding (n=73)	3(4.1%)
Advised on increasing the frequency of feeding with age~~as the child gets older~~ (n=5)	
Advised on gradually increasing food consistency as the infant gets older (n=5)	0(0.0%)
Advised on diversity to feed a child from grain, roots and tubers (n=5)	0(0.0%)
Advised on diversity to feed a child from legumes and nuts (n=5)	0(0.0%)
Advised on diversity to feed a child from fruits and vegetables (n=5)	0(0.0%)
Advised on diversity to feed a child from animal source foods (n=5)	0(0.0%)

Counselling on WASH	
Advised on personal and ~~F~~food ~~H~~hygiene (hand washing before food preparation, after using toilet, during feeding of a child (n=73)	4(5.5%)
Advised on ~~P~~proper storage of food (n=73)	1(1.4%)

Table 7 shows various components of nutrition counselling provided to mothers who came for ANC follow-up. We observed sub optimal nutrition counselling service provided to pregnant mothers against the counselling standards set at ANC clinics. A total of 74 (78.7%) mothers were counselled on having one extra meal every day. While 46 (48.9%) mothers were counselled on having variety of food, ~~and~~ 39 (41.5%) were counseled on the importance of having variety of food. Counselling on what to avoid such as eating raw food and what to limit such as caffeine was given for only for 4/94 and 3/94 mothers respectively. We have also observed that only 18 (19.2%) mothers were counselled on maternal health complication due to under nutrition during pregnancy.

Table 7: Proportion of mothers receiving nutritional counselling at antenatal care service provision point.

**Nutritiona~~l~~ counselling (n=94)**	**Frequency (%)**
Advised the mother to have one extra meal/ snack every day	74 (78.7%)
Advised the mother to eat from variety of food groups using locally available food	46 (48.9%)
Advised the mother about the importance of eating variety of food groups	39 (41.5 %)
Advised the mother to have adequate fluid intake (*at least 8 glasses of fluid everyday*)	29 (30.9%)
Advised the mother about fetal complications of under nutrition during pregnancy	20 (21.3%)
Advised the mother about having seasonal fruits and vegetables everyday	19 (20.2%)
Advised the mother about maternal complications of under nutrition	18 (19.2%)

Advised the mother about the importance of exclusive breast-feeding for six month (4th visit) (n=21)	8 (38.1%)
Advised the mother on the importance of early initiation of breast-feeding (4th visit) (n=21)	4 (19%)
Advised the mother to avoid pre-lacteal feeding (4th visit) (n=21)	3 (14.3%)
Advised the mother about adding iodized salt when serving food (after cooking)	13 (13.8%)
Advised the mother on importance of feeding colostrum (4th visit) (n=21)	1 (4.8%)
Advised the mother to expose the child to direct sun light (4th visit) (n=21)	1 (4.8%)
Advised the mother what to avoid during pregnancy *(i.e. Alcohol, raw/uncooked foods and vegetables)*	4 (4.3%)
Advised the mother what to limit during pregnancy (*i.e. caffeine, fat)*	3 (3.2%)
**Counselling on WASH (n=94)**	
Advised the mother to keep personal hygiene	46 (48.9%)
Advised the mother to wash hand after toilet use, and before meal preparation	11 (11.7%)
Advised the mother to keep utensils and cooking material clean	9 (9.6%)
Advised the mother to use treated water for drinking	3 (3.2%)
**Counselling on ITN (n=94)**	
Advised on ITN use	34 (36.2%)

Figure 2 shows counselling provided to mother on IFA during ANC follow up. We found that majority of the mothers 85 (90.4%) who came for ANC were provided IFA prescription. However, of those who were given prescription, only 73 (85.9%) were counselled to take IFA daily for six months. In addition, only 49 (57.7%) mothers were counselled about the benefit of IFA and 36 (42.4%) were counselled on the possible side effect of IFA.

Table 8 shows various components of nutrition counselling provided to mothers who came for PNC service. We have observed 75 (94.9%) mothers were counselled to exclusively breastfeed up

to six months. Regarding maternal nutrition, 63 (79.8%) mothers were counseled to have two extra meal and rest during lactation. Counselling about maternal complication of undernutrition was given for only 27 (34.2%) mothers.

Table 8: Proportion of mother receiving nutritional counselling at postnatal care service provision point.

**Nutritional counselling (n=79)**	**Frequency (%)**
Advised mother to exclusively breastfeed up to six month	75 (94.9%)
Advised the mother about extra meal and rest during lactation	63 (79.8%)
Advised mother to breast-feed on demand	62 (78.5%)
Advised the mother on the importance of exclusive breast-feeding for six month	56 (70.9%)
Advised mother on correct positioning and attachment during breast-feeding	54 (68.4%)
Advised the mother to eat from variety of food groups (locally available food)	48 (60.8%)
Advised the mother to eat food rich in iron	42 (53.2%)
Advised the mother to eat a diet that is rich in protein and fluid	38 (48.1%)
Advised the mother on the importance of eating variety of food groups	37 (46.8%)
Advised mother to avoid all dietary restriction if any	35 (44.3%)
Advised the mother about maternal complications of under nutrition during lactation	27 (34.2%)
Advised the mother to have adequate fluid intake (at least 8 glasses everyday)	21 (26.6%)
Advised the mother to have seasonal fruits and vegetables everyday	14 (17.7%)
Advised the mother about adding iodized salt when serving food (after cooking)	13 (16.5%)
**Counselling on WASH (n=79)**	
Advised the mother to use treated water for drinking	15 (18.9%)
Advised the mother about safe disposal of potentially infectious soiled pads or other materials	14 (17.7%)
Advised the mother to wash hand after toilet use, and before meal preparation, after cleaning baby, before feeding ,before breast feeding	12 (15.2%)
Advised the mother to keep utensils and cooking material clean	15 (18.9%)
Advised the mother to keep personal hygiene	40 (50.6%)

Counselling skill of service providers

Table 9 shows the counselling skill of service providers working at IMNCI and immunization service points. Listening to what the clients have to say was higher among service providers at IMNCI 91.2% compared to service providers at immunization 75.3% *(P<0.05)*. Similarly, recognizing and praising what clients were doing correctly was higher among ~~at~~ service providers at IMNCI 23.3% compared to service providers at immunization 9.6% *(P<0.05)*. During counselling, providers identified key difficulties for only 20.4% and 6.8% of the clients at IMNCI and immunization respectively *(P<0.05)*.

Table 9: Counselling skill of service providers during provision of counseling at IMNCI and immunization service points.

**Counselling**	**IMNCI (n=103)**	**Immunization (n=73)**	**Chi2 P-value**
Service provider listens to what the client has to say	94 (91.3%)	55 (75.3%)	**0.004**
Service provider recorded history of the client	87 (84.5%)	51 (69.9%)	**0.020**
Service provider used responses and gestures that show interest	84 (81.6%)	54 (73.9%)	0.228
Service provider avoided using judging words	84 (81.6%)	52 (71.2%)	0.107
Service provider Identified key difficulties (if any) and selects with the client the most important one to work on	21 (20.4%)	5 (6.8%)	**0.013**
Of those mothers with key difficulties service provider discussed options on key difficulties the client raised	17 (80.9%)*	5 (100%)*	0.056
Service provider recommends and negotiates do-able actions to help the client select the best	16 (76.2%)*	5 (100%)*	0.080

option to try depending on her context and resources			
Service provider helped the client agree to try one of the options and asks them to repeat the agreed-upon do-able action	15 (71.4%)*	5 (100%)*	0.112
Service provider let the client talk through her concerns before correcting information	59 (57.3%)	31 (42.5%)	0.053
Service provider recognized and praised what the client is doing correctly	24 (23.3%)	7 (9.6%)	**0.019**

* The denominator is the number of clients identified with key difficulties

Table 10 shows the counselling skill of service providers working at ANC and PNC service points. Listening to what the clients have to say was higher among service providers at ANC 97.9% compared to service providers at PNC 89.9% *(P<0.05)*. Similarly, identifying key difficulties the clients have was higher among service providers at ANC 40.4% compared to service providers at PNC 16.5% *(P<0.001)*. However, discussing about options for the identified difficulties was higher among service providers at PNC 92.3% compared to ~~among~~ service providers at ANC 73.7% *(P<0.05)*. Similarly, recommending doable action for the identified problem was higher among service providers at PNC 76.9% compared to service providers at ANC 68.4% *(P<0.05)*.

Table 10: Counselling skill of service providers during provision of counseling at ANC and PNC service points.

**Counselling**	**ANC (n= 94)**	**PNC (n=79)**	**Chi2 P-value**
Service provider listens to what the client has to say	92 (97.9 %)	71 (89.9%)	**0.045**
Service provider used responses and gestures that show interest	89 (94.7%)	71 (89.9%)	0.232
Service provider let the client talk through her concerns before correcting information	84 (89.4%)	63 (79.8%)	0.078

Service provider avoided using judging words	83 (88.3%)	63 (79.8%)	0.123
Service provider Identified key difficulties (if any) and selects with the client the most important one to work on	38 (40.4%)	13 (16.5%)	**0.001**
Of those mothers with key difficulties service provider discussed options on key difficulties the client raised	28 (73.7%)*	12 (92.3%)*	**0.023**
Service provider recommends and negotiates do-able actions to help the client select the best option to try depending on her context and resources	26 (68.4%)*	10 (76.9) *	**0.015**
Service provider helped the client agree to try one of the options and asks them to repeat the agreed-upon do-able action	26 (68.4%)*	9 (75%)*	0.491
Service provider recognized and praised what the client is doing correctly	43 (45.7%)	25 (31.7%)	0.059
Service providers recorded history of the client	71 (70.9%)	56 (70.9%)	0.083

* The denominator is the number of clients identified with key difficulties

Qualitative Result

A total of 60 key informants participated in this study. The participants include district administrative head, district health office head, ~~deputy~~ district health office deputy head, district nutrition focal person, program officers, service providers at health center, religious leader, WDA, HEW and PHCU heads.

Multi-sectoral collaboration

We found that the Woreda nutrition coordination body (WNCB), Woreda nutrition technical committee (WNTC) and Kebele nutrition technical committee (KNTC) are in place in the four districts. These structures were established following the 1000 days plus program launching. Key informant interview indicated that the issue is not related to the presence of the multi-sectoral

coordination bodies rather ~~and~~ it is related to its functionality. It is important to note that neither the coordination body nor the technical committee meet regularly and are productive enough. One of the key informants explained about the functionality of WNCB, WNTC and KNTC as follows:

> *" The WNCB meetings are not conducted regularly. There is usually third body request to have the meeting. It is not scheduled ahead and usually done hastily. There is unequal commitment of sectors, not planning together and there are no terms of reference. I can say WNTC almost does not exist and needs reestablishment. Regarding KNTC; the HEWs have capacity gap, they are not committed and motivated because of carrier structure problem, inadequate salary, no recognizing/rewarding mechanism, work overload etc. The kebele leaders are not committed as well."*

The functionality of the structure is mainly dependent up on the existence of nutrition projects in the district. ~~.~~All key informants believe that having WNCB is very important for the success of nutrition services. However, there is lack of ownership among different members. One participant explained the issue of ownership as follows:

> *"There is a tendency of pushing this job aside by the health sector due to different emerging activities and other priorities. In addition, the other sectors are dependent on an external initiation and they don't own this structure as part of their routine job. Apart from this there is also lack of logistic to conduct the meeting effectively…. you now like incentives."*

Another important bottleneck related to the functionality of the WNCB is ~~related to~~ the absence of shared definitive roles ~~s~~and responsibilities among the members. The participants of this study indicated that the members in the coordination body externalize their responsibility to the health sector and there is an assumption that nutrition is the concern of health sector only.

One of the participant expressed the following:

"Because of other political priorities the coordination body is not operating to the envisioned extent. So mainly, it is the technical committee that is working better currently. However, there are certain still limitation on the technical committee as well. There is a high challenge with ownership of the body as they feel this task is solely the responsibility of the health sector".

Integration of nutrition services

Our assessment shows that there is sub optimal and poor integration of nutrition services with in child and maternal health services at the health centers and health posts. We documented a physical rather than functional integration of nutrition services. For instance, growth monitoring and promotion (GMP), immunization and IMNCI service are given in one room usually by one service provider. However, there is missed opportunity of providing GMP for children who came for immunization and IMNCI service.

"There is no separate room for GMP, IMNCI and immunization at health center level. These three activities are offered in a room by a provider. The same is true for ANC, Delivery and PNC …. However; service providers are not using this opportunity to give nutrition service in an integrated manner."

Human resource, training and motivation of service providers

We have assessed staff adequacy, training and motivation of service providers at different levels in the districts. According to our finding, there is critical staff shortage at each level from district health office to the health post. This staff shortage was more profound at remote health facilities.

" I myself focal for three positions, work in regular and emergency outpatient department (OPD), the health center head also works in OPDs, other staffs also have burden and

generally there is staff shortage; there has been very critical shortage though yesterday one health officer joined the health center. There are only 5 staffs including the head and me. There is shortage of staffs in all departments."

Even though there is shortage at health centers, it was more significant at health posts~~ level compared to health centers~~. The number of the HEWs was not comparable with the number of households living in their catchment area. One participant explained:

"Based on the standard one health extension worker gives service to 2500 populations in her catchment area (1:2500). But the ratio in our woreda is 1: 10,000 to 11,000 populations. This is similar in the health center as well, there is not enough service provider compared to service seekers."

Besides staff shortage, we documented high staff turnover. Similar to staff shortage, the turnover was more prominent in rural health facilities. The turnover was most common among midwives, health officers and HEW.

"This year 8 HEWs left their job and moved to different cities by changing their job. It became a big challenge for the woreda. Some health posts have only one HEW. Currently Zemo health post have no HEW as a result it is closed."

We have identified training gaps in AMIYCN, out-patient therapeutic program (OTP), community-based nutrition (CBN), management of sever acute malnutrition, blended integrated nutrition and counselling skill. Besides, the training opportunities mainly depend on the partner's or training provider organization's objective rather than the need of the health sectors in the district. Furthermore, we have identified poor opportunities for health care providers to further their education and future career development.

" …there is no study opportunity. As I mentioned for you earlier, I joined this health center nearly five years back. It is very difficult to talk on this and there is no chance to pursue our education. Even when there are opportunities, there is no fair selection of candidates."

We also evaluated motivation of service provider. We have found poor motivation among service providers. Participants described variety of factors affecting their motivation including access to training, access to further education, timely duty payment, work overload, and inadequate salary.

Planning of nutrition services

The five-year strategic health sector plan is used as a baseline for district health office~~'s~~ planning purpose. During planning HEWs, health center heads, personnel from district health office, ~~District health office personnel~~ and ~~other~~ delegates from other sectors such as children and women affairs, agriculture, finance etc. are ~~will~~ usually ~~be~~ involved. However, the planning followed top to bottom approach. This approach does not take in to account the existing situation on the ground. Because of this, service providers had to deal with numbers sent from the top-level officials that doesn't really exist in their catchment area. They have explained this as follows:

"We are facing challenge during planning as we breakdown the figures we took from the woreda to each health post by using conversion factors. Therefore, the plan is not based on the existing situation of the PHCU."

"There was a time where they give us 5000 plans while we have 3000 in our catchment and vice versa so don't get surprised when you see 205% achievement."

Registration and reporting of nutrition services

We observed multiple registration books being used by professionals for different services. Registration format were not available at facilities according to the standard set by the FMOH. We documented ~~a~~ shortage of registration books especially at health post level.

> *"We are using self-made registry. This is a burden for us since making the register is tiresome. It also has drawback as we might omit some important variables".*

Another challenge with registration book was related with portability. Health extension workers used a plain paper to document activities during outreach services because it is difficult to carry the registration books to outreach sites. This has drawback as the HEWs often forget to copy to the registers resulting in poor documentation and inconsistency among reports and registers. In general, there is poor documentation especially at health post level.

> *"There is no documentation problem at health center level. We perform activities and record accordingly......... but there is a gap at health post level. Last time the health center head and I along with kebele leader came across poor documentation. This might be because the HEW are newly assigned since senior HEWs have left their job for different reasons."*

Similar to registration, we have identified challenges related with reporting as well. The challenges were lack of timeliness, reliability, and completeness of the reports.

> *"There was an incident where one of the health extension worker sent 0 ANC on the HMIS report and 20 ANC on the disease report, so we will cross check for such kind of errors and give feedback on how to improve the report in the future".*

Supportive supervision

The district health office provides supportive supervision to the health centers and health centers provide supportive supervision to health posts. There were two forms of supportive supervision in the district; integrated supportive supervision and program specific supportive supervision. As the name implies, integrated supportive supervision was conducted every quarter by integrating different case team leaders from the district health office. The program specific supervision was conducted whenever the need arises by individuals assigned to a specific program. This type of supervision focuses on specific programs.

> *"There is Integrated Supportive Supervision (ISS) this will be done every quarter where different officers from the Woreda health office supervise health centers, health posts and at least 10 sample households at community level."*

Discussion

The landscape analysis was intended to assess service readiness and nutrition service provision at different service provision points. The result from this study indicated poor structural readiness and gap in nutrition service~~s~~ provisions. Health facilities lacked essential anthropometric instruments, nutrition guidelines, registration and reporting formats. Poor structural readiness was more prominent at health posts ~~levels~~ compared to health centers. Our process evaluation indicated that there is missed opportunity of anthropometric assessment and preventive nutrition counselling at different contact points. Such problem was more critical at immunization unit than other units. In our qualitative assessment, we found that there is an existing but non-functional Woreda nutrition coordination body and technical committee. Training gaps, staff shortage and high staff

turnover were the main challenges faced by service providers resulting in burden, fatigue and poor motivation ~~among providers~~.

We have observed poor structural readiness in both health centers and health posts. However, overall facility preparedness was better in health centers compared to health posts. This might be because the health centers provide comprehensive primary health care including promotive, curative and rehabilitative services as compared to health posts. Even though health centers exhibited better structural readiness, the level of readiness varied across different units. For instance, there were greater availability of weighing scale at ANC units than other units. Such type of problems were~~are~~ also common in other countries like Bangladesh where ANC rooms have better structural readiness than other units [22]. This might be because ANC has been practiced well as part of the health service implementation compared to the recently introduced nutrition service implementation in IMNCI [22].

Integration of nutrition service is expected at different contact points such as ~~with~~ IMNCI. It is assumed that if screening is performed routinely in curative clinics, it creates a better opportunity for timely detection and intervention. However, our assessment of service provision at~~in~~ IMNCI indicated a missed opportunity for basic nutrition screening and counselling. Indeed, of the total number of children who came for IMNCI service, only 60.2% had their weight measured. It is important to note that the current IMCNI algorithm asks for weight of the child and yet 40% of the children were not weighed. Similarly, a study done in Botswana showed limited nutrition and dietary screening in curative clinics [23]. This missed opportunity could be attributed to lack of anthropometric equipment, poor motivation of staffs, shortage of staff and lack of supervision.

Poor interpretation of anthropometric assessments were also the findings from this study. Without nutrition assessment and correct interpretation, it is unlikely there would be appropriate management of undernutrition and appropriate treatment of other childhood illness [24]. Of those children whose weight was measured at IMNCI unit, correct interpretation using ~~of~~ weight for age Z-score was done for only 51.6% of the children. This might be related with capacity gaps, poor commitment and workload among service providers.

The other challenge that we identified was related to provision of preventive services such as proper counselling during clinical contact. Poor nutrition counselling could be a result of high caseload, staff shortage and capacity gap which led to prioritization of the immediate clinical concern rather than focusing on preventive intervention. However, overall nutrition service at IMNCI was relatively better as compared to immunization unit. We have identified a critical missed opportunity at immunization unit where only 16.4% of children had their weight measured while none of the children had their height measured. Lack of essential anthropometric instruments such as weight scale and stadiometer could have been possible reason for lack of appropriate nutritional assessment. In addition to ~~Besides,~~ lack of essential equipment, high caseload during immunization day could also be the possible reason for missed opportunity. The trend of provision of immunization was using a fixed day resulting in high caseload making immunization one of the most rushed services. This has resulted in poor nutrition counseling service at immunization unit. Of all the services, the least preventive counselling service was observed in this unit.

Immunization services are often widely available and potentially can support and be supported by additional health interventions. The combined delivery or integration of linked health interventions including GMP, nutrition advises and preventive cares for a more effective way of achieving common health goals [25]. In the study area, there was an opportunity to integrate nutrition

services as there was a physical integration of IMNCI, Immunization and GMP services. These services were given in a single room and by a single provider in most of the health facilities. However, the service provision did not progress beyond the physical integration which resulted in missed opportunities.

The level of provision of essential nutrition services was better at ANC and PNC unit as compared to IMNCI and immunization unit. This is reflected by improved nutrition counselling at ANC and PNC unit. Nutritional assessment was also better at ANC unit. For instance, majority (79.79%) of the mothers at ANC were weighed. We have also observed a relatively better counselling at ANC unit. This improved nutrition service might be due to the preventive nature of ANC service. Since ANC service is preventive than curative it makes it conducive to provide nutrition counselling than other services.

Our study showed prescription of IFA was high however; counselling related to IFA was poor. This finding was similar with a report conducted with the aim of describing and comparing nutrition intervention using service readiness and delivery across different countries. The finding from the study indicated prescription/provision of IFA was higher than counselling service related with IFA. Prescription ranged from 62.2% in Nepal to 87.5% in Senegal. Nonetheless, counselling on benefit of IFA ranged from 18% in Nepal to 72.6% in Tanzania [26]. Counselling on possible side effect was very low ranging from 1.8% in Senegal to 14.2% in Tanzania [26]. Inadequate tools and skill in counselling to support and monitor adherence could be possible reason for the observed result [27]. Poor counselling service about benefit, possible side effect, severity and magnitude of the problem has been identified as barriers for non-adherence of IFA [26,28–30]. A systematic review has identified fear of side effect as the commonest reason for non-adherence which was 46.4% [31]. As a result, counselling addressing benefit and possible side effect with

mitigation strategy to overcome the side effect should be incorporated other than mere provision of the supplement to ensure adherence.

During our assessment, we have identified major challenges faced by service providers. Training gap, staff shortage and high staff turnover were the commonly identified challenges. The training gap was not uniform in all unit; it was more pronounced among~~for~~ health care providers at maternity unit. The other challenge with training is that it was not need based rather it mainly ~~and mainly~~ depend on the objective of the organization providing the training . Similar to training, staff shortage and turnover was also not uniform. It was more common at rural health facilities. This might be due personal reasons and service provider's desire to live in town or near to town. Such problems could be possible reasons for poor implementation of services at different unit.

Motivation of staff was ~~was also found to be~~ one factor for effective delivery of nutrition services. Health extension workers are overloaded with health and other non-health related commitments such as political tasks. This ~~which~~ has resulted in poor motivation and fatigue among ~~of~~ health extension workers. Apart from the burden of work, lack of access to ~~for~~ trainings, lack of timely duty payment, lack of advancement in national career structure and inadequate salary were factors for poor motivation of service providers.

Because of the multi-sectoral nature of nutrition, various stakeholders outside the health needs to be involved, coordinated and linked to gain the synergistic impact for achievement of nutrition interventions [6]. Even though nutrition coordinating bodies and technical committees were established in all the districts, it is not functioning to the desired level. The primary reason for this could be engagement of different sectors in other urgent and political commitments. Even though the sectors will be housed in health sector all member of the coordinating body and technical

committee should be equally accountable [32]. However, this was not the reality in all districts. There was poor ownership and accountability among sectors reflected by externalizing every responsibility to the health sector. This might be a result of lack of strong monitoring and evaluation system in place. In addition, lack of coordination from the federal and regional governments has been identified as a constraint to improve nutrition service delivery in Ethiopia [33]. In order to ensure functionality of coordination body and technical committee, there should be a mechanism to capture data and triangulate from all relevant sectors.

We believe the current study has limitation that needs to be acknowledged. Since we used direct observation, it is difficult to rule out desirability bias. Direct observation during service provision may have encouraged service providers to offer a more thorough service for their clients. However, we believe the impact is minimal since our finding on both the structural readiness and process evaluation were low. The strength of this study was the assessment of both health centers and health posts and the use of multiple methods of data collection techniques including observation, and key informant interviews to assess the structural readiness and provision of nutrition services.

Conclusion and Recommendation

We documented poor structural readiness and gaps to deliver integrated nutrition services in all four districts. There were significant missed opportunities for different nutrition services including nutrition screening and counselling. Poor commitment and accountability of coordination body and technical committee towards nutrition intervention was common. Improving logistics and training may improve delivery of nutrition service. Improving health human resource might reduce missed opportunity that resulted because of high caseload.~~as a result of high case load~~. It might also ensure adequate time to provide thorough preventive counselling service at all units. Ensuring

a clear structure of accountability along with reporting mechanisms in the existing system might ensure functionality and accountability of the coordinating body and technical committee.

Supporting information

S1 Dataset. The minimal data set of this study

S2 Text. Observation checklist used in this study.

S3 Text. Key informant interview guide used in this study.

Acknowledgment

We are very grateful for the financial support of Irish Aid Ethiopia and ~~T~~the Federal Ministry of Health of Ethiopia. We would like to thank Addis Ababa University, School of Public Health for facilitating the conduct of the research. We appreciate the managers and health care providers for their assistance and cooperation during the assessment. Finally, we would like to thank all study participants who took part in this study.

Reference

1. Black RE, Allen LH, Bhutta ZA, Caulfield LE, de Onis M, Ezzati M, et al. Maternal and child undernutrition: global and regional exposures and health consequences. Lancet. 2008;371(9608):243–60.
2. Black RE, Victora CG, Walker SP, Bhutta ZA, Christian P, De Onis M, et al. Maternal and child undernutrition and overweight in low-income and middle-income countries. Lancet. 2013;382(9890):427–51.

3. World Health Organization. Nutrition in the WHO African Region.

4. UNICEF, WHO World Bank Group and United, Levels and Trends in Child Mortality Report, 2017.

5. Ethiopia Demographic and Health Survey 2016 Central Statistical Agency Addis Ababa, Ethiopia ICF International Calverton, Maryland, USA, 2017.

6. Federal Democratic Republic of Ethiopia - National Nutrition Program, 2016-2020.

7. Bhatia J, Bhutta ZA, Kalhan SC, editors. Maternal and child nutrition: the first 1,000 days. Karger Medical and Scientific Publishers; 2013.

8. RE Black, J Hopkins Bloomberg The lancet series on maternal and child nutrition, executive summary, Lancet 372, 1-10, 2008.

9. Karakochuck CD, Whitfield KC, Green TJ, Kraemer K. The biology of the first 1,000 days. CRC Press; 2017 Sep 22.

10. Schwarzenberg SJ, Georgieff MK. Advocacy for improving nutrition in the first 1000 days to support childhood development and adult health. Pediatrics. 2018;141(2).

11. Beluska-Turkan K, Korczak R, Hartell B, Moskal K, Maukonen J, Alexander DE, et al. Nutritional gaps and supplementation in the first 1000 days. Nutrients. 2019;11(12):1–50.

12. Bellieni C V. The Golden 1,000 Days. J Gen Pract. 2016;04(02):2–5.

13. Maalouf-Manasseh Z, Oot L, Sethuraman K. Food and Nutrition Technical Assistance III Project Giving Children the Best Start in Life: Integrating Nutrition and Early Childhood Development Programming within the First 1,000 Days. SecurenutritionOrg [Internet]. 2016;(January).

14. Lowensohn RI, Stadler DD, Naze C. Current concepts of maternal nutrition. Obstetrical & gynecological survey. Pediatr Emerg Care. 2017;33(12):792–3.

15. Wu G, Bazer F, Cudd T, Meininger C. Recent Advances in Nutritional Sciences-Maternal Nutrition and Fetal Development. Nutr [Internet]. 2004;(13):2169–72.

16. Abu-Saad K, Fraser D. Maternal nutrition and birth outcomes. Epidemiol Rev. 2010;32(1):5–25.

17. Sibhatu KT, Qaim M. Rural food security, subsistence agriculture, and seasonality. PLoS One. 2017;12(10):1–15.

18. CSA (Central Statistical Agency) (2006): Ethiopia Demographic and Health Survey 2005. Addis Ababa, Ethiopia. (2008): Statistical Abstract 2007. Addis Ababa, Ethiopia.

19. BOARD (Bureau of Agriculture and Rural Development) (2009): Compiled Report for Kombolcha. Eastern Hararghe Zone, Oromiya Regional State, Ethiopia.

20. Tesfaye G, Gebresamuel G, Gebretsadik A, Gebrelibanos A, Gebremeskel Y, Hagos T. Participatory rural appraisal report: Raya-Azebo Woreda, Tigray region; 2014.

21. Offa Woreda Burau of Agriculture and Rural Development, 2016.

22. Billah SM, Saha KK, Khan ANS, Chowdhury AH, Garnett SP, Arifeen S El, et al. Quality of nutrition services in primary health care facilities: Implications for integrating nutrition into the health system in Bangladesh. PLoS One. 2017;12(5):1–16.

23. Nnyepi MS. Dietary and Nutrition Screening for Children Seeking curative care in health facilities in Botswana. African Journal of Food, Agriculture, Nutrition and Development. 2006;6(2).

24. Arifeen SE, Bryce J, Gouws E, Baqui AH, Black RE, Hoque DME, et al. Quality of care for under-fives in first-level health facilities in one district of Bangladesh. Bull World Health Organ. 2005;83(4):260–7.

25. World Health Orgnization. GIVS: Global Immunization Vision and Strategy 2006-2015.

World Health Organization;2005.
26. Wang W, Benedict RK, Mallick L. The Role of Health Facilities in Supporting Adherence to Iron-Folic Acid Supplementation During Pregnancy: A Case Study Using DHS and SPA Data in Haiti and Malawi. DHS Working Paper No.160. Rockville, Maryland, USA: ICF. 2019;(August).
27. Siekmans K, Roche M, Kung'u JK, Desrochers RE, De-Regil LM. Barriers and enablers for iron folic acid (IFA) supplementation in pregnant women. Matern Child Nutr. 2018;14(July 2017):1–13.
28. Taye B, Abeje G, Mekonen A. Factors associated with compliance of prenatal iron folate supplementation among women in Mecha district, Western Amhara: A cross-sectional study. Pan Afr Med J. 2015;20:1–7.
29. Assefa H, Abebe SM, Sisay M. Magnitude and factors associated with adherence to Iron and folic acid supplementation among pregnant women in Aykel town, Northwest Ethiopia. BMC Pregnancy Childbirth. 2019;19(1):1–8.
30. Arega Sadore A, Abebe Gebretsadik L, Aman Hussen M. Compliance with iron-folate supplement and associated factors among antenatal care attendant mothers in Misha District, South Ethiopia: Community based cross-sectional study. J Environ Public Health. 2015;2015:1–7.
31. Desta M, Kassie B, Chanie H, Mulugeta H, Yirga T, Temesgen H, et al. Adherence of iron and folic acid supplementation and determinants among pregnant women in Ethiopia: A systematic review and meta-analysis. Reprod Health. 2019;16(1):1-14.
32. Government of the Federal Democratic Republic Of Ethiopia Program Implementation Manual of National Nutrition Program (NNP) - I July 2008 – June 2013 Federal Ministry

of Health. 2013;(June).

33. Sustainability S, Nutrition OF, Assessment IN, Program S. Programming and Integrating Nutrition Assessment ,2018.
