# Supplementary material for: Landscape analysis of nutrition services at Primary Health Care Units (PHCUs) in four districts of Ethiopia
Source: PLoS One. 2020 Dec 3;15(12):e0243240. doi: 10.1371/journal.pone.0243240 (PMC7714176; doi:10.1371/journal.pone.0243240)
Supplement: Attachment — Submitted filename: Manuscript - 2020-08-17T154229.001_With comments.docx [file pone.0243240.s004.docx]

**Landscape analysis of nutrition services in primary health care unit, Ethiopia.**

Esete Habtemariam Fenta^1*^, Bilal Shikur Endris^1^, Yalemwork Getenet Mengistu^1^, Fekadu Elias Sadamo^2^, Endashaw Hailu Gelan^1^, Tsegaye Gebrezgher Beyene^1^, Seifu Hagos Gebreyesus^1^

^1^ Department of Nutrition and Dietetics, School of Public Health, College of Health Sciences, Addis Ababa University.

^2^ Department of Reproductive Health and Nutrition, School of Public Health, College of Health Sciences, Wolaita Sodo University

^*^ [esete.f@gmail.com](mailto:esete.f@gmail.com) (EHF)

# **Abstract**

**Background:** Although good nutrition and healthy growth during the first 1000days have lasting benefit throughout life, the structural readiness of health facilities and nutrition services provision during the first 1000 days in Ethiopia is not well understood. The present study was part of a broader implementation research aimed at developing model nutrition services by implementing evidence based, high impact and cost-effective package of nutrition interventions through the continuum of care. This study was aimed at assessing structural readiness of health facilities and the extent of nutrition service provision in the implementation districts.

**Methods:** This assessment was conducted in four districts of Ethiopia. We used mixed method; a quantitative study followed by qualitative study. The quantitative study addressed two-dimensions, structural readiness and process of nutrition service delivery. The first dimension assessed attributes of context in which care is delivered by observing availability of essential logistics. The second dimension assessed what is been done while giving service by direct observation of provision of care at different unit of health facilities. A total of 380 observations were conducted from 23 health center and 33 health posts. The observations were conducted at the Integrated Management of Neonatal and Childhood Illnesses unit, immunization unit, Antenatal care unit, Delivery and Postnatal care unit. Moreover, a total of sixty key informant interviews were conducted with key stakeholders.

**Result:** We have assessed structural readiness of 56 health facilities in four districts of Ethiopia. Both quantitative and qualitative findings revealed poor structural readiness and gap in nutrition services provision. Health facilities lack essential logistics which was found to be more prominent at health post level compared to health centers The process evaluation showed a critical missed opportunity for anthropometric assessment and preventive nutrition counselling at different contact points. This was particularly prominent at immunization unit (where only 16.4% of children had their weight measured and only 16.2% of children under six month of age were counselled about exclusive breast feeding). On maternal services, 90.4% of pregnant women who came for antenatal care were prescribed iron and folic acid. However, only 57.7% were counselled about the benefit and 42.4% were counselled about the side effect. The qualitative findings indicated non functionality of the existing district nutrition coordination body and technical committees, training gaps, staff shortage, high staff turnover resulting in burden, fatigue and poor motivation among service providers.

**Conclusion:** There was poor structural readiness and gaps in delivering integrated nutrition services with a considerable missed opportunity in nutrition screening and counselling.

**Key words:** 1000days nutrition, Landscape analysis, Nutrition service evaluation

# **Background**

Maternal and child undernutrition remain prevalent with important consequence in low-income and middle-income countries [1,2]. According to the 2018 World Health Organization (WHO) African region data, between 2000 and 2015 nine countries have prevalence rate of maternal underweight above 15% and exceeds 20% in Ethiopia [3]. Under-nutrition is responsible for 45% of all under ﬁve child deaths, representing more than 3 million deaths per year [1]. Ethiopia has recorded remarkable improvements in health status and human centered development over the past two decades. Under five mortality rate decreased from 203 per 1000 live birth in 1990 to 58 per 1000 live birth in 2016 [4]. With respect to nutrition indicators, between 2000 and 2016, the national stunting prevalence rate declined by 20% from 58% to 38%, whilst underweight declined by 17% from 41% to 24%, and wasting from 12% to 10% [5].

Even though there is an improvement over the years, stunting, wasting and underweight remain to be prevalent in Ethiopia. In an effort to accelerate the reduction of undernutrition, the Government of Ethiopia developed national nutrition strategy and national nutrition programs (NNP). The revised NNP aims to strategically address nutrition problem by focusing on the life cycle approach to map key actions needed to improve the nutritional status of women and children starting with the ﬁrst thousand days including nutrition actions for in and out of school adolescents [6]. The first 1,000 days represent a window of opportunity from conception through first 2 years after birth [7,8]. It represents a life window where nutritional deficiencies in the first 1000days can exert a devastating impacts [9–11].

It has been identified good nutrition and healthy growth during this period have lasting benefit throughout life by helping avert child mortality and lifelong disease burden, maximizing growth, enabling children to reach their cognitive and developmental potential, particularly when combined with psychosocial stimulation [8,9,11–16]. However, it is not enough to know that a nutrition intervention is efficacious; it is also necessary to identify and address barriers and enablers for effective implementation of interventions under large-scale, real-world conditions.

To this effect Addis Ababa University with Ministry of Health (MOH) of Ethiopia and Irish Aid conducted an implementation research to develop four model nutrition districts. The overarching goal of the project was to create four model districts which implement evidence based, high impact and cost-effective package of nutrition interventions through the continuum of care; during delivery and through to infancy and up to 24 months old, from adolescence to pregnancy (motherhood). This study was conducted as part of the implementation research with the aim of assessing structural readiness of health facility and extent of provision of nutrition services at different contact points in four implementation districts.

# **Materials and methods**

**Study setting**

The present study was part of a broader implementation research by Addis Ababa University in collaboration with MOH of Ethiopia and Irish Aid. The aim of the implementation research was I) Understanding barriers and facilitators of comprehensive and integrated nutrition service at primary health care unit (PHCU) level; II) Developing implementation protocol based on the finding from desk review, rapid assessment and barrier and facilitator analysis; III) Piloting the implementation protocol on selected kebeles; and iv) Program learning and optimizing the implementation to create model nutrition districts.

This study was conducted with the aim of assessing the overall nutritional services provision in selected districts. It was conducted in four regions of Ethiopia namely Amhara, Oromia, Tigray and Southern Nations, Nationalities and Peoples’ Region (SNNPR). The districts included were South Achefer, Kombolcha, Raya Azebo and Offa respectively. The estimated catchment population for these districts were 161,644, 189,945, 159,347 and 135,136 respectively. There were a total of 26 health centers and 84 health posts in the four selected districts. Of which, 23 health centers and 33 health posts were selected randomly for this study. The health centers were staffed by Health officer, nurses, pharmacist, laboratory technicians and midwives while the health posts were staffed by health extension workers.

**Study design and period**

We used a mixed method approach; A quantitative study followed by a qualitative study was conducted from June-August, 2018.

**Sample size calculation**

We used single proportion formula with the assumption of Percentage of women who received nutritional counseling about exclusive breast feeding during postnatal care PNC (P) 50%, 95% level of confidence and 5% margin of error, yielding a total sample size of 384. We then allocated the total sample size proportionally across the four districts.

In order to have a deeper understanding about the overall nutrition service provision, we have conducted a total of sixty key informant interviews in 4 districts. Interview respondents were district administrative head (*n* = 1), district health office head (*n* = 2), deputy district health office head (*n* = 2), district nutrition focal person (*n* = 4), program officers (*n* = 6), service providers at health center (*n* =24), religious leader (*n* = 1), women development army (WDA) leader (*n* = 1), health extension workers (HEW) (*n* = 9) and PHCU directors (*n* = 10). We recruited interviewees with an experience on nutrition purposefully.

**Data collection tool and procedure**

The observation study addressed different dimensions of comprehensive integrated nutrition service (CINS) provision at different contact points. The first dimension was structural readiness. In this dimension we assessed attributes of context in which care is delivered by observing availability of equipment, guidelines, social behavioral change communication (SBCC) materials and registration formats. Structural readiness was assessed in a total of 23 health centers and 33 health posts.

The second dimension was the process of service delivery. In this dimension we assessed what is done while giving care through direct observation of service provision at difference contact points. The contact points include Integrated Management of Neonatal and Childhood Illness (IMNCI) unit, immunization unit, antenatal care (ANC) unit, delivery and 79 Post-Natal care (PNC) unit. We observed a total of 103 IMNCI, 73 immunizations, 94 ANC and 75 PNC service provision including anthropometric assessment and interpretation, provision of IFA supplementation, provision of nutrition counselling and essential counselling skill of service providers. All observation checklists were prepared using CINS guide prepared by MOH as a base. All tools were prepared in English and then translated into Amharic, then back to English. Tools were pretested and the necessary modification was made prior to actual data collection.

In order to have in depth understanding of nutrition services we have conducted key informant interview. The interview guide contained open ended questions to have high degree of flexibility. The interview guide covered topics on multi-sectoral collaboration, integration of nutrition services, planning of nutrition services, registration and reporting of service provision, supportive supervision, human resource and motivation of service providers. The interview guide was initially developed in English and it was then translated to Amharic.

The date, time and place of interview were arranged following what was most favorable and comfortable for the participants. Interviews were conducted in a quite office and privacy was ensured to enable participant to feel free and express their opinions. All interviews were audio taped and took about an hour on average. All data were collected by the project coordinators assigned in the four districts. All data were reviewed for completeness and consistency.

**Statistical analysis**

We used Epi data version 3.1 for data entry and the statistical software package STATA version 14 for data cleaning and analysis. Descriptive statistics was performed for different components of the nutritional services. Frequency tables and graphs were used to further describe the data set.

To analyze the qualitative data, we initially developed field note template. Using this template, we wrote an expanded field note for each interview. Then we developed analysis matrix where the rows consist the subgroup selected for the study i.e. the respondents and the columns stands for the topic of study (i.e. the question asked). Using this matrix, we then extracted key findings from the field notes. Quotations were used to further illustrate the responses of the respondents on important issues.

**Ethics approval and consent to participate**

Ethical clearance was obtained from the Research Ethical Committee of School of Public Health, Addis Ababa University. In addition, permission was obtained from relevant Federal and local government offices. Written informed consent was obtained from the participants after the necessary explanation about the purpose, procedures, benefits and risk of the study have been made. The respondents’ right to refuse few or all of the questions was respected at all times. Privacy of participants and confidentiality of the information found was kept.

# **Results**

A. **Structural readiness**

**Availability of essential equipment at health facility.** The structural readiness of health centers for nutrition service delivery is presented in table 1. Out of the total health center majority of ANC consultancy room (21/23) were equipped with weighing scale. While only (17/23) and (14/23) IMNCI and immunization room were equipped with weighing scale respectively. Height/length board was available in (16/23) IMNCI and ANC consultation room while only (8/23) immunization rooms were equipped with height/length boards. Mid-upper arm circumference (MUAC) tape was available in (19/23) of ANC and immunization room but it was available in all IMNCI room. Essential counselling materials were found in (10/23) of immunization and IMNCI room and in (8/23) ANC and delivery room. Appropriate registration and reporting format were found in all contact points of health centers.

**Table 1:** **Structural readiness of Health Centers- Availability of equipment, guide lines and record keeping registers.**

|  | Immunization n=23 | IMNCI n=23 | ANC n=23 | Delivery n=23 |
| --- | --- | --- | --- | --- |
| Anthropometric equipment |  |  |  |  |
| Baby weighing scale | 18 | 18 | NA | 23 |
| Adult weighing scale | 14 | 17 | 21 | 14 |
| Height/length board | 8 | 16 | 16 | 0 |
| MUAC tape | 19 | 23 | 19 | 16 |
| Counselling guide | | | | |
| AMIYCN guideline | 10 | 10 | 8 | 8 |
| Registration book | | | | |
| Registration book and reporting format | 23 | 23 | 23 | 23 |

*NA not applicable

The structural readiness of health post for nutrition service delivery is shown in table 2. Majority of health post (32/33) had baby weighing scale, and all health post had MUAC tape. However, only (20/33) and (8/33) had adult weight scale and height scale respectively. Adolescent, Maternal, Infant and Young Child Nutrition (AMIYCN) guideline was found only in (9/33) health post and demonstration equipment was found only in (6/33) health post. ANC registration book was found in (27/33) health post while PNC registration was found only in (15/33) health post.

**Table 2: Structural readiness of Health Posts- Availability of equipment, guide lines and record keeping registers.**

| Equipment (n=33) | Available and functional | Not available |
| --- | --- | --- |
| Baby weighing scale | 32 | 1 |
| Adult weighing scale | 20 | 13 |
| Height/length board | 8 | 25 |
| MUAC tape | 33 | - |
| AMIYCN guideline | 9 | 24 |
| SAM guideline | 19 | 14 |
| Demonstration equipment | 6 | 27 |
| Child card/record | 23 | 10 |
| ANC Registration book | 27 | 6 |
| PNC registration book | 15 | 18 |
| Reporting format | 30 | 3 |

Table 3 shows availability of medical supplies for nutrition service among observed health centers and health posts. Vitamin A was available in (21/23) health center and (30/33) health posts. Iron folic acid (IFA) supplementation was available in all health centers however, two health posts did not have IFA at the time of observation.

**Table 3: Availability of drug supply for nutrition services at health centers and health posts.**

| List of drugs | Health center (n=23) | Health post (n=33) |
| --- | --- | --- |
| Albendazole | 17 | 29 |
| Vitamin A | 21 | 30 |
| RUTF F-75 | 10 | NA |
| RUTF F-100 | 8 | NA |
| RUSF | 16 | 20 |
| Zinc | 23 | 30 |
| ORS | 23 | 32 |
| Daily rations (TSF) | 1 | 7 |
| Antibiotics | 23 | 33 |
| Iron folic acid | 23 | 31 |

*NA not applicable

B. **Process evaluation**

**Anthropometric assessment.** Evaluation of nutritional service delivery was assessed by observing a total of 103 IMNCI, 73 immunizations, 94 ANC and 75 PNC service provision. Result from observation of anthropometric assessment in IMCNI and immunization rooms is presented in figure 1. During provision of IMNCI service 62 (60.2%) children had weight measured, 38 (36.9%) had their height and 60 (58.3%) children had their MUAC measured. Of those children whose anthropometric measurement was taken in IMNCI, correct interpretation was done for 32 (51.6%) children using weight for age z-score and for 28 (73.7%) children using height for age Z-score. Correct interpretation using MUAC cutoff point was done for 39 (65%) of children. During provision of immunization service, we have observed only 12 children had their weight measured. However, none of the children who came for immunization service had their height measured.

Assessment of nutritional service delivery for mothers was determined by observing a total of 94 ANC and 75 PNC service provision. During ANC service a total of 75 (79.8%) mother had their weight measured and 41 (43.6%) mother had their MUAC measured. However, only 15 (15.96%) mother had their height measured. Of those mothers whose MUAC was measured, correct interpretation using MUAC cut off was done for 34 (82.93%) of the mothers. Out of 75 mothers who were measured for their weight, the service provider commented about the weight of 33 (44%) of the mother.

**Nutrition counselling provision.** Table 4 shows different nutrition counselling given for mothers who came for IMNCI service. Out of 31 mothers with children under six months of age who visited IMNCI, a total of 19 (61.3%) were counselled on exclusive breastfeeding for the first six months. Of the total number of children illegible for counselling about initiation of complementary feeding only 13 (41.9%) mothers were counselled about initiation of complementary food. As indicated in the table only 15 (14.6%) were counselled on hand washing practice and 10 (9.7%) were counselled on proper storage of food.

**Table 4: Proportion of mothers receiving nutrition counselling during IMNCI service provision**

| Nutritional counselling during IMNCI service (n=103) | Frequency | Percent |
| --- | --- | --- |
| Counselling on breastfeeding |  |  |
| Advised on Exclusively BF for 6 first months (n=31) | 19 | 61.3 |
| Advised on Continuing frequent, on-demand breastfeeding until 2 years of age (n=84) | 27 | 32.1% |
| Counselling on complementary feeding | | |
| Advised on initiation of CF at 6 months (n=31) | 13 | 41.9% |
| Advised on the practice responsive feeding (n=103) | 18 | 17.5% |
| Advised on increasing the frequency as the child gets older (n=72) | 15 | 20.8% |
| Advised on gradually increasing food consistency as the infant gets older (n=72) | 16 | 22.2% |
| Advised on diversity to feed a child from grain, roots and tubers (n=72) | 25 | 34.7% |
| Advised on diversity to feed a child from legumes and nuts (n=72) | 23 | 31.9% |
| Advised on diversity to feed a child from fruits and vegetables (n=72) | 18 | 25% |
| Advised on diversity to feed a child from animal source foods (n=72) | 15 | 20.8% |
| Advised on increasing fluid intake during illness, frequent breastfeeding, and encourage the child to eat soft, varied, appetizing, favorite foods (n=103) | 26 | 25.2% |
| Counselling on WASH | | |
| Advised on personal and Food Hygiene (hand washing before food preparation, after vising toilet, during feeding a child (n=103) | 15 | 14.6% |
| Proper storage of food (n=103) | 10 | 9.7% |

Out of 68 mothers with children under six months of age who visited immunization unit, only 11 (16.2%) were counselled to exclusively breastfeed their child for six months. Following this a total of 9 (13.4%) mothers were counselled about initiation of complementary feeding at 6 months. During our observation at immunization unit, there were only 5 children who were above the age of six months and no nutritional counselling was given for theses mothers (Table 5).

**Table 5: Proportion of mothers receiving nutrition counselling during immunization service provision**

| Nutritional counselling during Immunization service (n=73) | Frequency | Percent |
| --- | --- | --- |
| Counselling on breastfeeding |  |  |
| Advised on Exclusively BF for first 6 months (n=68) | 11 | 16.2% |
| Advised on Continuing frequent, on-demand breastfeeding until 2 years of age (n=73) | 5 | 6.9% |
| Counselling on complementary feeding | | |
| Advised on initiation of CF at 6 months (n=68) | 9 | 13.2% |
| Advised on the practice responsive feeding (n=73) | 3 | 4.1% |
| Advised on increasing the frequency as the child gets older (n=5) | 0 | 0 |
| Advised on gradually increasing food consistency as the infant gets older (n=5) | 0 | 0 |
| Advised on diversity to feed a child from grain, roots and tubers (n=5) | 0 | 0 |
| Advised on diversity to feed a child from legumes and nuts (n=5) | 0 | 0 |
| Advised on diversity to feed a child from fruits and vegetables (n=5) | 0 | 0 |
| Advised on diversity to feed a child from animal source foods (n=5) | 0 | 0 |
| Advised on increasing fluid intake during illness, frequent breastfeeding, and encourage the child to eat soft, varied, appetizing, favorite foods (n=73) | 4 | 5.48% |
| Counselling on WASH | | |
| Advised on personal and Food Hygiene (hand washing on before food preparation, after vising toilet, feeding child (n=73) | 4 | 5.5% |
| Proper storage of food (n=73) | 1 | 1.4% |

Table 6 shows different nutrition counselling given for mothers who came for ANC follow up. A total of 74 (78.7%) mothers were counselled on having one extra meal every day. While 46 (48.9%) mothers were counselled on having variety of food and 39 (41.5%) were counseled on the importance of having variety of food. Counselling on what to avoid such as eating raw food and what to limit such as caffeine was given for only for 4 and 3 mothers respectively. We have also observed that only 18 (19.2%) of the mothers were counselled on maternal complication of under nutrition.

**Table 6: Proportion of mothers receiving nutritional counselling during antenatal care service provision**

| Nutritional counselling (n=94) | Frequency | Percent |
| --- | --- | --- |
| Advised the mother to have one extra meal/ snack every day | 74 | 78.7% |
| Advised the mother to eat from variety of food groups using locally available food | 46 | 48.9% |
| Advised the mother about the importance of eating variety of food groups | 39 | 41.5 % |
| Advised the mother about having seasonal fruits and vegetables everyday | 19 | 20.2% |
| Advised the mother to have adequate fluid intake (*at least 8 glasses of fluid everyday*) | 29 | 30.9% |
| Advised the mother about adding iodized salt when serving food (after cooking) | 13 | 13.8% |
| Advised the mother what to avoid during pregnancy *(i.e. Alcohol, raw/uncooked foods and vegetables)* | 4 | 4.3% |
| Advised the mother what to limit during pregnancy (*i.e. caffeine, fat )* | 3 | 3.2% |
| Advised the mother about maternal complications of under nutrition during pregnancy | 18 | 19.2% |
| Advised the mother about fetal complications of under nutrition during pregnancy | 20 | 21.3% |
| Advised the mother on the importance of early initiation of breast feeding (fourth visit) (n=21) | 4 | 19% |
| Advised the mother on importance of feeding colostrum (fourth visit) (n=21) | 1 | 4.8% |
| Advised the mother to avoid pre-lacteal feeding (fourth visit) (n=21) | 3 | 14.3% |
| Advised the mother to expose the child to direct sun light (fourth visit) (n=21) | 1 | 4.8% |
| Advised the mother about the importance of exclusive breast feeding for six month (fourth visit) (n=21) | 8 | 38.1% |
| Counselling on WASH (n=94) | | |
| Advised the mother to use treated water for drinking | 3 | 3.2% |
| Advised the mother to wash hand after toilet use, and before meal preparation | 11 | 11.7% |
| Advised the mother to keep utensils and cooking material clean | 9 | 9.6% |
| Advised the mother to keep personal hygiene | 46 | 48.9% |
| Counselling on ITN (n=94) | | |
| Advise on ITN use | 34 | 36.2% |

Figure 2 shows counselling given on IFA for mothers during ANC follow up. Out of the total mothers who came for ANC, IFA was provided for 85 (90.4%) mothers. Of those who had been prescribed IFA, 73 (85.9%) were counselled to take IFA daily for six months. However, only 49 (57.7%) mothers were counselled about the benefit of IFA and less than half 36 (42.4%) were counselled on the possible side effect of IFA.

Table 7 shows different nutrition counselling given for mothers who came for PNC service. We have observed 75 (94.9%) mothers were counselled to exclusively breastfeed up to six months. Regarding maternal nutrition, 63 (79.8%) mothers were counseled to have two extra meal and rest during lactation. Counselling about maternal complication of undernutrition was given for only 27 (34.18%) mothers. The least counselling service given for mothers was counselling about having seasonal fruits and vegetables which was 14 (17.7%).

**Table 7: Proportion of mother receiving nutritional counselling during postnatal care service provision**

| **Nutritional counselling (n=79)** | Frequency | Percent |
| --- | --- | --- |
| Advised the mother about extra meal and rest during lactation | 63 | 79.8% |
| Advised the mother to eat a diet that is rich in protein and fluid | 38 | 48.1% |
| Advised the mother to eat food rich in iron | 42 | 53.2% |
| Advised mother to avoid all dietary restriction if any | 35 | 44.3% |
| Advised mother to exclusively breastfed up to six month | 75 | 94.9% |
| Advised the mother on the importance of exclusive breast feeding for six month | 56 | 70.9% |
| Advised mother to breast fed on demand | 62 | 78.5% |
| Advised mother on correct positioning and attachment during breast feeding | 54 | 68.4% |
| Advised the mother to eat from variety of food groups using locally available food | 48 | 60.8% |
| Advised the mother on the importance of eating variety of food groups | 37 | 46.8% |
| Advised the mother to have seasonal fruits and vegetables everyday | 14 | 17.7% |
| Advised the mother to have adequate fluid intake (at least 8 glasses of fluid everyday) | 21 | 26.6% |
| Advised the mother about adding iodized salt when serving food (after cooking) | 13 | 16.5% |
| Informed the mother about maternal complications of under nutrition during lactation | 27 | 34.2% |
| Counselling on WASH (n=79) | | |
| Advised the mother to use treated water for drinking | 15 | 18.9% |
| Advised the mother about safe disposal of potentially infectious soiled pads or other materials | 14 | 17.7% |
| Advised the mother to wash hand after toilet use, and before meal preparation, after cleaning baby, before feeding ,before breast feeding | 12 | 15.2% |
| Advised the mother to keep utensils and cooking material clean | 15 | 18.9% |
| Advised the mother to keep personal hygiene | 40 | 50.6% |

**Counselling skill of service providers.** Counselling skill of service providers was assessed at different contact points. We have found that 94 (91.2%) of service providers at IMNCI, 55 (75.3%) at immunization, 92 (97.9%) at ANC and 71 (89.9%) at PNC listen to what the clients have to say (Table 8).

As indicated in the table, 84 (81.6%) of service providers at IMNCI, 54 (73.9%) at immunization, 92 (97.9%) at ANC and 71 (89.9%) at PNC used responses and gesture that shows interest. However, praising a client for something they have done right was not common. Praising of clients was the highest among providers at ANC 43 (45.7%) followed by providers at PNC 25 (31.7%). This practice was the lowest at immunization unit where only 7(9.6%) service providers praised the clients for something they have done right.

During counselling service provision, providers identified key difficulties for 38 (40.4%) of client at ANC followed by 21 (20.4%) and 13(16.5%) at IMNCI and PNC respectively. Out of these, providers had discussed about options for the identified difficulties with 28 (73.7%), 17 (80.9%) and 12 (92.3%) at ANC, IMNCI and PNC unit respectively.

**Table 8: Counselling skill of service providers during provision of counseling at different contact points**

| Counselling | IMNCI (n=103) | Immunization (n=73) | ANC (n= 94) | PNC (n=79) |
| --- | --- | --- | --- | --- |
| Service provider listens to what the client has to say | 94 (91.3%) | 55 (75.3%) | 92 (97.9 %) | 71 (89.9%) |
| Service provider used responses and gestures that show interest | 84 (81.6%) | 54 (73.9%) | 89 (94.7%) | 71 (89.9%) |
| Service provider let the client talk through her concerns before correcting information | 59 (57.3%) | 31 (42.5%) | 84 (89.4%) | 63 (79.8%) |
| Service provider avoided using judging words | 84 (81.6%) | 52 (71.2%) | 83 (88.3%) | 63 (79.8%) |
| Service provider recognized and praised what the client is doing correctly | 24 (23.3%) | 7 (9.6%) | 43 (45.7%) | 25 (31.7%) |
| Service provider Identified key difficulties (if any) and selects with the client the most important one to work on | 21 (20.4%) | 5 (6.8%) | 38 (40.4%) | 13 (16.5%) |
| Of those mothers with key difficulties service provider discussed options on key difficulties the client raised | 17 (80.9%)^*^ | 5 (100%)^*^ | 28 (73.7%)^*^ | 12 (92.3%)^*^ |
| Service provider recommends and negotiates do-able actions to help the client select the best option to try depending on her context and resources | 16 (76.2%)^*^ | 5 (100%)^*^ | 26 (68.4%)^*^ | 10 (76.9) ^*^ |
| Service provider helped the client agree to try one of the options and asks them to repeat the agreed-upon do-able action | 15 (71.4%)^*^ | 5 (100%)^*^ | 26 (68.4%)^*^ | 9 (75%)^*^ |
| Service provider recorded history of the client | 87 (84.5%) | 51 (69.9%) | 71 (70.9%) | 56 (70.9%) |

^*^ The denominator is the number of clients identified with key difficulties

**Qualitative evaluation**

**Participant profile**

A total of 60 participants in the four districts took part in this study. The participants include district administrative head, district health office head, deputy district health office head, district nutrition focal person, program officers, service providers at health center, religious leader, WDA, HEW and PHCU head.

**Multi-sectoral collaboration**

There is existing Woreda nutrition coordination body (WNCB), Woreda nutrition technical committee (WNTC) and Kebele nutrition technical committee (KNTC) in all district. These structures were formed following 1000 days plus program launching. Key informant interview revealed that even though there is existing structure it is not functioning to the level of expectation. Neither the coordination body nor the technical committee meet regularly. They rarely meet to the point where the structure is almost non-functional. One of the key informants explained about the functionality of WNCB, WNTC and KNTC as follow:

*" The WNCB meetings are not conducted regularly. There is usually third body request to have the meeting. It is not scheduled ahead and usually done hastily. There is unequal commitment of sectors, not planning together and there are no terms of reference. I can say WNTC almost does not exist and needs reestablishment. Regarding KNTC; the HEWs have capacity gap, they are not committed and motivated because of carrier structure problem, inadequate salary, no recognizing/rewarding mechanism, work overload etc. The kebele leaders are not committed as well.” (30 years old, male, BSc Nurse)*

The functionality of the structure is mainly dependent up on the existence of different projects in the district to activate them. Key informants believe that having WNCB is very important for the success of nutrition services. However, there is lack of ownership among different members. A health officer explained:

*“There is a tendency of pushing this job aside by the health sector due to different emerging activities and other priorities. And the other sectors are dependent on an external initiation and they don’t own this structure as part of their routine job. Apart from this there is also lack of logistic to conduct the meeting effectively…. you now like incentives.” (32 years old, male, health officer)*

The participants of this study also mentioned that the members in the coordination body externalize their responsibility to the health sector and there is an assumption that nutrition is the concern of health sector only. One participant expressed the following:

*“Because of other political priorities the coordination body is not operating to the envisioned extent. So mainly it is the technical committee which is working better currently. Though there are certain limitation on the technical committee as well. There is a high challenge with ownership of the body as they feel this task is solely the responsibility of the health sector”. (48 years old male BSc Nurse)*

**Integration of nutrition services**

Our finding shows there is poor integration of nutrition services. There is a physical rather than functional integration of nutrition service. For instance, growth monitoring and promotion (GMP), immunization and IMNCI service are given in one room usually by one service provider. However, there is missed opportunity of providing GMP for children who came for immunization and IMNCI service.

*“There is no separate room for GMP, IMNCI and immunization at health center level. These three activities are offered in a room by a provider. The same is true for ANC, Delivery and PNC …. However; service providers are not using this opportunity to give nutrition service in an integrated manner.” (30 years old male, MPH)*

**Human resource, training and motivation**

We have assessed staff adequacy, training and motivation of service providers at different levels in the districts. According to our finding there is critical staff shortage at each level starting from district to health post. This staff shortage was more profound at remote health facilities.

*" I myself focal for three positions, work in regular and emergency outpatient department (OPD), the health center head also works in OPDs, other staffs also have burden and generally there is staff shortage; there has been very critical shortage though yesterday one health officer joined the health center. There are only 5 staffs including the head and me. There is shortage of staffs in all departments."(27 years old, male, Nurse)*

Even though there is shortage at health centers, it was significant at health post level compared to health centers. The number of the HEWs was not comparable with the number of people living in their catchment area. One participant explained:

*“Based on the standard one health extension worker gives service to 2500 populations in her catchment area (1:2500). But the ratio in our woreda is 1: 10,000 to 11,000 populations. This is similar in the health center as well, there is not enough service provider compared to service seekers.” (32 years old, male, Health officer, Offa district)*

Besides staff shortage there was also high staff turnover. Similar to staff shortage the turnover was more prominent in rural health facilities. The turnover was most common among midwives, health officers and HEW.

*“This year 8 HEWs left their job and moved to different cities by changing their job. It became a big challenge for the woreda. Some health posts have only one HEW. Currently Zemo health post have no HEW as a result it is closed.” (30 years old, male, BSc Nurse)*

We have identified training gaps in AMIYCN, out-patient therapeutic program (OTP), community-based nutrition (CBN), management of sever acute malnutrition, blended integrated nutrition and counselling skill. Besides, the training opportunities mainly depend on the partner’s or training provider organization’s objective rather than the need of the health sectors in the district. Furthermore, we have identified poor opportunities for health care providers to further their education and future career development.

*" …there is no study opportunity. As I mentioned for you earlier, I joined this health center nearly five years back. It is very difficult to talk on this and there is no chance to pursue our education. Even when there are opportunities, there is no fair selection of candidates." (27 years old, male, nurse)*

We also evaluated motivation of service provider. We have found poor motivation among service providers. Participants described variety of factors affecting their motivation including access to training, access to further education, timely duty payment, work overload, and inadequate salary.

**Planning**

There is five-year strategic health sector plan. This plan is being used as a baseline for the district plan. During planning HEWs, health center heads, District health office personnel and other delegates from other sectors such as children and women affairs, agriculture, finance etc. will usually be involved. However, the planning followed top to bottom approach. This approach does not take in to account the existing situation on the ground. Because of this, service providers had to deal with numbers sent from the top level officials which doesn’t really exist in their catchment area. They have explained this as follows:

*"We are facing challenge during planning as we breakdown the figures we took from the woreda to each health post by using conversion factors. Therefore, the plan is not based on the existing situation of the PHCU."(26 years, male, health officer)*

*“There was a time where they give us 5000 plans while we have 3000 in our catchment and vice versa so don’t get surprised when you see 205% achievement.” (28 years, male, B.sc Nurse)*

**Registration and reporting**

There were different registration books used by professionals for different services. Registration format should be available at each facility but our study showed there was shortage of registration books especially at health post level.

*“We are using self-made registry This is a burden for us since making the register is tiresome. It also has drawback as we might omit some important variables”. (32 years, female, HEW)*

The other challenge with registration book was related with portability. Health extension workers faced difficulty to take registration book during outreach activities as a result they used a plain paper during outreach services. This has drawback as the HEW often forget to copy to the registers resulting in poor documentation and inconsistency among reports and registers. In general, there is poor documentation especially at health post level.

*"There is no documentation problem at HC level. We perform activities and record accordingly......... but at HP level there is gap. Last time I and the health center head along with kebele leader came across with poor documentation. This might be because the HEW are newly assigned since senior HEWs have left their job for different reasons."* (*27 years, nurse)*

Similar to registration, we have identified challenges related with reporting as well. The challenges were lack of timeliness, reliability, and completeness of the reports.

*“There was an incident where one of the health extension worker sent 0 ANC on the HMIS report and 20 ANC on the disease report, so we will cross check for such kind of errors and give feedback on how to improve the report in the future”. (28 years, male, Bsc Nurse)*

**Supportive supervision**

The district health office provides supportive supervision to the health centers and health centers provide supportive supervision to health posts. There were two forms of supportive supervision in the district; integrated supportive supervision and program specific supportive supervision. As the name implies, integrated supportive supervision was conducted every quarter by integrating different case team leaders from the district health office. The program specific supervision was done whenever the need arises by individual assigned to a specific program. This type of supervision focuses on specific programs.

*“There is Integrated Supportive Supervision(ISS) this will be done every quarter where different officers from the Woreda health office supervise health centers, health posts and at least 10 sample households at community level….”. (43 years, Male, B.sc Nurse)*

# **Discussion**

The landscape analysis was intended to assess service readiness and nutrition service provision at different contact points. The result from this study indicated poor structural readiness and gap in nutrition services provision. Health facilities lacked essential anthropometric instruments, nutrition guide lines, registration and reporting formats. Poor structural readiness was more prominent at health post levels compared to health centers. Our process evaluation indicated that there is missed opportunity of anthropometric assessment and preventive nutrition counselling at different contact points. Such problem was more critical at immunization unit than other units. In our qualitative assessment, we found that there is an existing but non-functional Woreda nutrition coordination body and technical committee. Training gaps, staff shortage and high staff turnover were the main challenges faced by service providers resulting in burden, fatigue and poor motivation among providers.

We have observed poor structural readiness in both health center and health posts. However, overall facility preparedness was better in health center compared to health post. This might be because the health centers provide comprehensive primary health care including promotive, curative and rehabilitative services as compared to health posts. Even though health centers exhibited better structural readiness, the level of readiness varied across different units. For instance, there were greater availability of weighing scale at ANC units than other units. Such type of problems are also common in other countries like Bangladesh where ANC rooms have better structural readiness than other units [17]. This might be because ANC has been practiced well as part of the health service implementation compared to the recently introduced nutrition service implementation in IMNCI [17].

Integration of nutrition service is expected at different contact points such as with IMNCI. It is assumed that if screening is performed routinely in curative clinics, it creates a better opportunity for timely detection and intervention. However, our assessment of service provision in IMNCI indicated a missed opportunity for basic nutrition screening and counselling. Indeed, of total number of children who came for IMNCI service, only 60.2% had their weight measured. It is important to note that the current IMCNI algorithm asks for weight of the child and yet 40% of the children were not weighed. Similarly, a study done in Botswana showed limited nutrition and dietary screening in curative clinics [18]. This missed opportunity could be attributed to lack of anthropometric, poor motivation of staffs, shortage of staff and lack of supervision.

Poor interpretation of anthropometric assessments were also the findings from this study. Without nutrition assessment and correct interpretation, it is unlikely there would be appropriate management of undernutrition and appropriate treatment of other childhood illness [19]. Of those children whose weight was measured at IMNCI unit, correct interpretation of weight for age Z-score was done for only 51.6% of the children. This might be related with capacity gaps, poor commitment and work load among service providers.

The other challenge that we identified was related to provision of preventive services such as proper counselling during clinical contact. Poor nutrition counselling could be a result of high case load, staff shortage and capacity gap which led to prioritization of the immediate clinical concern rather than focusing on preventive intervention. However, overall nutrition service at IMNCI was relatively better as compared to immunization unit. We have identified a critical missed opportunity at immunization unit where only 16.4% of children had their weight measured while none of the children had their height measured. Lack of essential anthropometric instruments such as weight scale and stadiometer could have been possible reason for lack of appropriate nutritional assessment. Besides, lack of essential equipment, high case load during immunization day could also be the possible reason for missed opportunity. The trend of provision of immunization was using a fixed day resulting in high case load making immunization one of the most rushed services. This has resulted in poor nutrition counseling service at immunization unit. Of all the services, the least preventive counselling service was observed in this unit.

Immunization services are often widely available and potentially can support and be supported by additional health interventions. The combined delivery or integration of linked health interventions including GMP, nutrition advises and preventive cares for a more effective way of achieving common health goals [20]. In the study area there was an opportunity to integrate nutrition services as there was a physical integration of IMNCI, Immunization and GMP services. These services were given in a single room and by a single provider in most of the health facilities. However, the service provision did not progress beyond the physical integration which resulted in missed opportunities.

The level of provision of essential nutrition services was better at ANC and PNC unit as compared to IMNCI and immunization unit. This is reflected by improved nutrition counselling at ANC and PNC unit. Nutritional assessment was also better at ANC unit. For instance, majority (79.79%) of the mothers at ANC were weighed. We have also observed a relatively better counselling at ANC unit. This improved nutrition service might be due to the preventive nature of ANC service. Since ANC service is preventive than curative it makes it conducive to provide nutrition counselling than other services.

The coverage of IFA supplementation for mothers who came for ANC was 90.7%. Even though supplementation coverage was high, counselling related to IFA was very poor. Only 57% of the mothers were counselled on benefit of IFA while only 42.4% were counselled on possible side effect. This finding was similar with a report conducted with the aim of describing and comparing nutrition intervention using service readiness and delivery across different countries. The finding from the study indicated prescription/provision of IFA was higher than counselling service related with IFA. Prescription ranged from 62.2% in Nepal to 87.5% in Senegal. Nonetheless, counselling on benefit of IFA ranged from 18% in Nepal to 72.6% in Tanzania. Counselling on possible side effect was very low ranging from 1.8% in Senegal to 14.2% in Tanzania [21]. Inadequate tools and skill in counselling to support and monitor adherence could be possible reason for the observed result [22].

Poor counselling service about benefit, possible side effect, severity and magnitude of the problem has been identified as barriers for non-adherence of IFA [21,23–25]. A systematic review has identified fear of side effect as the commonest reason for non-adherence which was 46.4% [26]. As a result, counselling addressing benefit and possible side effect with mitigation strategy to overcome it should be incorporated other than mere provision of the supplement to improve adherence.

During our assessment, we have identified major challenges faced by service providers. For instance, training gap, staff shortage and high staff turnover were the commonly identified gaps. The training gap was not uniform in all unit; it was more pronounced for health care providers at maternity unit. The other challenge with training is that it was not need based and mainly depend on the objective of a partner organization. Similar to training, staff shortage and turnover was also not uniform. It was more common at rural health facilities. This might be due personal reasons and service providers seek to live in town or near to town. such problems could be possible reasons for poor implementation of services at different unit.

Motivation of staff was also found to be one factor for effective delivery of nutrition services. Health extension workers are overloaded with health and other non-health related commitments such as political tasks which has resulted in poor motivation and fatigue of health extension workers. Apart from the burden of work, lack of access for trainings, timely duty payment, national career structure and inadequate salary were factors for poor motivation of service providers.

Because of the multi-sectoral nature of nutrition, various stakeholders outside the health needs to be involved, coordinated and linked to gain the synergistic impact for achievement of nutrition interventions [6]. Even though nutrition coordinating bodies and technical committees were established in all the districts, it is not functioning to the desired level. The primary reason for this could be engagement of different sectors in other urgent and political commitments. Even though the sectors will be housed in health sector all member of the coordinating body and technical committee should be equally accountable [27]. However, this was not the reality in all districts. There was poor ownership and accountability among sectors reflected by externalizing every responsibility to the health sector. This might be a result of lack of strong monitoring and evaluation system in place. In addition, lack of coordination from the federal and regional governments has been identified as a constraint to improve nutrition service delivery in Ethiopia [28]. In order to ensure functionality of coordination body and technical committee, there should be a mechanism to capture data and triangulate from all relevant sectors.

We believe the current study has limitation that needs to be acknowledged. Since we used direct observation it is difficult to rule out desirability bias. Direct observation during service provision may have encouraged service providers to offer a more thorough service for their clients. However, we believe the impact is minimal since our finding on both the structural readiness and process evaluation were low. The strength of this study was the assessment of both health centers and health posts and the use of multiple methods of data collection techniques including observation, and key informant interviews to assess the structural readiness and provision of nutrition services.

# **Conclusion and Recommendation**

There is poor structural readiness and gaps to deliver integrated nutrition services in all districts. There was high rate of missed opportunities for different nutrition services including nutrition screening and counselling. There is also poor commitment and accountability of coordination body and technical committee towards nutrition intervention. Improving logistics and training may improve delivery of nutrition service. Improving man power might minimize missed opportunity as a result of high case load. It might also ensure adequate time to provide thorough preventive counselling service at all units. Ensuring a clear structure of accountability along with reporting mechanisms in the existing system might ensure functionality and accountability of the coordinating body and technical committee.

# **Acknowledgment**

We are very grateful for the financial support of Irish Aid Ethiopia and of Ministry of Health of Ethiopia. We would like to thank Addis Ababa University, School of Public Health for facilitating the conduct of the research. We appreciate the managers and health care providers for their assistance and cooperation during the assessment. Finally, we would like to thank all study participants who took part in this study.

# **Reference**

1.	Black RE, Allen LH, Bhutta ZA, Caulfield LE, de Onis M, Ezzati M, et al. Maternal and child undernutrition: global and regional exposures and health consequences. Lancet. 2008;371(9608):243–60.

2.	Black RE, Victora CG, Walker SP, Bhutta ZA, Christian P, De Onis M, et al. Maternal and child undernutrition and overweight in low-income and middle-income countries. Lancet. 2013;382(9890):427–51.

3.	World Health Orgnization. Nutrition in the WHO African Region.

4.	UNICEF, WHO World Bank Group and United, Levels and Trends in Child Mortality Report, 2017.

5.	Ethiopia Demographic and Health Survey 2016 Central Statistical Agency Addis Ababa, Ethiopia ICF International Calverton, Maryland, USA, 2017.

6.	Federal Democratic Republic of Ethiopia - National Nutrition Program, 2016-2020.

7.	Bhatia J, Bhutta ZA, Kalhan SC, editors. Maternal and child nutrition: the first 1,000 days. Karger Medical and Scientific Publishers; 2013.

8.	RE Black, J Hopkins Bloomberg The lancet series on maternal and child nutrition, executive summary, Lancet 372, 1-10, 2008.

9.	Karakochuck CD, Whitfield KC, Green TJ, Kraemer K. The biology of the first 1,000 days. CRC Press; 2017 Sep 22.

10.	Schwarzenberg SJ, Georgieff MK. Advocacy for improving nutrition in the first 1000 days to support childhood development and adult health. Pediatrics. 2018;141(2).

11.	Beluska-Turkan K, Korczak R, Hartell B, Moskal K, Maukonen J, Alexander DE, et al. Nutritional gaps and supplementation in the first 1000 days. Nutrients. 2019;11(12):1–50.

12.	Bellieni C V. The Golden 1,000 Days. J Gen Pract. 2016;04(02):2–5.

13.	Maalouf-Manasseh Z, Oot L, Sethuraman K. Food and Nutrition Technical Assistance III Project Giving Children the Best Start in Life: Integrating Nutrition and Early Childhood Development Programming within the First 1,000 Days. SecurenutritionOrg [Internet]. 2016;(January).

14.	Lowensohn RI, Stadler DD, Naze C. Current concepts of maternal nutrition. Obstetrical & gynecological survey. Pediatr Emerg Care. 2017;33(12):792–3.

15.	Wu G, Bazer F, Cudd T, Meininger C. Recent Advances in Nutritional Sciences-Maternal Nutrition and Fetal Development. Nutr [Internet]. 2004;(13):2169–72.

16.	Abu-Saad K, Fraser D. Maternal nutrition and birth outcomes. Epidemiol Rev. 2010;32(1):5–25.

17.	Billah SM, Saha KK, Khan ANS, Chowdhury AH, Garnett SP, Arifeen S El, et al. Quality of nutrition services in primary health care facilities: Implications for integrating nutrition into the health system in Bangladesh. PLoS One. 2017;12(5):1–16.

18.	Nnyepi MS. Dietary and Nutrition Screening for Children Seeking curative care in health facilities in Botswana. African Journal of Food, Agriculture, Nutrition and Development. 2006;6(2).

19.	Arifeen SE, Bryce J, Gouws E, Baqui AH, Black RE, Hoque DME, et al. Quality of care for under-fives in first-level health facilities in one district of Bangladesh. Bull World Health Organ. 2005;83(4):260–7.

20.	World Health Orgnization. GIVS: Global Immunization Vision and Strategy 2006-2015. World Health Organization;2005.

21.	Wang W, Benedict RK, Mallick L. The Role of Health Facilities in Supporting Adherence to Iron-Folic Acid Supplementation During Pregnancy: A Case Study Using DHS and SPA Data in Haiti and Malawi. DHS Working Paper No.160. Rockville, Maryland, USA: ICF. 2019;(August).

22.	Siekmans K, Roche M, Kung’u JK, Desrochers RE, De-Regil LM. Barriers and enablers for iron folic acid (IFA) supplementation in pregnant women. Matern Child Nutr. 2018;14(July 2017):1–13.

23.	Taye B, Abeje G, Mekonen A. Factors associated with compliance of prenatal iron folate supplementation among women in Mecha district, Western Amhara: A cross-sectional study. Pan Afr Med J. 2015;20:1–7.

24.	Assefa H, Abebe SM, Sisay M. Magnitude and factors associated with adherence to Iron and folic acid supplementation among pregnant women in Aykel town, Northwest Ethiopia. BMC Pregnancy Childbirth. 2019;19(1):1–8.

25.	Arega Sadore A, Abebe Gebretsadik L, Aman Hussen M. Compliance with iron-folate supplement and associated factors among antenatal care attendant mothers in Misha District, South Ethiopia: Community based cross-sectional study. J Environ Public Health. 2015;2015:1–7.

26.	Desta M, Kassie B, Chanie H, Mulugeta H, Yirga T, Temesgen H, et al. Adherence of iron and folic acid supplementation and determinants among pregnant women in Ethiopia: A systematic review and meta-analysis. Reprod Health. 2019;16(1):1–14.

27.	Government of the Federal Democratic Republic Of Ethiopia, Program Implementation Manual of National Nutrition Program ( NNP ) - I July 2008 – June 2013 Federal Ministry of Health. 2013;(June).

28.	Sustainability S, Nutrition OF, Assessment IN, Program S. Programming and Integrating Nutrition Assessment ,. 2018;
